# Mixing and Matching of Hybrid Megasynthases is a Hub for the Evolution of Metabolic Diversity in Cyanobacteria

**DOI:** 10.1002/anie.202502461

**Published:** 2025-05-19

**Authors:** Keishi Ishida, Qi Sun, Jonna Teikari, Christian Hertweck, Elke Dittmann, Masahiro Murakami, Martin Baunach

**Affiliations:** ^1^ Institute for Pharmaceutical Biology University of Bonn Nussallee 6 53115 Bonn Germany; ^2^ Biomolecular Chemistry Leibniz Institute for Natural Product Research and Infection Biology Hans Knöll Institute Beutenbergstr. 11a 07745 Jena Germany; ^3^ Department of Marine Biochemistry Graduate School of Agricultural Life Sciences The University of Tokyo 1‐1‐1 Yayoi Bunkyo‐ku Tokyo 113‐8657 Japan; ^4^ Institute for Atmospheric and Earth System Research University of Helsinki P.O. Box 56 (Viikinkaari 9) Helsinki FI‐00014 Finland; ^5^ Institute of Microbiology Friedrich Schiller University Jena Neugasse 25 07743 Jena Germany; ^6^ Department of Microbiology Institute for Biochemistry and Biology University of Potsdam Karl‐Liebknecht‐Str. 24/25 14476 Potsdam‐Golm Germany

**Keywords:** Biosynthesis, Megasynthase, Molecular evolution, Nonribosomal peptide, Polyketide

## Abstract

Modular megasynthases, such as polyketide synthases (PKSs) and nonribosomal peptide synthetases (NRPSs), are molecular assembly lines that biosynthesize many pharmaceutically and ecologically important natural products. Understanding how these compounds evolve could inspire the artificial evolution of compound diversity by metabolic engineering. Over the past two decades, a number of seminal studies have significantly contributed to our understanding of natural product evolution. However, the evolution of NRPS and PKS assembly lines remains poorly understood, especially for NRPS/PKS hybrids. Here, we provide substantial evidence for a remarkable cluster‐mixing event involving three cyanobacterial biosynthetic gene clusters (BGCs), resulting in the emergence of novel peptide‐polyketide hybrids that were named minutumamides. By combining retro‐evolutionary analysis with structure‐guided genome mining, we could discover a potential evolutionary ancestor that links nostopeptolide and minutumamide biosynthesis. In addition, we were able to trace nostopeptolide‐related module and domain blocks in various other biosynthetic pathways, indicating a surprisingly vivid mixing and matching of biosynthesis genes in the evolution of NRPS and *cis*‐acyltransferase PKS/NRPS pathways, which was previously regarded as a unique feature of *trans*‐acyltransferase (*trans*‐AT) PKS. These remarkable insights into the evolutionary plasticity of NRPS‐PKS assembly lines provide valuable guidance for pathway engineers looking for productive combinations that yield “nonnatural” hybrid natural products.

## Graphical abstract

Natural combinatorics: Evolutionary mixing and matching of three cyanobacterial biosynthetic gene clusters resulted in the emergence of minutumamides, a novel family of peptide‐polyketide hybrids. Retro‐evolutionary analysis led to the discovery of nostopeptolide KVJ3, an evolutionary ancestor that links nostopeptolide and minutumamide biosynthesis and sheds light on cluster mixing as a widespread strategy in the evolution of metabolic diversity.

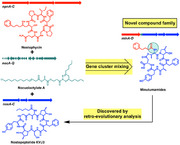

## Introduction

Natural products exhibit a striking structural diversity, which has been shaped by evolution over long periods of time. This diversity plays a central role in chemical biology because it is associated with a wide range of bioactivities. For example, natural products serve as signaling molecules or defense compounds for their producers while also being an important source of molecular tools and therapeutics for humans.^[^ ^1^, ^2^, ^3^ ^]^ The evolvability of biosynthetic pathways is the key to this diversity. Consequently, a comprehensive understanding of the principles and rules governing natural product evolution can facilitate the generation of artificial compound diversity.^[^ ^4^, ^5^, ^6^, ^7^, ^8^, ^9^, ^10^ ^]^

**Scheme 1:**
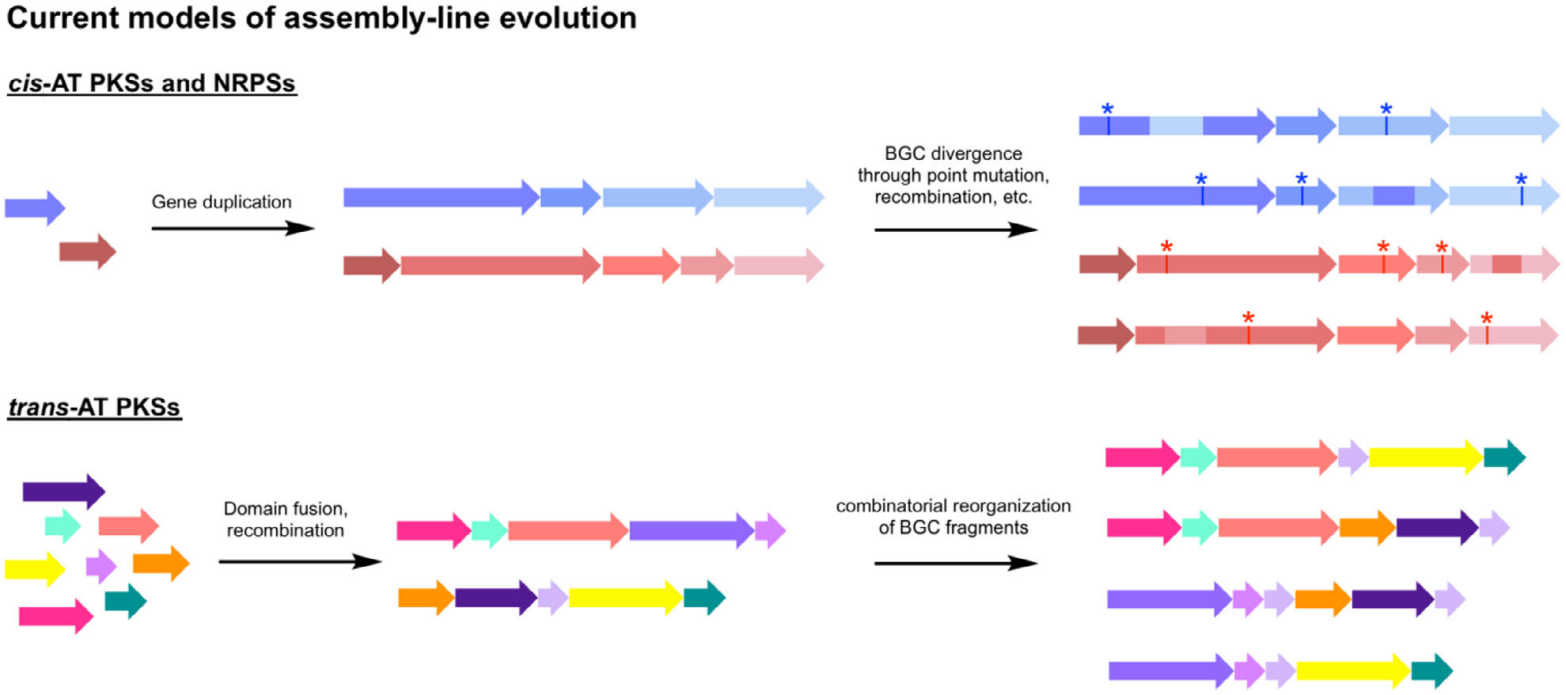
Current models of assembly‐line evolution. *cis*‐AT PKSs and NRPSs are believed to evolve primarily by gene duplications followed by BGC divergence^[^ ^12^, ^19^, ^21^, ^22^ ^]^ whereas *trans*‐AT PKSs are believed to have formed primarily through domain fusion and recombination leading to gene mosaics whose BGC fragments can be further recombined to form hybrid BGCs.^[^ ^12^, ^17^, ^18^, ^20^ ^]^

In general, biosynthetic gene clusters (BGCs) are regarded as rapidly evolving genomic entities that can undergo diversification through point mutation, gene duplication, and DNA recombination.^[^ ^11^, ^12^ ^]^ Over the past two decades, a number of seminal studies have significantly contributed to our understanding of the evolution of natural product biosynthesis.^[^ ^12^, ^13^, ^14^, ^15^, ^16^, ^17^, ^18^, ^19^, ^20^, ^21^, ^22^ ^]^ In the case of multimodular assembly lines, such as polyketide synthases (PKS) and nonribosomal peptide synthetases (NRPS), which particularly contribute to the diversity of bioactive natural products by catalyzing the biosynthesis of polyketides, nonribosomal peptides, and hybrids thereof,^[^ ^18^, ^23^ ^]^ the prevailing view is that they have evolved by two distinct mechanisms (Scheme 1):^[^ ^12^, ^17^, ^18^, ^19^, ^20^ ^]^ *trans*‐acyltransferase (*trans*‐AT) PKSs, which differ from *cis*‐AT PKSs in that they employ free‐standing ATs that act in *trans*,^[^ ^24^ ^]^ are believed to have diversified primarily through horizontal gene transfer and combinatorial reorganization of large gene fragments comprising multiple PKS modules, so‐called module blocks.^[^ ^12^, ^17^, ^18^, ^20^ ^]^ In contrast, an alternative hypothesis has been proposed for *cis*‐AT PKSs. This hypothesis suggests that prototypical multimodular PKSs may have originated from the successive duplication of a parent module, which subsequently evolved into distinct yet functionally related contemporary PKS families.^[^ ^19^, ^21^, ^22^ ^]^ Similarly, although NRPSs have been less extensively studied from an evolutionary perspective, it was anticipated that they would evolve through the duplication of modules and subsequent sequence divergence.^[^ ^12^ ^]^ Given the vast diversity of *cis*‐AT PKS and NRPS products, however, the question arises as to how this wealth of compounds could have been created predominantly by duplication of single modules.^[^ ^18^, ^25^ ^]^ Additionally, the current models fail to account for the evolution of the numerous hybrid *cis*‐AT PKS/NRPS pathways, which obviously requires the combination of fundamentally different types of modules.^[^ ^5^ ^]^

Herein we report the discovery of a novel family of hybrid polyketide‐peptide compounds, whose structures suggest mixing and matching of distinct cyanopeptide biosynthetic pathways. By employing retro‐evolutionary analysis and structure‐guided genome mining, we uncover substantial evidence for a number of hitherto unanticipated cluster‐mixing events in the evolutionary history of NRPS and PKS/NRPS BGCs in cyanobacteria that drive the generation of structural diversity. This unexpected mosaic‐like evolution indicates a much more pronounced evolutionary plasticity of modular megasynthases across class boundaries than is currently anticipated, offering inspiration for pathway engineering that capitalizes on natural recombination schemes.

## Results and Discussion

### Discovery of Minutumamides

**Figure 1:**
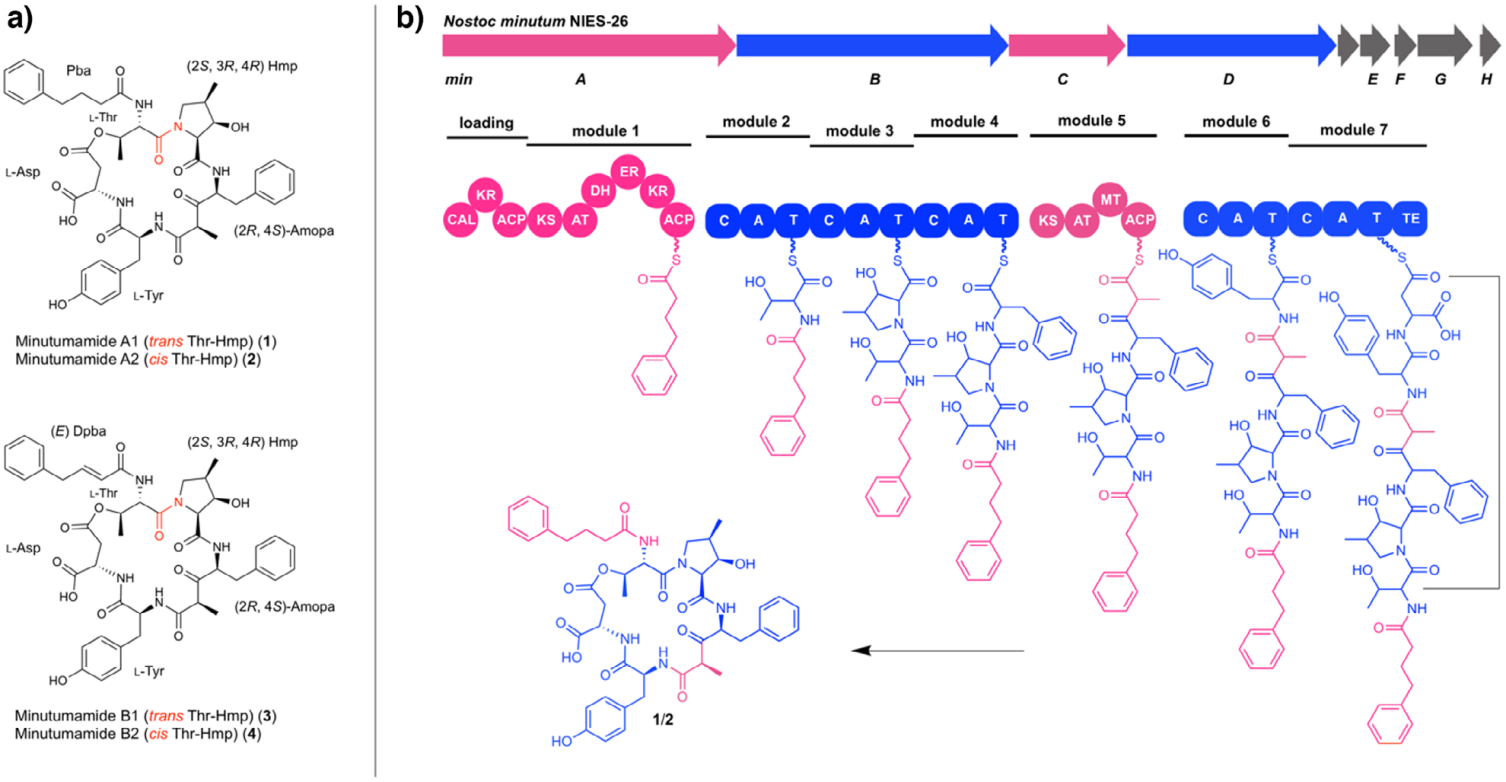
Discovery of a novel hybrid peptide‐polyketide compound family. a) Structures of minutumamides A1 (**1**) and A2 (**2**) and B1 (**3**) and B2 (**4**). **1** and **2** differ from **3** and **4** by the degree of saturation of the side chain (Pba vs. (*E*) Dpba). The *cis*‐*trans* amide bond between Thr and Hmp is colored in red. b) Putative minutumamide BGC (*min*) of *Nostoc minutum* NIES‐26 and the proposed biosynthesis of **1** and **2**. Substructures that are PKS‐derived are colored in pink, whereas substructures that are NRPS‐derived are colored in blue. Amopa, 4‐amino‐3‐oxo‐2‐methyl‐5‐phenylvaleric acid; Hmp, 4‐methyl‐3‐hydroxyproline; Pba, 4‐phenylbutyric acid; Dpba, 4‐phenyl‐2‐butenoic acid; CAL, co‐enzyme A ligase; KR, ketoreductase; ACP, acyl carrier protein; KS, ketosynthase; AT, acyltransferase; DH, dehydratase; ER, enoyl reductase; C, condensation domain; A, adenylation domain; T, thiolation domain; MT, methyltransferase; and TE, thioesterase.

In the search for novel microbial natural products, we screened a panel of *Nostoc* spp., given that cyanobacteria represent a rich yet underexplored source of pharmacologically interesting and structurally intricate compounds. The genus *Nostoc* is notable for its abundance of cryptic BGCs encoding modular megasynthases, rendering it an optimal subject for genome mining campaigns.^[^ ^26^, ^27^, ^28^, ^29^ ^]^ Following analysis by HR‐MS, two promising candidates with a predicted molecular formula of C_45_H_53_N_5_O_12_ were identified and successfully isolated from extracts of *Nostoc minutum* NIES‐26. Subsequently, we employed comprehensive 1D and 2D NMR analysis in conjunction with chemical derivatization and degradation experiments to elucidate the structure of the compounds (Figures S1‒S45). The analysis revealed that the compounds are novel cyclic pentadepsipeptides featuring an unusual 4‐phenylbutyric acid (Pba) side chain (Figure 1). Interestingly, both compounds, which we named minutumamide A1 (**1**) and A2 (**2**), exhibit the same absolute configuration, indicating that the peptides are conformational isomers. Finally, temperature‐dependent ^1^H NMR analysis of amide protons^[^ ^30^ ^]^ strongly suggests the presence of a *cis*‐*trans* amide bond between Thr and 4‐methyl‐3‐hydroxyproline (Hmp), resulting in the formation of two different rotamers (Figures S46‒S50 and Table S5)—an assumption that is supported by the observed long‐range NOEs (Figure S51; for more details on the structure elucidation of these compounds, see Supporting Information).

A high proportion of *cis* proline is a characteristic of cyclic peptides, in which the conformational restrictions imposed by the ring closure enhance the occurrence of the *cis* state. A systematic search of *cis* prolines in cyclic and acyclic peptides from the Cambridge Structural Database by Vitagliano et al. revealed that the proportion of *cis* Pro is 57.4% and 5.6% in cyclic and acyclic peptides, respectively.^[^ ^31^ ^]^ However, in contrast to the high proportion of *cis* Pro in cyclic peptides, reports on mixtures of natural proline rotamers such as *cis*,*cis*‐ and *trans*,*trans*‐ceratospongamide^[^ ^32^ ^]^ are exceptionally rare. In addition, Pba is a very rare building block in PKS/NRPS biosynthesis, which, as far as we know, is only present in prexenocoumacin E, a prodrug of the antibiotic xenocoumacin from *Xenorhabdus nematophila*.^[^ ^33^ ^]^

From a biosynthetic perspective, the structures of compounds **1** and **2** suggest a mixed PKS‐NRPS origin. To support this assumption, we searched for the putative minutumamide biosynthesis gene cluster (BGC) in the genome of *N. minutum* NIES‐26. Given that each of the highly versatile assembly lines in PK and NRP biosynthesis typically comprises specialized domains and modules for chain initiation, elongation, and termination, and that the number and type of modules are responsible for chain length, choice of building blocks, and the type of processing in a unidirectional assembly, it is possible to predict a BGC from the structure of a compound and vice versa.^[^ ^23^, ^34^ ^]^ This procedure resulted in the identification of a potential BGC (*min*) coding for a PKS/NRPS hybrid system that is perfectly collinear with the proposed biosynthesis of minutumamide A1 and A2 (Figure 1b), thereby providing further support for the structural assignment. Notably, we could isolate a set of highly similar congeners from *Nostoc linckia* NIES‐25. These compounds, which we named minutumamide B1 (**3**) and B2 (**4**), comprise 4‐phenylbutanoic acid (Dpba) in place of Pba (Figures 1 and S1‒S51; Supporting Information). Consequently, **3** and **4** differ from **1** and **2** solely in the nature of the side chain.

### Discovery of Nostopeptolide KVJ3 by Structure‐Guided Genome Mining

**Figure 2:**
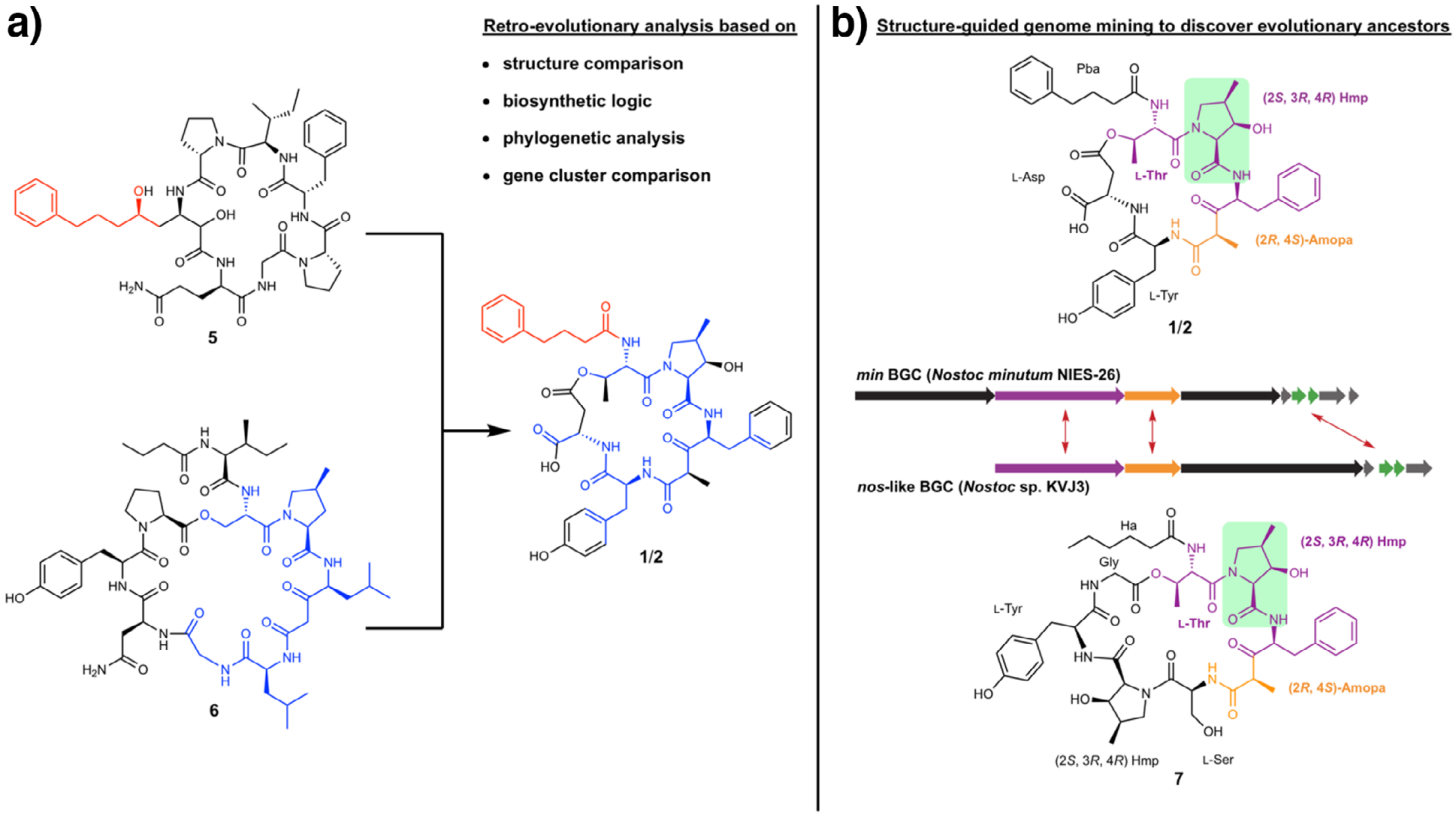
Structural similarity of cyanopeptides from the genus *Nostoc*. a) Minutumamides A1/A2 (**1**/**2**) distantly resemble streamlined, ring‐contracted versions of the cyanopeptide nostopeptolide A1 (substructure marked in blue) that got fused with the PKS‐derived side chain of another cyanopeptide, nostophycin (substructure marked in red). Retro‐evolutionary analysis suggests a shared evolutionary history of minutumamides with nostophycin (**6**) and nostopeptolide A1 (**5**). b) Structure of nostopeptolide KVJ3 (**7**), which was discovered by structure‐guided genome mining with *min* biosynthesis genes as query. Highly homologous NRPS and PKS genes, as well as genes associated with the biosynthesis of Hmp^[^ ^36^ ^]^ are colored in purple, orange, and green. Amopa, 4‐amino‐3‐oxo‐2‐methyl‐5‐phenylvaleric acid; Ha, hexanoic acid; Hmp, 4‐methyl‐3‐hydroxyproline; Pba, 4‐phenylbutyric acid; and Dpba, 4‐phenyl‐2‐butenoic acid.

Structurally, the minutumamides distantly resemble streamlined, ring‐contracted versions of the cyanopeptide nostopeptolide A1 (**5**)^[^ ^35^ ^]^ fused to the PKS‐derived side chain of another cyanopeptide, nostophycin (**6**) (Figure 2a).^[^ ^37^ ^]^ To test the possibility of a common biosynthetic origin, we compared the *min* BGC with the nostophycin (*npn*)^[^ ^38^ ^]^ and nostopeptolide (*nos*) BGCs^[^ ^39^ ^]^ and analyzed the encoded megasynthases. By phylogenetic analysis, we indeed confirmed a close relationship between acyltransferase (AT), ketosynthase (KS), and condensation (C) domains that are involved in minutumamide biosynthesis with domains from nostopeptolide and nostophycin biosynthetic pathways (Figures S52–S54). However, structural differences between minutumamides and nostopeptolides, as well as the differences in the corresponding biosynthetic genes, were too pronounced to allow for a conclusive reconstruction of the evolutionary history.

Therefore, we aimed at identifying descendants of potential evolutionary intermediates by structure‐guided genome mining. This approach could help unravel the complex evolutionary history of minutumamides. By performing bioinformatic analysis using minutumamide biosynthetic genes as a handle, we identified a candidate BGC in the genome of *Nostoc* sp. KVJ3, which was recently sequenced by us.^[^ ^40^ ^]^ Based on detailed in silico analysis, this BGC tentatively encodes the biosynthetic machinery for a ring‐contracted 7‐membered nostopeptolide congener that closely resembles the macrolactone ring of minutumamides but lacks their characteristic aromatic side chain (Figure S55). Therefore, this compound may serve as a structural link between nostopeptolide A and minutumamides. To isolate the predicted compound, we cultured *Nostoc* sp. KVJ3 to high cell density. By high‐resolution LC‐MS monitoring, we detected a potential candidate (**7**) with the deduced molecular formula of C_48_H_65_N_7_O_14_. We isolated pure **7** by reversed‐phase column chromatography followed by reversed‐phase HPLC and elucidated its structure by a combination of ^1^H NMR, ^13^C NMR, ^1^H‐^1^H COSY, HSQC, HMBC, and Marfey's analysis. The results confirmed the discovery of a novel 7‐membered nostopeptolide congener, which we named nostopeptolide KVJ3 (Figures 2b and S56‒S63; for details on structure elucidation, see Supporting Information).

### Cluster‐Mixing Event in the Evolution of Minutumamides

**Figure 3:**
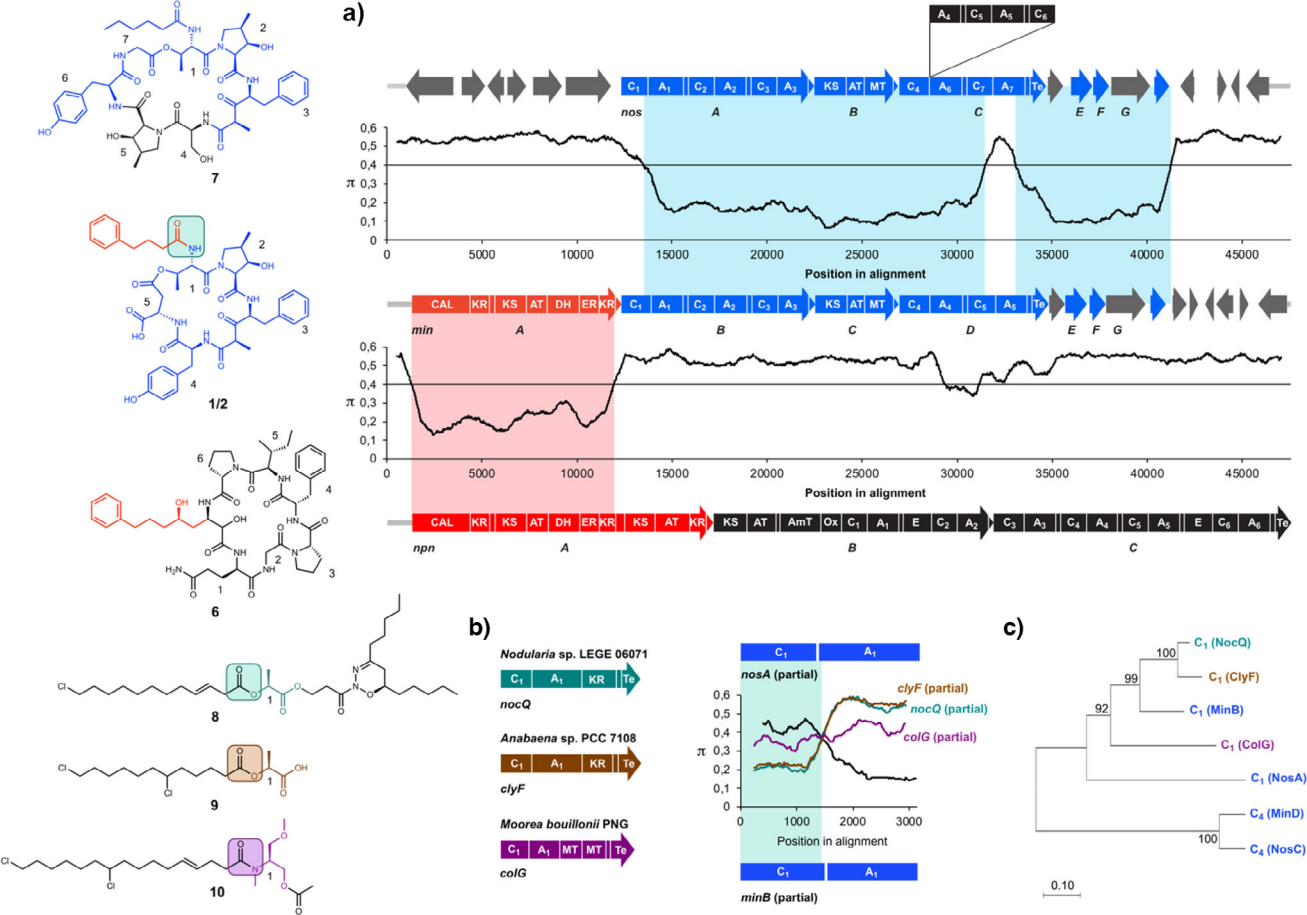
Comparison of nostopeptolide KVJ3 (**7**), minutumamide A1/A2 (**1**/**2**), nostocphycin (**5**) structures, and biosynthetic genes. a) Comparison of the BGCs of nostopeptolide KVJ3, minutumamide A1/A2, and nostophycin by sliding window analysis reveals pronounced sequence homology between *minA* and *npnA* and *minB‐G* with *nosA‐G*, thereby indicating a cluster mixing event in the evolution of minutumamides. π‐Values (average number of nucleotide differences per site between two sequences) were computed using the sliding window mode in DnaSP. b) Sliding window analysis reveals pronounced divergence in the first C domain of *nosA* compared to *minB*, which can be complemented by C domains encoded in the BGCs of nocuolactylate A (*noc*) or chlorosphaerolactylate A (*cly*) and, to a lesser extent, by a C domain encoded in the BGC of columbamide A (*col*). c) Phylogenetic analysis confirms the close relationship of C_1__MinB with C domains from nocuolactylate A (**8**) (C_1__NocQ) and chlorosphaerolactylate A (**9**) biosynthesis (C_1__ClyF) as well as more distant homology to the C domain of ColG involved in the biosynthesis of columbamide A (**10**). CAL, co‐enzyme A ligase; KR, ketoreductase; KS, ketosynthase; AT, acyltransferase; DH, dehydratase; ER, enoyl reductase; C, condensation domain; A, adenylation domain; T, thiolation domain; MT, methyltransferase; AmT, aminotransferase; Ox, oxygenase; E, epimerase; TE, thioesterase; acyl carrier proteins and thiolation domains are not labeled due to space limitations.

To test our hypothesis that nostopeptolide KVJ3 is a descendant of an evolutionary intermediate in the evolution of minutumamides, we compared its BGC with the BGC for minutumamides A1 and A2. Sliding window analysis shows high sequence homology for the majority of genes, indicating a shared evolutionary origin (Figure 3a). However, there is a notable absence of a sequence stretch in *minD* encoding A_4_T_4_C_5_A_5_T_5_C_6_ when compared to *nosC*. Additionally, there is a considerably high divergence in parts of *C_7_A_7_* in *nosC* and *minD*, which results in ring contraction and a different amino acid in position 5 of minutumamides when compared to nostopeptolide KVJ3 (position 7). This demonstrates clear diversification in the course of evolution. In addition, the two clusters are readily distinguishable due to the presence of the PKS gene *minA* within the BGC of minutumamides. To test our hypothesis of a shared evolutionary history of *minA* and nostophycin biosynthesis genes, we compared the BGC of minutumamide A1 and A2 with the BGC of nostophycin. A sliding window analysis indicates a high degree of sequence homology between *minA* and the PKS gene *npnA*, providing substantial evidence in support of the hypothesis that a cluster‐mixing event has occurred, whereby a nostophycin BGC has been combined with a remodeled and streamlined nostopeptolide BGC (Figure 3a).

### Fine‐Tuning of Recombined Pathways by Module Adjustment

Notably, the first C domain encoded by *minB* shows pronounced divergence compared to the starter C domain encoded by *nosA* (Figure 3a), indicating module adjustment after the assumed cluster‐mixing event. Instead, BLAST analysis revealed high similarity to C domains of NocQ involved in the biosynthesis of nocuolactylate A (**8**)^[^ ^41^ ^]^ and ClyF involved in the biosynthesis of chlorosphaerolactylate A (**9**)^[^ ^42^ ^]^ as well as more distant homology to the C domain of ColG involved in the biosynthesis of columbamide A (**10**) (Figure S64).^[^ ^43^ ^]^ These C domains have been proposed to cooperate with downstream PKSs to fuse either fatty acids with hydroxy acids, as in the case of NocQ and ClyF, or a reduced polyketide with an amino acid, as in the case of ColG. While ColG, apart from a rare MT domain tandem follows standard NRPS architecture NocQ and ClyF have typical depsipeptide synthetase architectures.^[^ ^42^ ^]^ Sliding window analysis confirmed the close relationship on the genetic level in particular for the two C domains encoded by *nocQ* and *clyF* (Figure 3b). The same trend can be seen by phylogenetic analysis that demonstrates close relationship of MinB_C_1_ with NocQ_C_1_ from nocuolactylate^[^ ^41^ ^]^ and ClyF_C_1_ from chlorosphaerolactylate biosynthesis,^[^ ^42^ ^]^ more distant relationship with ColG_C_1_^[^ ^43^ ^]^ and only remote relationship with NosA_C_1_.^[^ ^39^ ^]^ From a functional point of view, it seems surprising that MinB_C_1_ is more closely related to C domains that condense fatty acids with hydroxy acids than to a related C domain that condense a PKS product with an amino acid. However, *Nodularia* and *Anabaena* spp. are more closely related to *Nostoc* spp. than the genus *Moorea*, making the transfer of genetic material by horizontal gene transfer with subsequent gene mixing much more likely.^[^ ^44^ ^]^ Remarkably, there is no close relationship of MinB_C_1_ with NosC_C_4_ or MinD_C_4_, which form an outgroup (Figure 3c), although both C domains also work downstream of PKS modules.

However, NocQ_C_1_ seems not to be the only remnant from the *noc* BGC, since C‐ and N‐terminal docking domains (^C^dd; ^N^dd) can be found in MinA and MinB, which are closely related to the C‐ and N‐terminal dds of NocP and NocQ and therefore seem to be co‐adjusted to yield a functional hybrid pathway. As observed for the C domains, the relationship of dds, which are related to the prototype dds, TubB_^C^dd, and TubC_^N^dd,^[^ ^45^ ^]^ is far more pronounced to NocP_^C^dd and NocQ_^N^dd than to the dds of NosB and NosD or MinC and MinD (Figure S65).

From an engineering perspective, it is intriguing that two unrelated multimodular PKS and NRPS assembly lines can be combined simply by C domain and docking domain adjustment. Thus, if applicable to engineering, this concept would have great potential for the design of structural hybrids.

Apparently, cluster mixing and module adjustment led to a novel pathway and, consequently, a novel scaffold. This scaffold appears to have been further diversified during evolution, as minutumamides A1 and A2 differ from minutumamides B1 and B2 in the presence of a double bond in the PKS‐derived side chain. In agreement with this structural difference, MinA from *N. linckia* NIES25 lacks an ER domain that is present in MinA from *N. minutum* NIES26, indicating domain deletion as a strategy to explore chemical diversity (Figure S66).

### Shared Evolutionary Origin of Nostopeptolide A1 and Nostopeptolide KVJ3

**Figure 4:**
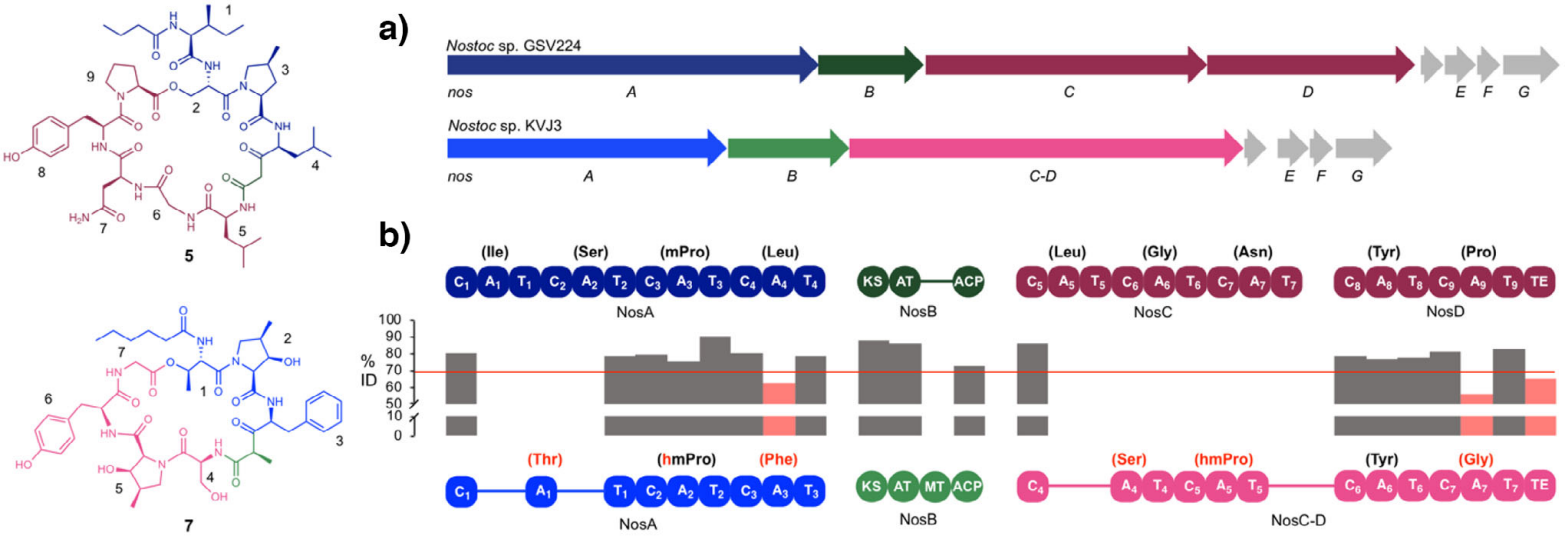
Comparison of the nostopeptolide A1 (**5**) and nostopeptolide KVJ3 (**7**) biosynthetic machinery on the genetic and enzymatic level. a) Organization of *nos* BGCs in *Nostoc* sp. GSV224 and *Nostoc* sp. KVJ3. b) Pairwise comparison of NRPS/PKS modules on the protein sequence level demonstrates high degree of similarity between domains in the majority of cases (>70%), thereby underlining an evolutionary relationship. However, various domains and even complete modules were absent in either of the clusters or differed substantially (indicated in red), thereby indicating evolutionary divergence. KS, ketosynthase; AT, acyltransferase; ACP, acyl carrier protein; KR, ketoreductase; MT, methyltransferase; C, condensation domain; A, adenylation domain; T, thiolation domain; and TE, thioesterase.

The overall architecture of nostopeptolide KVJ3 and nostopeptolide A1 exhibits notable similarities, including the position of the macrolactone bond relative to the polyketide substructure and the presence of a fatty acid moiety in the side chain. Although these similarities indicate a shared evolutionary origin, there are nevertheless substantial differences between the two compounds. For example, there are differences in the ring topologies (7‐membered vs. 8‐membered), nostopeptolide KVJ3 is missing an amino acid in the side chain, and there is significant variation in the amino acid composition (Figure S67). Accordingly, the evolution of a related biosynthetic pathway would require extensive remodeling of nostopeptolide biosynthesis genes. To investigate the relationship between the two compounds, we compared the BGC of nostopeptolide A1 and the putative BGC of nostopeptolide KVJ3 (Figure 4a). In line with the assumption of a shared evolutionary history, both gene clusters show a highly similar organization, including genes *nosE* and *nosF*, which encode the enzymes for the biosynthesis of methylproline, as well as *nosG* encoding an ABC transporter presumably involved in nostopeptolide export.^[^ ^36^ ^]^ The only obvious difference is that the genes *nosC* and *nosD* are combined into a single ORF. Moreover, pairwise comparison of the encoded megasynthases demonstrates high degree of similarity between domains in the majority of cases (>70%), thereby underlining an evolutionary relationship (Figure 4b). However, various sequences encoding domains and even complete modules were absent from either of the clusters or differed substantially from them, indicating evolutionary divergence. These results suggest that an ancestral nostopeptolide BGC diverged into the pathways of nostopeptolide KVJ3 and nostopeptolide A1 by several module and domain deletions or expansions as well as (sub)domain swaps in the course of evolution (for a detailed analysis and an evolutionary model, see Supporting Information and Figures S68 and S69). Notably, two otherwise conserved modules differ significantly in the sequences of their A domains. This divergence results in the incorporation of different amino acids into the respective nostopeptolide (Leu and Pro in nostopeptolide A1 vs. Phe and Gly in nostopeptolide KVJ3, Figure 4b). This pattern is in accordance with recent findings indicating that the A core domain (A_core_) is a recombination hotspot whose diversification correlates with the diversification of NRPS compound families, whereas the C domain, the flexible A subdomain, and the T domain remain largely unaffected.^[^ ^46^, ^47^ ^]^

These results highlight the remarkable evolutionary plasticity of NRPS‐PKS assembly lines, in which gene cluster expansion and reduction provide shortcuts in the generation of structural diversity via the gain and loss of modules and domains. While the evolution of gene clusters is often associated with gene duplication, neofunctionalization, and the acquisition of genes via horizontal gene transfer, leading from simple systems to more complex ones,^[^ ^11^ ^]^ our results indicate that the loss of modules and domains is also a major driver of diversification. Consistent with this, gene deletions leading to reduced gene clusters and consequently simplified compound structures have also been proposed for other pathways, such as the evolution of aureothin from neoaureothin^[^ ^6^ ^]^ or nodularin from microcystin.^[^ ^48^ ^]^

From an engineering perspective, also this concept, which might be underappreciated in the evolution of structural diversity, holds great potential for engineering novel structures by deleting domains or modules. Notably, the putative module deletions/swaps in the evolution of nostopeptolide KVJ3 and minutumamides do not occur within the traditional boundaries of NRPS modules (C–A–T). Instead, they follow the alternative module architecture A–T–C, which appears to be strongly influenced by C domain (and docking domain) preservation or neofunctionalization at PKS/NRPS borders. Interestingly, alternative module boundaries have already been employed for the de novo design and engineering of NRPSs.^[^ ^49^, ^50^ ^]^

### Genetic Hub for the Evolution of Cyanopeptide Diversity

**Figure 5:**
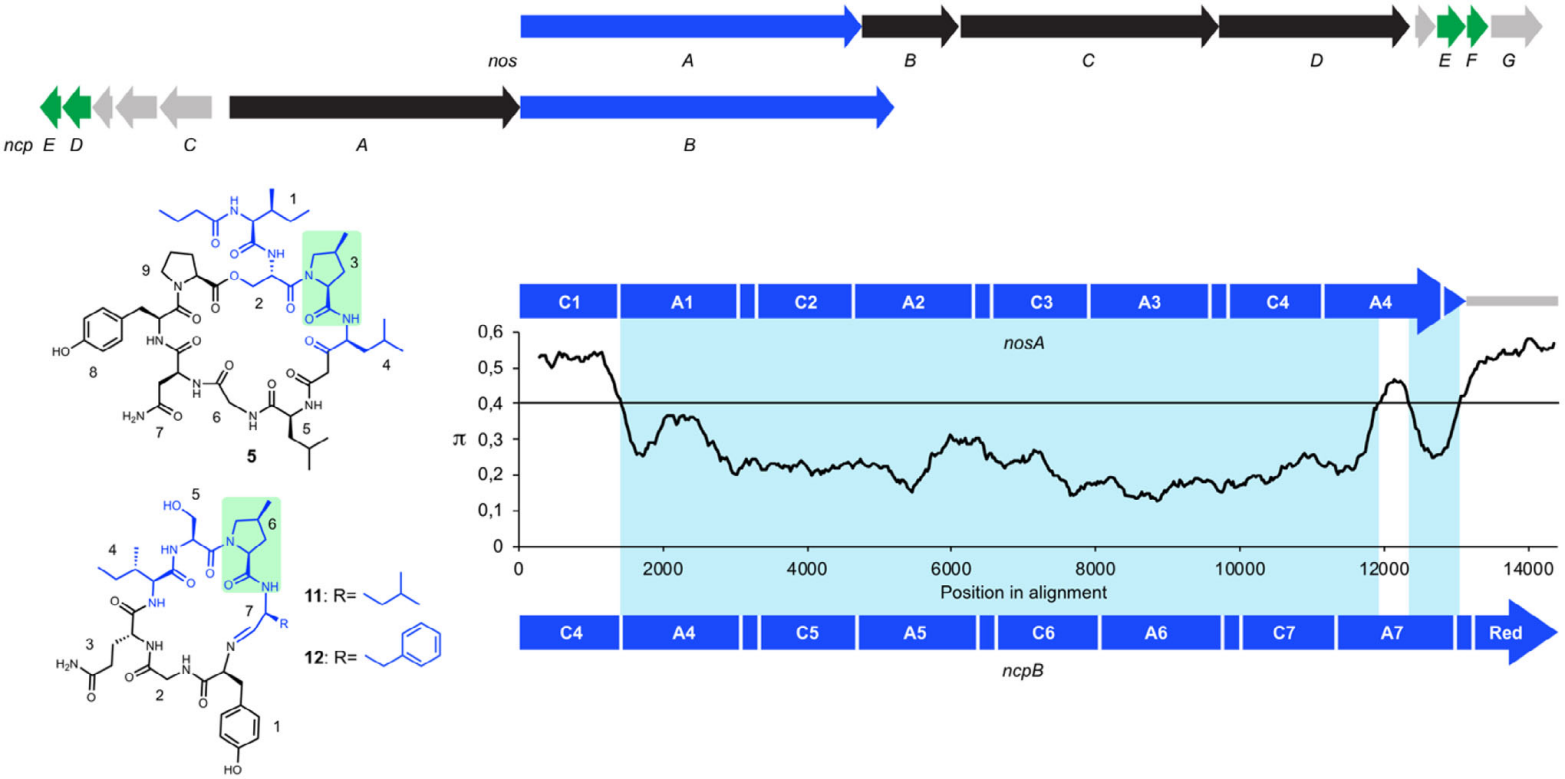
Comparison of nostopeptolide A1 (**5**) and nostocyclopeptide A1 (**11**) and A2 (**12**) structures and biosynthesis genes. The tetrapeptide unit Ile‐Ser‐mPro‐Leu (blue) is shared by both compounds. Genes encoding enzymes for the biosynthesis of 4‐methylproline are marked in green. Comparison of *nosA* and *ncpB* by sliding window analysis to compute the average number of nucleotide differences per site (π values) indicates a common evolutionary origin of both NRPS genes due to their pronounced sequence homology. C, condensation domain; A, adenylation domain; Red, reductase; thiolation domains are not labeled due to space limitations.

The minutumamides seem to be a prime example of how mixing and matching of biosynthesis genes together with the simultaneous adjustment of individual modules can create a new scaffold, which later is diversified by module swapping and/or deletions. However, this phenomenon might be more widespread, as indicated by the structure of nostocyclopeptide A1 (**11**)—another nonribosomal peptide produced by a *Nostoc* strain.^[^ ^51^ ^]^ This compound shares the tetrapeptide unit Ile‐Ser‐mPro‐Leu with nostopeptolide A1 (Figure 5). In addition, the domain architecture of the tetramodular enzyme NcpB, which is responsible for the incorporation of the corresponding four amino acids at positions 4–7 of nostocyclopeptides, is similar to that of NosA. To investigate a putative relationship, *ncpB* ^[^ ^38^ ^]^ and *nosA* ^[^ ^39^ ^]^ were aligned and compared by sliding window analysis. Notably, both genes show high homology over long stretches, thereby indeed indicating a common evolutionary origin (Figure 5).

However, similar to what has been observed for *minB* and *nosA*, the gene segment encoding the first C domain is highly divergent in both genes. This deviation is in accordance with different biosynthetic roles: whereas the first condensation domain of NosA links a fatty acid starter to the PCP‐bound leucyl moiety, the first condensation domain of NcpB connects the leucyl species to the PCP‐bound tripeptide synthesized by NcpA.^[^ ^52^ ^]^ Another major difference is that *ncpB* encodes a terminal reductase domain (Red). This domain serves as termination domain that catalyzes the reductive release of a linear aldehyde intermediate, which reacts further to the macrocyclic imine.^[^ ^53^ ^]^ These two adjustments reflect the transformation of a multimodular enzyme that initiates the biosynthesis of a cyanopeptin to a homolog that terminates it. Finally, both genes show divergence in a subdomain stretch of the terminal A domain (Figure 5). These results are in line with the altered substrate specificity of NcpB_A7, which also can activate phenylalanine besides its ability to activate leucine, leading to biosynthesis of nostocyclopeptide A2 (**12**). The close homology of *nosA*‐like genes in biosynthesis gene clusters of compounds as diverse as nostopeptolides, minutunamides, and nostocyclopeptides is highly remarkable and suggests that the ancestor of *nosA*, *minB*, and *ncpB* is a genetic hub for the evolution of cyanobacterial NRP diversity.

### Gene Cluster Mosaics Beyond *trans*‐AT PKS Genes

**Figure 6:**
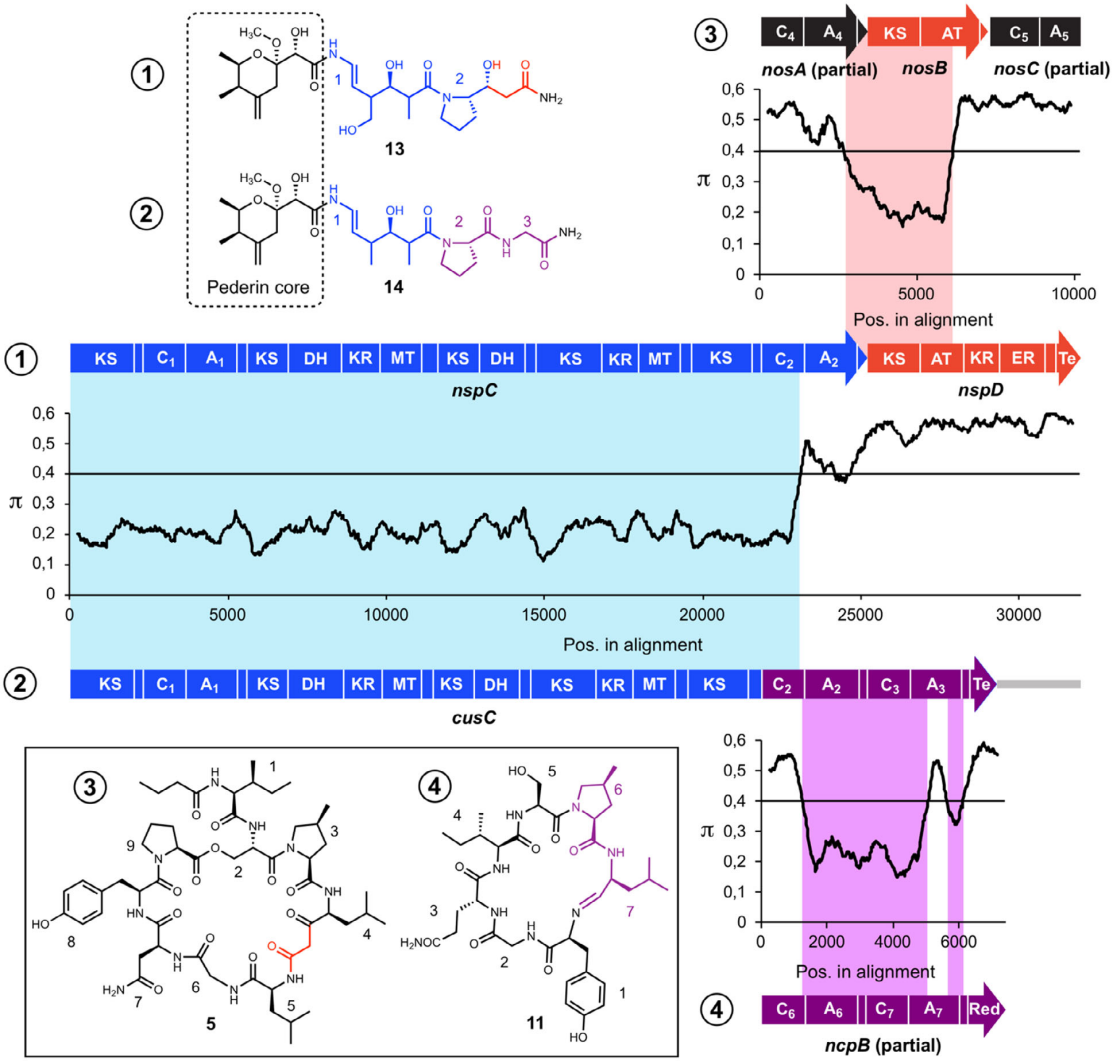
Comparison of the structures of nosperin (**13**) and cusperin A (**14**) and sliding window analysis of nosperin (*nsp*) and cusperin (*cus*) biosynthesis genes. The genes *nspC* and *nspD* in part show strong divergence on the genetic level that could be complemented by gene segments of the nostopeptolide (**5**) BGC (*nos*) or the nostocyclopeptide (**11**) BGC (*ncp*), thereby indicating gene cluster mixing. π‐Values (average number of nucleotide differences per site between two sequences) were computed using the sliding window mode in DnaSP. KR, ketoreductase; KS, ketosynthase; AT, acyltransferase; DH, dehydratase; ER, enoyl reductase; C, condensation domain; A, adenylation domain; MT, methyltransferase; TE, thioesterase; acyl carrier proteins and thiolation domains are not labeled due to space limitations.

Although gene fragments encoding NosA‐like module blocks seem widespread in *Nostoc* BGCs, they may not be the only genetic parts related to nostopeptide biosynthesis genes that contribute to BGC mosaics. Remarkably, two domains encoded by *nspD* in the nosperin BGC from *Nostoc* sp. “*Peltigera membranacea* cyanobiont” show high homology to a domain pair encoded by *nosB*.^[^ ^54^ ^]^ Nosperin (**13**) belongs to the pederin family of compounds. These often highly potent cytotoxins are predominantly produced by bacterial symbionts.^[^ ^24^ ^]^ Nosperin and the recently identified cusperin A (**14**) and B (**15**) represent the only cyanobacterial congeners of this compound family.^[^ ^54^, ^55^ ^]^ Both types of compounds are structurally highly similar but differ by the presence of a terminal reduced biketide substructure in nosperin, which is replaced by a glycine residue in cusperins (Figure 6).^[^ ^55^ ^]^ Consequently, the corresponding BGCs differ in the presence of a PKS gene in the nosperin BGC, which is accompanied by the absence of a terminal NRPS module.^[^ ^54^, ^55^ ^]^ Expectedly, sliding window analysis confirms a pronounced drop in sequence similarity in the terminal part of the BGCs, indicating mixing of different biosynthesis genes. Comparison of the PKS gene *nspD* with *nosB* shows pronounced homology of the sequences encoding the KS and AT domains, indicating a shared biogenetic origin, which has already been suggested by Kampa et al.^[^ ^54^ ^]^ However, additional PKS modules encoded by *nspD* suggest a more complex evolutionary history involving further domain mixing. The assumption that shuffling of module parts contributed to the evolution of the nosperin BGC is further supported by the odd module architecture of NspD (KS‐AT‐KR‐ER‐ACP‐TE), which has an orphan ER domain and is strikingly different from the canonical sequence KS‐AT‐DH‐ER‐KR‐ACP‐TE.^[^ ^54^ ^]^ In addition, the gene fragment *cusC_A_2_T_2_* is highly divergent compared to its counterpart from *nspC*. This is surprising considering that the corresponding modules both catalyze the activation and incorporation of proline into the growing chain. Remarkably, BLAST analysis revealed high similarity of these domains with NcpB from nostocyclopeptide biosynthesis,^[^ ^56^ ^]^ once again underlining the importance of *nosA* homologs for BGC expansion and the emergence of chemical novelty. Sliding window analysis of *cusC* and *ncpB* confirms the homology on the genetic level, indicating a cluster‐mixing event of a nosperin‐like BGC and a nostocyclopeptide‐like BGC in the evolution of cusperins (Figure 6). Divergence in the terminal A_core_ domain as well as the termination domain (thioesterase vs. reductive domain), which is in line with the corresponding structures of cusperins and nostocyclopeptides, points to further module adjustment in the course of evolution.

The evolution of pederin‐type compounds is a textbook example of how combinatorial reorganization of *trans*‐AT PKS gene fragments creates a variety of divergent BGCs.^[^ ^17^, ^24^ ^]^ However, the examples of nosperin and cusperin biosynthesis exemplify that this concept is by no means limited to *trans*‐AT PKS but also includes mixing and matching of NRPS and *cis*‐AT PKS genes.

### Novel Unified Model of Assembly‐Line Evolution

**Scheme 2:**
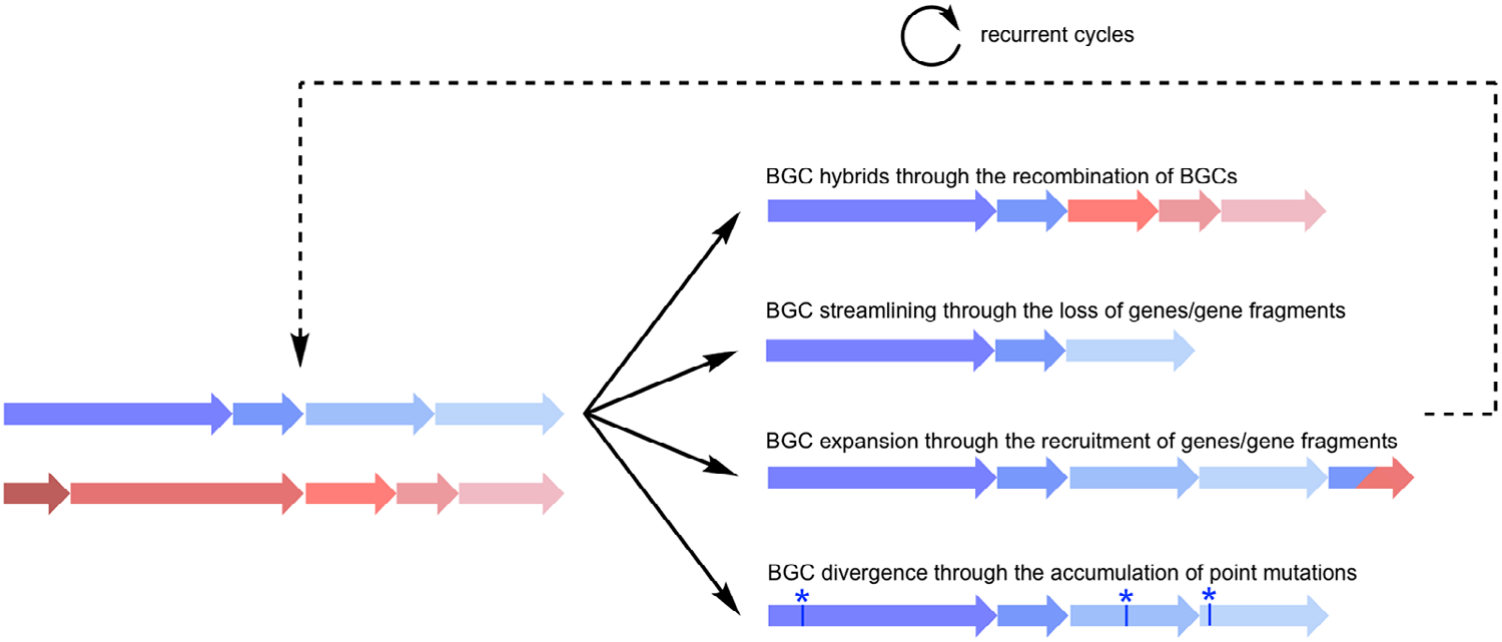
Unified model of assembly‐line evolution. Diversification of assembly line BGCs, such as for *cis*‐AT PKS, *trans*‐AT PKS, or NRPS, could occur via recombination to create BGC hybrids, BGC streamlining through the loss of genes or gene fragments, BGC expansion through the recruitment of genes or gene fragments, and BGC divergence through the accumulation of mutations.

The enormous structural diversity in the natural product cosmos is reflected in more than 340 000 described compounds.^[^ ^57^ ^]^ In parallel with the discovery of more and more new compound families over the past decades, our understanding of the biosynthetic logic and the enzymatic machinery of natural product biosynthesis has grown steadily.^[^ ^58^, ^59^, ^60^, ^61^, ^62^, ^63^ ^]^ In contrast, our understanding of the evolutionary principles that have led to this impressive diversity and complexity is clearly lagging behind.^[^ ^64^, ^65^ ^]^ A key principle that nature uses to constantly develop chemical innovations with limited resources is the combination of pre‐validated substructures and natural product fragments to form new hybrids—a concept that is also becoming increasingly popular in synthesis to create pharmacologically active substances, so‐called pseudo‐natural products, following nature's example.^[^ ^66^, ^67^, ^68^ ^]^ Due to their modular design, megasynthases are theoretically predestined to be combined in all conceivable ways. However, although the different thio‐template assembly lines share many biosynthetic principles, the recombination of extended module series in the evolution of natural hybrids is considered to be a predominant feature of *trans*‐AT PKSs.^[^ ^12^, ^17^, ^18^, ^20^ ^]^ In a recent study, we showed that recombination events in gene segments that encode A domains are a key driver for the diversification of bacterial NRPS compound families.^[^ ^47^ ^]^ While these results highlight a key evolutionary strategy to diversify a given scaffold, it is far from understood how NRPS biosynthetic pathways or NRPS‐PKS hybrid biosynthetic pathways evolve to create novel compound architectures. Our results provide ample evidence for several unexpected cluster‐mixing events in the course of evolution that led to the emergence of novel nonribosomal peptide and hybrid peptide‐polyketide BGCs in cyanobacteria. The resulting *cis*‐AT PKS‐NRPS mosaic in the biosynthesis of minutumamides, together with additional examples from nostocyclopeptide, nosperin, and cusperin biosynthesis revealed in this study, are unprecedented cases of recombined pathways, complementing the known examples from *trans*‐AT PKSs^[^ ^17^, ^20^ ^]^ or siderophore biosynthesis.^[^ ^69^ ^]^ This diversity urgently calls for a more general model of recombination schemes in the evolution of modular megasynthases that is not restricted to a particular class of enzymes or compounds (Scheme 2).

## Conclusion

In summary, we discovered a novel family of hybrid polyketide‐peptide compounds and showed that genetic elements of at least three different pathways have been rearranged in the evolution of their biosynthesis. Structure‐guided genome mining identified an unusual ring‐contracted nostopeptolide congener, nostopeptolide KVJ3, linking nostopeptolide and minutumamide biosynthesis. Retro‐evolutionary analysis suggests that a *nos* pathway has been redesigned and streamlined through multiple module, domain, and subdomain exchanges and deletions during evolution, which was then mixed with a nostophycin‐like BGC and a BGC of cyanobacterial lactylates to give the minutumamide pathway. Tracing of *nos*‐like genetic elements in other megasynthase pathways reveals vivid mixing and matching of hybrid megasynthase clusters in cyanobacteria. The design principles revealed by the in‐depth dissection of natural combinatorial biosynthesis in this study may inspire pathway engineers in search of productive combinations that lead to “nonnatural” hybrid natural products. In recent years, a trend toward evolution‐inspired engineering for modular megasynthases has emerged, which is fueled by the increasing understanding of pathway evolution.^[^ ^4^, ^5^, ^6^, ^7^, ^8^, ^9^, ^10^, ^70^ ^]^ Thus, traceable recombination schemes in the evolution of natural product diversity are valuable resources for future engineering campaigns. The combination of structure comparison, retro‐evolutionary analysis, and genome mining appears to be a promising strategy to unravel the complex evolutionary history of megasynthases and to gain insights into productive recombination schemes. We envision that this approach will aid in elucidating many more gene cluster mosaics and hybrid natural products in the future.

## Supporting Information

The authors have cited additional references within the Supporting Information.^[^ ^71^, ^72^, ^73^, ^74^, ^75^, ^76^, ^77^, ^78^, ^79^, ^80^, ^81^, ^82^ ^]^

## Conflict of Interests

The authors declare no conflict of interest.

## Supporting information



Supporting Information

## Data Availability

The data that support the findings of this study are available from the corresponding author upon reasonable request.
