# Supplementary material for: Mixing and Matching of Hybrid Megasynthases is a Hub for the Evolution of Metabolic Diversity in Cyanobacteria
Source: Angew Chem Int Ed Engl. 2025 May 19;64(26):e202502461. doi: 10.1002/anie.202502461 (PMC12184294; doi:10.1002/anie.202502461)
Supplement: Supplementary file 1 — Supporting Information [file ANIE-64-e202502461-s001.pdf]

**Table of content**

1. Experimental Section…………………………………………………………………………...2
2. Supplemental Notes………………………………………………...……………………..9
3. Supplemental Tables……………………………………………………………………..13
4. Supplemental Figures...……………………………………………………………………19

## 1. Experimental Section

### General Analytical Procedures

NMR spectra were measured on Bruker Avance 500 and 600 MHz spectrometers with a cryo probe. Spectra were referenced to the residual solvent peak DMSO-$d_6$; $\delta_H$ = 2.49 ppm, $\delta_C$ = 39.5 ppm, $D_2O$; $\delta_H$ = 4.79, $CD_3OD$; $\delta_H$ = 3.30. Ultraviolet spectra were measured on a Hitachi 330 spectrophotometer. Optical rotations were measured on a JASCO DIP-1000 polarimeter. HPLC-HRMS measurements were carried out on an QExactive Orbitrap Mass Spectrometer (Thermo Fisher Scientific) with an electrospray ion source using Accucore C18 column (2.6 µm, 100 Å, 2.1 × 150 mm, Thermo Fisher Scientific) and an elution gradient, solvent A: $H_2O$ containing 0.1% formic acid, solvent B: acetonitrile, gradient: 5% to 98% B in 10 min, 98% B for 4 min, flow rate: 0.2 mL $min^{-1}$.

### Cultivation of cyanobacteria

*Nostoc minutum* NIES-26 and *N. linckia* NIES-25 were obtained from the Microbial Culture Collection at the National Institute for Environmental Studies (NIES Collection, Tsukuba, Japan) and cultured in 10 L glass bottles containing CB medium[1] with aeration (filtered air at a flow rate of 0.3 L $min^{-1}$) at 25 °C under an illumination of 250 µE $mm^{-2}$ $s^{-1}$ on a 12L:12D cycle, respectively. After 25 days, the algal cells were filtered by 95 µm nylon plankton nets (Swiss Silk Bolting Cloth Mfg Co., Ltd) and lyophilized. The obtained freeze-dried cells were kept in a freezer at –20 °C until extraction.
*Nostoc* sp. KVJ3 was cultivated to high cell density (HD) in 100 mL of $BG11_0$[2] medium under continuous shaking for 18-21 days by using a HD-cultivation platform (CellDEG GmbH). A pressure of 32 mbar of $CO_2$ was achieved through the addition of $KHCO_3$ and $K_2CO_3$ buffer in the lower chamber of the cultivation vessel. Cells were harvested by centrifugation at 7000 g for 7 minutes and washed twice with $BG11_0$ medium and kept frozen at –20 °C for further use.

### Isolation of minutumamide A1 (1) and A2 (2) from *N. minutum* NIES-26

The dried cells (231 g from 590 L culture) were suspended in 80% aq. methanol (3 L) and homogenized. The homogenized cells were centrifuged and the residues were repeatedly extracted with 80% aq. methanol (3 L × 2) and 100% methanol (3 L × 1). Obtained extracts were combined and concentrated under reduced pressure. The residues were partitioned with diethyl ether and water. The water phase was further partitioned with *n*-butanol and water. The *n*-butanol phase (dry weight 12.6 g) was concentrated under reduced pressure and subjected to reversed-phase flash column chromatography (120 × 110 mm, YMC-ODS AM, YMC Co., LTD., Kyoto) with eluents of 20, 30, 40, 50, 60% aq. methanol, 100% methanol, and dichloromethane. The 40% aq. methanol eluted fraction (dry weight 341.0 mg) was subjected to reversed-phase flash column chromatography (60 × 35 mm, YMC-ODS AM) with eluents of 20, 30, 50% aq. methanol, 100% acetonitrile containing 0.05% trifluoroacetic acid, and dichloromethane. The 50% aq. acetonitrile containing 0.05% trifluoroacetic acid fraction (dry weight 98.2 mg) was subjected to reversed-phase HPLC (Capcell Pak

C18 UG, 250 × 10 mm, Shiseido Co., LTD) with 38% aq. acetonitrile containing 0.05% trifluoroacetic acid at flow rate 2 mL min$^{-1}$ to yield minutumamide A1 (**1**, 38.0 mg) and A2 (**2**, 5.0 mg).

**Minutumamide A1 (1)**. White powder. $[\alpha]_D$ –90.0° (*c* = 0.31, methanol). UV (methanol) $\lambda_{max}$ 278 nm (*ε* 1,800). HRESIMS: $[M + H]^+$ = 856.3784 (calculated for $C_{45}H_{54}N_5O_{12}$, 856.3763).

**Minutumamide A2 (2)**. White powder. $[\alpha]_D$ +78.5° (*c* = 0.20, methanol). UV (methanol) $\lambda_{max}$ 278 nm (*ε* 1,800). HRESIMS: $[M + H]^+$ = 856.3777 (calculated for $C_{45}H_{54}N_5O_{12}$, 856.3763).

**Isolation of minutumamide B1 (3) and B2 (4) from *N. linckia* NIES-25.**

The dried cells (273 g from 550 L culture) were suspended in 80% aq. methanol (3 L) and homogenized. The homogenized cells were centrifuged and the residues were repeatedly extracted with 80% aq. methanol (3 L × 2) and 100% methanol (3 L × 1). Obtained extracts were combined and concentrated under reduced pressure. The residues were partitioned with diethyl ether and water. The water phase was further partitioned with *n*-butanol and water. The *n*-butanol phase (dry weight 9.8 g) was concentrated under the reduced pressure and subjected to reversed-phase flash column chromatography (150 × 110 mm, YMC-ODS AM) with eluents of 20, 30, 40, 60, 80% aq. methanol, 100% methanol, and dichloromethane. The 40% aq. methanol eluted fraction (dry weight 438.4 mg) was subjected to reversed-phase flash column chromatography (60 × 35 mm, YMC-ODS AM) with eluents of 20, 30, 50% aq. methanol, 100% acetonitrile containing 0.05% trifluoroacetic acid, and dichloromethane. The 50% aq. acetonitrile containing 0.05% trifluoroacetic acid fraction (dry weight 72.0 mg) was subjected to reversed-phase HPLC (Capcell Pak C18 UG, 250 × 10 mm) with 38% aq. acetonitrile containing 0.05% trifluoroacetic acid at flow rate 2 mL min$^{-1}$ to yield minutumamide B1 (**3**, 19.6 mg) and B2 (**4**,14.6 mg).

**Minutumamide B1 (3)**. White powder. $[\alpha]_D$ –56.7° (*c* = 0.39, MeOH). UV (methanol) $\lambda_{max}$ 277 nm (*ε* 1,400). HRESIMS: $[M + H]^+$ = 854.3600 (calculated for $C_{45}H_{52}N_5O_{12}$, 854.3607).

**Minutumamide B2 (4)**. White powder. $[\alpha]_D$ +67.4° (*c* = 0.36, MeOH). UV (methanol) $\lambda_{max}$ 277 nm (*ε* 1,300). HRESIMS: $[M + H]^+$ = 854.3600 (calculated for $C_{45}H_{52}N_5O_{12}$, 854.3607).

**Isolation of nostopeptolide KVJ3 (7) from *Nostoc* sp. kvj3.**

The dried cells were suspended with 80% aq. methanol (200 mL) and extracted by sonication (BANDELIN SONOPULS HD2200, BANDELIN electoronic GmbH, Berlin). The extracts were centrifuged and the residues were repeatedly extracted with 80% aq. methanol (*v*/*v*, 200 mL × 2) and 100% methanol (200 mL × 1). The extracts were combined and concentrated under reduced pressure. The residue was partitioned with

*n*-hexane (100 mL) and 90% aq. Methanol (*v*/*v*, 100 mL). After the solvent of 90% aq. methanol phase was removed under reduced pressure, the residue was subjected to reversed-phase flash column chromatography (110 × 30 mm, LiChroprep RP-18, 40–63 µm, MERCK) with eluents of 20, 40, 60, 80% aq. methanol, 100% methanol, and dichloromethane. The 80% aq. methanol eluted fraction was subjected to reversed-phase HPLC (Nucleosil 100-7C18, 250 × 21.2 mm, Macherey-Nagel) using a gradient system; solvent A (water containing 0.1% trifluroacetic acid), solvent B (83% aq. acetonitrile), 10% B for 10 min, to 100% B for 30 min, and kept for 10 min at a flow rate 12 mL $min^{-1}$, to yield crude nostopeptolide KVJ3 (1.0 mg). This crude peptide was subjected to reversed-phase HPLC (Nucleodur C18 ec, 250 × 10 mm, Macherey-Nagel) using a gradient system; solvent A (water containing 0.1% trifluroacetic acid), solvent B (83% aq. acetonitrile), 10% B for 10 min, to 100% B for 30 min, and kept for 10 min at a flow rate 5 mL $min^{-1}$, to yield nostopeptolide KVJ3 (**5**, 257 µg).

**Nostopeptolide KVJ3 (7)**. White powder. HRESIMS: $[M + H]^+$ = 964.4655 (calculated for $C_{48}H_{66}N_7O_{14}$, 964.4662).

**Detection of homoserine obtained by $LiBH_4$ reduction of minutumamide A1 (1)**. $LiBH_4$ (4.6 µg in dry THF) was added to minutumamide A1 (**1**, 100 µg) in dry THF (1 mL) at 0 °C and stirred for 2 h. After 0.5 M HCl (1 mL) was added to the reaction mixture, the mixture was further diluted with water and then subjected to SPE column (Isolute, ENV+, 100 mg, Separtis GmbH, Germany). The column was washed with water and peptides were eluted with methanol. The reduced peptide was dissolved in 6 M HCl (1 mL) and heated at 110 °C for 16 h. After acidic solution was dried under nitrogen gas stream, 1 M $NaHCO_3$ (100 µL) and Sanger's reagent (1-fluoro-2,4-dinitrobenzene, 50 µL (10 mg $mL^{-1}$ in acetone)) were added to the residue and then heated at 50 °C for 1 h. The reaction mixture was acidified by 2 M HCl (50 µL) and diluted with acetonitrile (250 µL).

The Sanger's reagent derivatives were analyzed by LC-MS (MS; AmaZon speed, Bruker Daltonics, LC; 1260 infinity, Agilent Technologies) using a reversed-phase HPLC column (Develosil RPAQUEOUS-AR-5 C30, 140Å, 4.6 × 150 mm, (Nomura Chemical) with a gradient system: solvent A (water containing 0.1% formic acid), solvent B (acetonitrile), 10% B for 2 min, to 100% B for 20 min. Flow rate: 1.0 mL $min^{-1}$.

β-Homoserine was prepared as follows: $LiBH_4$ (60 mg) was added to a solution of Boc-Asp-$O^tBu$ (200 mg) in dry THF (10 mL) and heated at 60 °C for 8 h. The reaction mixture was cooled in an ice bath and acidified with excess 0.5 M HCl followed by extracted with EtOAc. Organic phase was washed with brine and dried over $Na_2SO_4$. The obtained alcohol was subjected to reversed-phase HPLC (Nucleodur PolarTec, 5 µm, 100Å, 10 × 250 mm, Macherey-Nagel) using a gradient system, solvent A (water containing 0.1% TFA (*v*/*v*), solvent B (83% aq. acetonitrile), 5% B for 10 min, to 100% B in 30 min, at a flow rate 6 mL $min^{-1}$, to yield Boc-β-homoserine (79 mg). Boc-β-homoserine (7.0 mg) was dissolved in TFA/$H_2O$ (95:5, 500 µL) and stirred at room temperature for 20 min. The solvent was removed under nitrogen gas stream to obtain β-homoserine (3.6 mg, HRESIMS: $[M + H]^+$ = 120.0657 (calculated for $C_4H_{11}NO_3$,

120.0655). $^{1}H$ NMR (500 MHz, $D_2O$): $\delta_H$ = 4.73 (dd, $J$ = 11.3, 6.4 Hz, 1H), 4.55 (dd, $J$ = 11.3, 2.2 Hz, 1H), 4.39 (ddt, $J$ = 8.5, 6.4, 2.4 Hz, 1H), 3.23 (dd, $J$ = 19.0, 8.5 Hz, 1H), 2.80 (dd, $J$ = 19.0, 2.5 Hz, 1H).

**HPLC analysis of FDAA derivatives of amino acid hydrolysates.**
The peptides (minutumamides (**1–4**); 100 µg, nostopeptolide KVJ3 (**7**); 50 µg) were dissolved in 6 M HCl (500 µL) containing 0.05 % phenol and heated at 110 °C under argon for 16 h. After the removal of solvent under reduced pressure, the residue was dissolved in 1 M $NaHCO_3$ (100 µL) and 50 µL of FDAA solution (1-fluoro-2-4-dinitrophenyl-5-L-alanine amide, 10 mg $mL^{-1}$ in acetone) and incubated at 50 °C for 1 h. The reaction mixture was neutralized by 2 M HCl (50 µL) and diluted with 200 µL of 50% aq. acetonitrile (100 µL for nostopeptolide KVJ3).
The FDAA derivatives of amino acid hydrolysates were analyzed by LC-MS (MS; 6120 single quadrupole or AmaZon speed, LC; 1260 infinity, Agilent Technologies) using a gradient system as follows:
**L-FDAA derivative of D,L-Asp, D,L-Thr and D,L-alloThr.** HPLC column: Develosil RPAQUEOUS-AR-5 C30, 140Å, 4.6 × 150 mm. A gradient system: solvent A (water containing 0.1% formic acid), solvent B (acetonitrile), 20% B for 2.5 min, to 30% B for 5 min and kept 17.5 min, to 50% B for 20 min, to 20% B for 1 min. Flow rate: 1.0 mL $min^{-1}$.
**L-FDAA derivative of D,L-Tyr.** HPLC column: Develosil RPAQUEOUS-AR-5 C30, 140Å, 4.6 × 150 mm. A gradient system: solvent A (water containing 0.1% formic acid), solvent B (acetonitrile), 10% B to 100% B in 40 min, to 10% B for 1 min. Flow rate: 1.0 mL $min^{-1}$.
**L-FDAA derivative of D,L-Ser and D,L-FDAA derivative of Hmp.** HPLC column: Eclipse XDB-18, 3.5 µm, 80Å, 4.6 × 100 mm (Agilent Technologies). A gradient system: solvent A (water containing 0.1% formic acid), solvent B (acetonitrile), 20% B for 5 min, to 100% B in 25 min, to 20% B for 1 min. Flow rate: 1.0 mL $min^{-1}$.

**Purification of Hmp and Appo (2-amino-1-phenylpentan-3-one) from acid hydrolysate of minutumamide A2 (2).**
Minutumamide A2 (**2**, 15 mg in 0.5 mL methanol) was dissolved in 6 M HCl (6 mL) and removed air under reduced pressure. After the reaction mixture was heated at 110 °C for 18 h, the solvent was removed under reduced pressure followed by lyophilisation. The residue was subjected to reversed-phase HPLC (Synergy™ 4 µm Hydro RP 80Å, C18, 21.2 × 250 mm, Phenomenex) using a gradient system; solvent A (water containing 0.1% TFA), solvent B (83% aq. acetonitrile), 0%B for 15 min, to 100%B for 30 min, flow rate 12 mL $min^{-1}$, to yield Hmp (2.35 mg) and Appo (2.75 mg).

**Hmp (3-hydroxy-4-methylproline).** HRESIMS: $[M + H]^+$ = 146.0810 (calculated for $C_6H_{12}NO_3$, 146.0812). $^{1}H$ NMR (600 MHz, $D_2O$), $\delta_H$ = 4.48 (brt, $J$ = 3.1 Hz, 1H), 4.42 (brd, $J$ = 3.1 Hz, 1H), 3.50 (m, 1H), 3.06 (t, $J$ = 11.6 Hz, 1H), 2.42 (m, 1H), 1.02 (d, $J$ = 6.7 Hz, 3H).

**Appo (2-amino-1-phenylpentan-3-one).** HRESIMS: $[M + H]^+$ = 178.1226 (calculated for $C_{11}H_{16}NO$, 178.1226). $^1H$ NMR (600 MHz, $CD_3OD$), $\delta_H$ = 7.34 (td, $J$ = 7.4, 1.4 Hz, 2H), 7.28 (td, $J$ = 7.4, 1.4 Hz, 1H), 7.25 (dd, $J$ = 7.4, 1.4 Hz, 2H), 4.35 (dd, $J$ = 8.0, 6.6 Hz, 1H), 3.22 (dd, $J$ = 14.4, 6.6 Hz, 1H), 3.00 (dd, $J$ = 14.4, 8.0 Hz, 1H), 2.45 (m, 2H), 0.98 (t, $J$ = 7.2 Hz, 3H).

**CD spectrometer analysis of the *in situ* Hmp-fluorescamine derivative.**
The fluorescamine derivatization of Hmp was performed by the method of Toome *et al.*[3] Fluorescamine solution (250 µL, 0.004 M in dioxane) was mixed with an imino acid solution (250 µL, 0.002 M in 0.05 M potassium borate buffer, pH 9.0) in 1.5 mL Eppendorf tube and the mixture was vortexed for 15 sec. The resulting mixture was transferred to a 0.1 cm cuvette and measured by J-815 CD spectrometer (JASCO) at 20 °C.

**Preparation of BPG-Appo.**
Appo (0.5 mg, 2.8 µmol), HATU (1.3 mg, 3.4 µmol), and Boc-L-phenylglycine (1.0 mg, 4.0 µmol) were dissolved in dry DMF (0.5 mL). After the mixture was cooled in an ice bath, *N*,*N*-diisopropylethylamine (0.6 µL, 3.4 µmol) was added to the mixture and then the reaction mixture was stirred at room temperature for 20 h. The solvent was removed under reduced pressure and then the residue was dissolved in EtOAc and washed with water. The organic phase was dried over $Na_2SO_4$. The extract was subjected to reversed-phase HPLC (Nucleodur HTec, 5 µm, 100 Å, 10 × 250 mm) using a gradient system, solvent A (water containing 0.1 %TFA), solvent B (83% aq. acetonitrile (*v/v*)), 30% B for 10 min, to 100% B in 35 min, at flow rate 5 mL $min^{-1}$, to yield L-BPG-Appo (0.59 mg). D-BPG-Appo (0.67 mg) was obtained by the above-mentioned procedure.

**Detection of phenylalanine by retro Claisen reaction of peptides.**
The peptide (minutumamides (**1**–**4**), each 100 µg, nostopeptolide KVJ3 (**7**), 50 µg) was dissolved in 2 M NaOH (0.2 mL) and stirred at room temperature for 2 h. The reaction mixture was acidified with 1 M HCl (0.4 mL) and then subjected to SPE column (SDB, 50 mg, BAKERBOND) and washed with water. The methanol eluted fraction was dried under reduced pressure. The residue was dissolved in 6 M HCl (0.5 mL) and heated at 110 °C for 16 h. After the solvent was removed under reduced pressure, 1 M $NaHCO_3$ (100 µL) and L-FDAA (50 µL, 10 mg $mL^{-1}$ in acetone) were added and incubated at 50 °C for 1 h. The mixture was quenched with 2 M HCl (50 µL) and diluted with 50% aq. acetonitrile (100 µL). The L-FDAA derivatives of acid hydrolysates were analyzed by LC-MS (MS; AmaZon speed, LC; Agilent 1260 infinity) using the reversed-phase HPLC column (Eclipse XDB-C8, 5 µ, 80Å, 4.6 × 150 mm, Agilent Technologies) with a gradient system: solvent A (water containing 0.1% formic acid), solvent B (acetonitrile), 10% B for 2 min, to 100% B for 20 min. Flow rate: 1.0 mL $min^{-1}$.

**The stereochemistry elucidation of 4-amino-3-oxo-2-methyl-5-phenylvaleric acid (Amopa).**

The peptides (minutumamides (**1**–**4**), each 100 µg for Phe, 500 µg for lactic acid) were dissolved in TFA (200 µL for Phe, 500 µL for lactic acid) and then cooled in an ice bath. A portion of $Na_2CO_4$ (excess) was added separately twice in 20 min and the mixture was stirred at 0 °C for further 10 min followed by room temperature for 3 h. The reaction mixture was concentrated under nitrogen gas stream, diluted with water and then subjected to SPE column (SDB, 200 mg, BAKERBOND). After the resin was washed with water, the oxidized peptide was eluted with methanol. The resulting peptide was dissolved in 6 M HCl (1 mL) and heated at 110 °C for 16 h. The acid hydrolysate was dried under nitrogen gas stream.

**Derivatization and detection of Phe.** To the dried acid hydrolysate, 1 M $NaHCO_3$ (100 µL) and L-FDAA (50 µL, 10 mg mL$^{-1}$ in acetone) were added and heated at 50 °C for 1 h. After the reaction mixture was neutralized with 2 M HCl (50 µL), the L-FDAA derivatives of acid hydrolysates were analyzed by LC-MS (MS; AmaZon speed, LC; 1260 infinity) using the reversed-phase HPLC column (Eclipse XDB-C8, 5 µm, 80Å, 4.6 × 150 mm) with a gradient system: solvent A (water containing 0.1% formic acid), solvent B (acetonitrile), 10% B for 2 min, to 100% B for 20 min. Flow rate: 1.0 mL min$^{-1}$.

**Derivatization and detection of lactic acid.** The dried acid hydrolysate was dissolved in dry THF (1 mL). After sodium hydride (3 mg, 60% dispension in mineral oil) was added to the mixture, this mixture was stirred under argon at room temperature for 10 min. L-FDAA (200 µL, 8 mg mL$^{-1}$ in dry THF) was added to the mixture and further stirred at room temperature for 2 h. After the reaction mixture was acidified with 2 M HCl (3 mL), the lactic acid derivative was extracted with EtOAc (2 mL × 3). The obtained organic phase was dried over $Na_2SO_4$ and concentrated under reduced pressure. The residue was dissolved in methanol (100 µL). The L-FDAA derivatives of hydroxy acids were analyzed by LC-MS (MS; AmaZon speed, LC; 1260 infinity) using the reversed-phase HPLC column (Eclipse XDB-C18, 3.5 µ, 80Å, 4.6 × 100 mm) with a gradient system: solvent A (water containing 0.1% formic acid), solvent B (acetonitrile), 20% B for 5 min, to 100% B in 25 min, to 20% B for 1 min. Flow rate: 1.0 mL min$^{-1}$.

**Bioinformatic analysis**

For the comparison of gene segments that encode NRPS and PKS modules, π values (average number of nucleotide differences per site between two sequences) were computed in the sliding window mode in DnaSP 6[4] for sequence pairs that had been prealigned with Mega X.[5] The sliding window varied between a width of 300 nt and 1000 nt according to the size of the analyzed gene segments and had a step size of 25 nt. For visualization of sequence similarity/divergence, π values were plotted against window midpoints with Microsoft Excel.

Module and domain boundaries have been inferred by using the PKS/NRPS Analysis Website of the University of Maryland.[6] Boundaries of docking domains (dd) have been inferred manually by sequence comparison to the prototype docking domains TubB_$^{C}$dd, and TubC_$^{N}$dd.[7]

Evolutionary analyses were conducted in MEGA X.[5] The evolutionary history was inferred using the Neighbor-Joining method.[8] The percentage of replicate trees in

which the associated sequence clustered together in the bootstrap test[9] (500 replicates) are shown next to the branches. The trees were drawn to scale, with branch lengths in the same units as those of the evolutionary distances used to infer the phylogenetic trees. The evolutionary distances were computed using the Poisson correction method.[10]

## 2. Supplemental Notes

### Discovery and structure elucidation of minutumamides

*Nostoc minutum* NIES-26 was cultured in 10 L glass bottles to a final volume of 590 L. Next, cells were harvested and lyophilized. The dried cells were extracted with aqueous methanol and the extracts were subjected to ODS flash column chromatography followed by reversed-phase HPLC, yielding minutamamide A1 and A2 as a major and a minor component, respectively. Minutumamide B1 and B2 were isolated from *Nostoc linckia* NIES-25 by using the same procedure as mentioned above. The positive ion *m/z* 856.3784 $[M + H]^+$ observed by the high-resolution LC-MS showed that minutumamide A1 (**1**) has a molecular formula of $C_{45}H_{53}N_5O_{12}$ (Fig. S1). Its peptidic nature was confirmed by the observation of amide protons Thr-NH (δ 7.79), Amopa-NH (δ 8.90), Tyr (δ 7.27), and Asp-NH (δ 8.49) and seven amide (ester) carbonyl carbons between δ 166.1 and 172.6 in $^1H$ and $^{13}C$ NMR spectrum (Fig. S2, S3). The three proteinogenic amino acid residues of Thr, Tyr, and Glu were determined by $^1H$-$^1H$ COSY, HSQC, and HMBC spectral analysis (Fig. S4–S6). Detailed analysis of $^1H$-$^1H$ COSY, HSQC, and HMBC spectra indicated the presence of two phenyl rings by aromatic protons between δ 7.11 and 7.26 (Fig. S4–S6). A moiety possessing the first phenyl ring was determined as 4-phenylbutyric acid (Pba) by HMBC correlations from Pba-H3 (*δ* 1.80) to Pba-C1 (*δ* 172.6), Pba-C2 (*δ* 34.7), Pba-C4 (*δ* 34.5), Pba-C5 (*δ* 141.9), and $^1H$-$^1H$ COSY correlations from Pba-H2 (*δ* 2.28) to Pba-H4 (*δ* 2.56). Another phenyl group belongs to 4-amino-3-oxo-2-methyl-5-phenylvaleric acid (Amopa), which consists of a phenylalanyl and a propyl unit. Connectivity of these units was determined by the HMBC correlations between Amopa-H12 (*δ* 0.85), -H4 (*δ* 4.78), -H5 (*δ* 2.66 and *δ* 3.01)/Amopa-C3 (*δ* 202.9). The connectivity of 4-methyl-3-hydroxyproline (Hmp) was determined by the spin system between Hmp-H2 (*δ* 4.58)/Hmp-H3 (*δ* 4.40), Hmp-H3/Hmp-H4 (*δ* 2.15), Hmp-H4/Hmp-H5 (*δ* 3.17, 3.89), Hmp-H3/Hmp-OH (*δ* 5.85), Hmp-H4/Hmp-H6 (*δ* 1.00) in $^1H$-$^1H$ COSY and confirmed by the HMBC correlations between Hmp-H5/Hmp-C2 (*δ* 64.8) and Hmp-H2/Hmp-C1 (*δ* 168.2). The sequence of amino acids was elucidated by the HMBC correlations between Thr-H2/Pba-C1, Hmp-H5/Thr-C1, Ampa-H4/Hmp-C1, Tyr-H2/Amopa-C1, Thr-H2/Asp-C4 (Fig. S8), to feature a cyclic pentadepsipeptide. Since α- and γ-carbonyl ester formation of Asp with Thr cannot be distinguished by HMBC correlations, minutumamide A1 (**1**) was reduced by $LiBH_4$ followed by acid hydrolyzation (Fig. S9). Analysis of the Sanger's reagent derivatives of acid hydrolysates by LC-MS led to the detection of homoserine. From this result it was determined that minutumamide A1 (**1**) possesses an ester bond between a hydroxy group of Thr and the γ-carbonyl group of Asp (Fig. S9).

Minutumamide A2 (**2**) possesses the same molecular formula as $C_{45}H_{53}N_5O_{12}$ (Fig. S10) and extensive 1D and 2D NMR spectral analysis showed that minutumamide A2 has the same planar structure as minutumamide A1 (**1**, Fig. S11–S17).

The molecular formula $C_{45}H_{51}N_5O_{12}$ obtained from positive ion 854.3600 $[M + H]^+$ (Fig. S18) and 1D NMR spectra of minutumamide B1 (**2**) suggested that the structure of this peptide is very similar to that of minutumamide A1 (**1**, Fig. S19–S20). The detailed

analysis of 1D and 2D NMR spectral data revealed that minutumamide B1 has 4-phenyl butanoic acid (Dpba) instead of 4-phenyl butyric aicd (Pba) as in minutumamide A1 and A2 (Fig. S19–S25). Interestingly, as in the case of minutumamide A1 (**1**) and A2 (**2**), minutumamide B2 (**4**) also has the same planer structure as minutumamide B1 (**3**, Fig. S26–S33).

The stereochemistry of proteinogenic amino acids in the four minutumamides (**1**–**4**) was elucidated by reversed-phase HPLC-MS analysis of acid hydrolysate of peptides treated with L-FDAA (α-(2,4-Dinitro-5-fluorophenyl)-L-alaninamide), as L-Thr, L-Asp, and L-Tyr (Fig. S34–S36)[11]. To elucidate the absolute stereochemistry of Hmp and Amopa, a large-scale acid hydrolyzation of minutumamide A2 was performed to afford Hmp and Appo (2-amino-1-phenylpentan-3-one) (Fig. S41). Hmp purified by reversed phase HPLC was derivatized with fluorescamine and its derivative was analyzed by CD spectrometry[3]. By the observation of positive (317 nm) and negative (317 nm) cotton effects as those of L-Pro, the absolute stereochemistry at C2 of Hmp was determined as *S* configuration (Fig. S37). The vicinal small coupling constants, $^{3}J_{H2,H3}$ (3.1 Hz) and $^{3}J_{H3,H4}$ (3.1 Hz) of purified Hmp, and observed NOEs between H2/H4 and OH/H5 of Hmp in minutumamide A2 (**2**) revealed that the stereochemistry of Hmp is 2*S*, 3*R*, 4*R* (Fig. S16, S38, S39). The very similar chemical shifts of Hmp among minutumamides suggest the same stereochemistry of Hmp in the other three minutumamide congeners (A2, B1, and B2). This suggestion is also supported by Marfey analysis using L- and D-FDAA derivatives of Hmp obtained from minutumamide A2 (Fig. S40).

To elucidate the stereochemistry of the amino group in Appo, the amine was derivatized by the chiral reagent BPG (Boc-phenylglycine)[12]. $^{1}$H NMR spectra indicated that Appo was a racemic mixture (Fig. S41–S42). This racemization was observed by retro Claisen reaction[13] of Amopa as D,L-phenylalanine mixture (Fig. S43–S44). Finally, we succeeded to elucidate the stereochemistry of Amopa by using a Baeyer Villger oxidation (BVO). For this, minutumamide A1 (**1**) was oxidized by $Na_2CO_4$ in TFA[14] and hydrolyzed by 6M HCl. Obtained acid hydrolysates were derivatized with L-FDAA and the derivatives were analyzed by HPLC-MS to afford L-Phe and L-lactic acid[15] (Fig. S45). Thus, the stereochemistry of Amopa was elucidated as 2*R*, 4*S*. The absolute stereochemistry of Amopa in the other three minutumamides was also determined as 2*R*, 4*S* configuration by performed BVO (Fig. S44–S45).

The same absolute stereochemistry between minutumamide A1 (**1**) and A2 (**2**) (B1 (**3**) and B2 (**4**)) suggested that these peptides are conformational isomers. Thus, we investigated temperature-dependent $^{1}$H NMR signals of amide protons. Baxter and Williamson have reported that a hydrogen-bonded amide proton in proteins has a more positive temperature coefficient ppb/K than –4.5, but a non-hydrogen bonded amide proton has a more negative ppb/K than –4.5.[16] Minutumamide A1/B1 and A2/B2 showed very similar amide proton chemical shift change and temperature coefficient pattern, respectively (Fig. S46–S49, Table S5). In minutumamide A1 (**1**) and B1 (**3**), only Asp clearly has a hydrogen-bonded amide proton. On the other hand, Asp and Tyr have hydrogen-bonded amide protons in minutumamide A2 (**2**) and B2 (**4**) (Fig. S50, Table S5). These differences strongly suggest a *cis-trans* amide bond between Thr and Hmp. The observed long-range NOEs in minutumamide A2 (**2**) and B2 (**4**) (α,

β-proton of Thr to α-proton Amopa), which were not detected in minutumamide A1 (**1**) and B1 (**3**), support the assumption of rotamers (minutumamide A1/A2 and B1/B2) with a *cis*-*trans* amide bond (Fig. S51).

**Discovery and structure elucidation of nostopeptolide KVJ3 (7)**

The freeze-dried cells of *Nostoc* sp. KVJ3 were extracted with aqueous methanol and concentrated under reduced pressure. The residue was partitioned with *n*-hexane and 90% aq. methanol. The aq. methanol phase was subjected to reversed phase column chromatography followed by reversed phase HPLC to yield small amounts of nostopeptolide KVJ3 (**7**).

The positive ion 964.4655 $[M+H]^+$ of nostopeptolide KVJ3 (**7**) observed by high-resolution LCMS analysis revealed the molecular formula $C_{48}H_{65}N_7O_{14}$ (Fig. S56). The $^1H$ NMR spectrum showed the presence of two aromatic rings, *p*-hydroxyphenyl group signals $\delta_H$ 6.60 (2H, d, $J$ = 8.5 Hz) and 6.99 (2H, d, $J$ = 8.5 Hz), and phenyl group signals $\delta_H$ 7.26 (2H, d, $J$ = 7.3 Hz), 7.16 (2H, t, $J$ = 7.3 Hz), and 7.10 (1H, t, $J$ = 7.3 Hz) (Fig. S57). These moieties were confirmed as Tyr and Amopa, respectively, by analysis of $^{13}C$ NMR, $^1H$-$^1H$ COSY, HSQC, and HMBC (Fig. S35, S58–S61, S63). In the high field of the $^1H$ NMR spectrum, four doublet methyl protons and one triplet methyl proton were observed. One doublet methyl proton $\delta_H$ 0.95 (d, $J$ = 6.4 Hz) belongs to Amopa. The three independent spin systems, Thr-NH $\delta_H$ 8.40 to Thr-H4 $\delta_H$ 1.13 (d, $J$ = 6.3 Hz), Hmp1-H2 $\delta_H$ 4.51 to Hmp1-H6 $\delta_H$ 0.98 (d 6.7), and Hmp2-H2 $\delta_H$ 4.26 to H6 $\delta_H$ 0.93 (d, $J$ = 6.7 Hz), observed by $^1H$-$^1H$ COSY indicated that the other three methyl protons belong to Thr, Hmp1, and Hmp2, respectively, and these moieties were confirmed by HMBC correlations. $^1H$-$^1H$ COSY correlations from triplet methyl protons Ha-H6 $\delta_H$ 0.85 (d, $J$ = 6.8 Hz) to methylene protons Ha-H2 $\delta_H$ 2.20/2.28 and HMBC correlations between Ha-H2/H3 $\delta_H$ 1.50 and Ha-C1 $\delta_C$ 172.9 revealed that this unit is a hexanoic acid (Ha). The remaining two $^1H$-$^1H$ COSY spin systems Gly-NH $\delta_H$ 7.93 to Gly-H2 $\delta_H$ 3.59/3.76 and Ser-NH $\delta_H$ 7.59 to Ser-H3 $\delta_H$ 3.68/3.78 were determined as Gly and Ser residues, respectively. Finally, the obtained 8 residues were connected by HMBC correlations between Thr-H2 $\delta_H$ 4.92/Ha-C1, Hmp1-H2/Thr-C1 $\delta_C$ 166.4, Amopa-NH $\delta_H$ 8.54, Ser-NH $\delta_H$ 7.59/Amopa-C1 $\delta_C$ 167.9, Hmp2-H2/Ser-C1 $\delta_C$ 173.1, Try-NH $\delta_H$ 7.44/Hmp2-C1 $\delta_C$ 168.3, Gly-NH/Tyr-C1 $\delta_C$ 172.4, Thr-H3 $\delta_H$ 5.32/Gly-C1 $\delta_C$ 168.9, yielding the structure of the novel cyclic heptadepsipeptide nostopeptolide KVJ3 (**5**, Fig. 1 and S63).

The stereochemistry of proteinogenic amino acids was elucidated as L-Thr, L-Ser, and L-Tyr by reversed-phase HPLC-MS analysis of L-FDAA derivatives of the acid hydrolysate of nostopeptolide KVJ3 (Fig. S34–S36). The stereochemistry of both Hmp residues was deduced as 2*S*, 3*R*, 4*R*, judging from the similarity of chemical shifts with those of the minutumamides.

**Detailed comparison of the nostopeptolide A1 and nostopeptolide KVJ3 BGC**

To investigate the divergence of both clusters on the genomic level we compared gene segments that encode NRPS modules by sliding window analysis to compute $\pi$ values (the average number of nucleotide differences per site between two sequences). Segments with low $\pi$ values (near 0) correlate with high homology of sequences, whereas segments with high $\pi$ values (near 1) correlate with high divergence of sequences. Analogous to the protein level, the respective modules show high divergence in the segments encoding the A domain, with sequences encoding the specificity pocket being the most affected (Fig. S65a, b). Remarkably, the divergent sequences can be complemented by highly homologous sequences from other modules of the two clusters, thereby indicating inter-cluster gene shuffling (Fig. S65a, b). In addition to that, it seems that the nostopeptolide clusters were further diversified by three distinct cluster streamlining and/or expansion events, which affect side chain composition, ring size and methylation of the products. Strikingly, the sequence encoding the domain series $A_4T_4C_4A_5T_5$, which facilitates the incorporation of serine and hydroxymethylproline in the biosynthesis of nostopeptolide KVJ3 is highly homologous to the sequence encoding the domain series $A_2T_2C_2A_3T_3$, which facilitates the incorporation of serine and methylproline in the biosynthesis of nostopeptolide A1 (Fig. S65c). This indicates either an ancestral gene duplication event in the evolution of nostopeptolides that is veiled in the biosynthesis gene cluster of nostopeptolide A1 by extensive gene mutation and module expansion, or, more intuitively, a module swap comprising $A_5T_5C_6A_6T_6C_7A_7T_7$ of *nosC* and $A_2T_2C_2A_3T_3$ of *nosA* early in the evolution of the nostopeptolide KVJ3 BGC. This assumed module swap should then have occurred after an inter-cluster subdomain swap of $A_6$ of *nosC* and $A_9$ of *nosD* that led to a change of substrate specificity of the encoded A domain from proline to glycine but before the change of the substrate specificity of $A_2$ encoded by *nosA* from serine to threonine as found in the structure of nostopeptolide KVJ3. The latter assumption is supported by the observation that $A_4T_4$ of the nostopeptolide KVJ3 BGC shares much more homology to $A_2T_2$ of the nostopeptolide A1 BGC than to the corresponding didomain sequence of the own cluster (Fig. 3e). Regarding the additional methyltransferase (MT) domain of $NosB_{KVJ3}$, remnants of a MT domain between the AT and ACP domains of NosB from *Nostoc* sp. GSV224 can be found, what is a clear sign of the loss of a MT domain in the evolution of nostopeptolide A1 rather than the gain of a MT domain in the evolution of nostopeptolide KVJ3. Although it is impossible to unambiguously recapitulate the evolutionary history of nostopeptolides with only two types of congeners and BGCs at hand, the most parsimonious model would suggest a series of module, domain and subdomain swaps as well as domain deletions leading to the remodeled, minimized biosynthetic pathway of nostopeptolide KVJ3 (Fig. S65). Hopefully, more congeners and corresponding BGCs will be elucidated in the future that could help to further inform and test this model.

## 3. Supplemental Tables

**Table S1.** $^{1}H$ (600 MHz) and $^{13}C$ (150 MHz) NMR data for minutumamide A1 (**1**) in DMSO-$d_6$

| position | $^{13}C$ (type) | $^{1}H$, m ($J$ Hz) | $^{1}H$-$^{1}H$ COSY | HMBC (**$^{1}H$** to $^{13}C$) |
|---|---|---|---|---|
| **Pba** | | | | |
| 1 | 172.6 (C) | | | Pba-H2, H3, Thr-H2 |
| 2 | 34.7 ($CH_2$) | 2.28, m | Pba-H3 | Pba-H3, H4 |
| 3 | 27.5 ($CH_2$) | 1.80, m | Pba-H2, H4 | Pba-H2, H4 |
| 4 | 34.5 ($CH_2$) | 2.56, m | Pba-H3 | Pba-H2, H3, H6, H10 |
| 5 | 141.9 (C) | | | Pba-H4, H7, H9 |
| 6,10 | 128.1 (CH) | 7.18, d (7.5) | Pba-H6, H7, H9, H10 | Pba-H4, H6, H10 |
| 7,9 | 128.2 (CH) | 7.26, t (7.5) | Pba-H6, H7, H8, H9, H10 | Pba-H7, H9 |
| 8 | 125.7 (CH) | 7.16, t (7.5) | Pba-H7, H9 | Pba-H6, H10 |
| **Thr** | | | | |
| 1 | 166.1 (C) | | | Thr-H2, Hmp-H5 |
| 2 | 53.6 (CH) | 4.97, dd (8.5, 2.6) | Thr-H3, NH | Thr-H4 |
| 3 | 69.4 (CH) | 5.28, br | Thr-H2, H4 | Thr-H4 |
| 4 | 16.4 ($CH_3$) | 1.16, d (6.2) | Thr-H3 | |
| NH | | 7.79, d (8.5) | Thr-H2 | |
| **Hmp** | | | | |
| 1 | 168.2 (C) | | | Hmp-H2 |
| 2 | 64.8 (CH) | 4.58, d (6.0) | Hmp-H3 | Hmp-H4, H5 |
| 3 | 72.3 (CH) | 4.40, m | Hmp-H2, H4, OH | Hmp-H4, H5, H6 |
| 4 | 39.2 (CH) | 2.15, m | Hmp-H3, H5, H6 | Hmp-H3, H5, H6 |
| 5 | 51.2 ($CH_2$) | 3.17, m | Hmp-H4, H5 | Hmp-H3, H4, H6 |
| | | 3.89, dd (9.2, 6.8) | Hmp-H4, H5 | |
| 6 | 10.4 ($CH_3$) | 1.00, d (6.7) | Hmp-H4 | |
| OH | | 5.85, br | Hmp-OH | |
| **Amopa** | | | | |
| 1 | 169.0 (C) | | | Amopa-H12, Tyr-H2 |
| 2 | 47.6 (CH) | 4.15, m | Amopa-H12 | Amopa-H12 |
| 3 | 202.9 (C) | | | Amopa-H4, H5, H12 |
| 4 | 55.3 (CH) | 4.78, br | Amopa-H5, NH | Amopa-H5 |
| 5 | 34.9 ($CH_2$) | 2.66, dd (14.6, 7.9) | Amopa-H4, H5 | Amopa-H7, H11 |
| | | 3.01, dd (14.6, 3.9) | Amopa-H4, H5 | |
| 6 | 137.4 (C) | | | Amopa-H5 |
| 7,11 | 129.0 (CH) | 7.17, m | Amopa-H7, H8, H10, H11 | Amopa-H5, H7, H9, H11 |
| 8,10 | 128.1 (CH) | 7.19, m | Amopa-H7, H8, H9, H10, H11 | Amopa-H8, H10 |
| 9 | 125.9 (CH) | 7.11, t (7.0) | Amopa-H8, H10 | Amopa-H7, H11 |
| 12 | 12.7 ($CH_3$) | 0.8, d (6.7) | Amopa-H2 | Amopa-H2 |
| NH | | 8.90, brd (9.0) | Amopa-H4 | |
| **Tyr** | | | | |
| 1 | 171.3 (C) | | | Tyr-H2 |
| 2 | 55.7 (CH) | 4.16, m | Tyr-H3, NH | Tyr-H3 |
| 3 | 37.0 ($CH_2$) | 2.48, m | Tyr-H2, H3 | Tyr-H2, H5, H9 |
| | | 2.99, m | Tyr-H2, H3 | |
| 4 | 127.8 (C) | | | Tyr-6, H8 |
| 5,9 | 129.8 (CH) | 6.99, d (8.5) | Tyr-H5, H6, H8, H9 | Tyr-H3, H5, H6, H8, H9 |
| 6,8 | 115.2 (CH) | 6.63, d (8.5) | Tyr-H5, H6, H8, H9 | Tyr-H6, H8, OH |
| 10 | 155.9 (C) | | | Tyr-H5, H6, H8, H9 |
| OH | | 9.20, s | | |
| NH | | 7.27, m | Tyr-H2 | |
| **Asp** | | | | |
| 1 | 172.3 (C) | | | Asp-H3 |
| 2 | 49.2 (CH) | 4.32, br | Asp-H3, NH | Asp-H3 |
| 3 | 33.8 (CH) | 2.35, m | Asp-H2, H3 | |
| | | 2.54, m | Asp-H2, H3 | |
| 4 | 169.1 (C) | | | Asp-H3, Thr-H3 |
| NH | | 8.49, brs | Asp-H2 | |

**Table S2.** $^{1}H$ (600 MHz) and $^{13}C$ (150 MHz) NMR data for minutumamide A2 (**2**) in DMSO-$d_6$

| position | $^{13}C$ (type) | $^{1}H$, m ($J$ Hz) | $^{1}H$-$^{1}H$ COSY | HMBC ($^{1}$**H** to $^{13}C$) |
|---|---|---|---|---|
| **Pba** | | | | |
| 1 | 172.8 (C) | | | Pba-H2, H3, Thr-H2 |
| 2 | 34.7* ($CH_2$) | 2.30, m | Pba-H3 | Pba-H3, H4 |
| 3 | 27.4 ($CH_2$) | 1.81, m | Pba-H2, H4 | Pba-H2, H4 |
| 4 | 34.8 ($CH_2$) | 2.58, brt (7.6) | Pba-H3 | Pba-HH2, H3, H6, H10 |
| 5 | 142.0 (C) | | | Pba-H3, H4, H7, H9 |
| 6,10 | 128.4 (CH) | 7.19, d (7.5) | Pba-H6, H7, H9, H10 | Pba-H4, H6, H8, H10 |
| 7,9 | 128.3 (CH) | 7.27, t (7.5) | Pba-H6, H7, H8, H9, H10 | Pba-H7, H9 |
| 8 | 125.7 (CH) | 7.17, t (7.5) | | Pba-H6, H10 |
| **Thr** | | | | |
| 1 | 166.6 (C) | | | Thr-H2, Hmp-H2, H5 |
| 2 | 53.8 (CH) | 5.01, dd (9.0, 2.9) | Thr-H3, NH | Thr-H4 |
| 3 | 68.9 (CH) | 5.32, m | Thr-H2, H4 | Thr-H2, H4 |
| 4 | 16.3 ($CH_3$) | 1.14, d (6.2) | Thr-H3 | Thr-H3 |
| NH | | 8.10, d (9.0) | Thr-H2 | |
| **Hmp** | | | | |
| 1 | 168.7 (C) | | | Hmp-H2, Amopa-H2 |
| 2 | 65.3 (CH) | 4.55, d (5.7) | Hmp-H3 | Hmp-H3, H4, H5 |
| 3 | 72.2 (CH) | 4.38, m | Hmp-H2, H4, OH | Hmp-H2, H5, H6 |
| 4 | 39.1 (CH) | 2.11, m | Hmp-H3, H5, H6 | Hmp-H2, H3, H5, H6 |
| 5 | 51.6 ($CH_2$) | 3.08, brt (10.9) | Hmp-H4, H5 | Hmp-H3, H4, H6 |
| | | 3.94, m | Hmp-H4, H5 | |
| 6 | 10.4 ($CH_3$) | 0.95, d (6.7) | Hmp-H4 | Hmp-H4, H5 |
| OH | | 5.88, br | Hmp-H2 | |
| **Amopa** | | | | |
| 1 | 168.3 (C) | | | Amopa-H12, Tyr-H2 |
| 2 | 46.0 (CH) | 4.05, q (6.9) | Amopa-H12 | Amopa-H12 |
| 3 | 204.6 (C) | | | Amopa-H4, H5, H12 |
| 4 | 58.9 (CH) | 3.93, m | Amopa-H5, NH | Amopa-H5 |
| 5 | 34.2 ($CH_2$) | 2.78, m | Amopa-H4, H5 | Amopa-H7, H11 |
| | | 3.06, dd (13.6, 3.0) | Amopa-H4, H5 | |
| 6 | 138.8 (C) | | | Amopa-H5, H8, H10 |
| 7,11 | 130.1 (CH) | 7.42, d (7.3) | Amopa-H7, H8, H10, H11 | Amopa-H5, H7, H9, H11 |
| 8,10 | 128.1 (CH) | 7.21, t (7.3) | Amopa-H7, H8, H9, H10, H11 | Amopa-H8, H10 |
| 9 | 126.2 (CH) | 7.15, t (7.3) | Amopa-H8, H10 | Amopa-H7, H11 |
| 12 | 14.0 ($CH_3$) | 1.00, d (6.9) | Amopa-H2 | Amopa-H2 |
| NH | | 8.94, m | Amopa-H4 | |
| **Tyr** | | | | |
| 1 | 171.7 (C) | | | Tyr-H2, H3, Asp-H2 |
| 2 | 55.1 (CH) | 4.29, ddd (10.6, 9.0, 3.5) | Tyr-H3, NH | Tyr-H3 |
| 3 | 37.0 ($CH_2$) | 2.40, dd (14.3, 10.6) | Tyr-H2, H3 | Tyr-H2, H5, H9 |
| | | 3.00, dd (14.3, 3.5) | Tyr-H2, H3 | |
| 4 | | | | Tyr-H2, H6, H8 |
| 5,9 | 128.6 (C) | 7.02, d (8.5) | Tyr-H5, H6, H8, H9 | Tyr-H3, H5, H9 |
| 6,8 | 129.7 (CH) | 6.62, d (8.5) | Tyr-H5, H6, H8, H9, H10 | Tyr-H5, H6, H8, H9, OH |
| 10 | 115.0 (CH) | | Tyr-H6, H8 | Tyr-H6, H8, OH |
| NH | 155.7 (C) | 7.15, m | Tyr-H2 | |
| OH | | 9.18, s | | |
| **Asp** | | | | |
| 1 | 171.8 (C) | | | Asp-H3 |
| 2 | 48.0 (CH) | 4.64, ddd (11.2, 8.2, 3.6) | Asp-H3, NH | Asp-H3 |
| 3 | 34.7* ($CH_2$) | 2.64, dd (17.7, 11.2) | Asp-H2, H3 | Asp-H2 |
| | | 2.76, dd (17.7, 3.6) | Asp-H2, H3 | |
| 4 | 169.4 (C) | | | Asp-H2, H3, Thr-H3 |
| NH | | 7.26, m | Asp-H2 | |

*: exchangeable

**Table S3.** $^1$H (600 MHz) and $^{13}$C (150 MHz) NMR data for minutumamide B1 (**3**) in DMSO-$d_6$

| position | $^{13}$C (type) | $^1$H, m ($J$ Hz) | $^1$H-$^1$H COSY | HMBC ($^1$**H** to $^{13}$C) |
|---|---|---|---|---|
| **Dpba** | | | | |
| 1 | 165.2 (C) | | | Dpba-H2, H3, Thr-H2, NH |
| 2 | 124.9 (CH) | 6.39, d (15.3) | Dpba-H3 | Dpba-H4 |
| 3 | 142.2 (CH) | 6.76, ddd (15.3, 7.0, 6.9) | Dpba-H2, H4 | Dpba-H4 |
| 4 | 37.2 ($CH_2$) | 3.47, d (6.9) | Dpba-H3 | Dpba-HH2, H3, H6, H10 |
| 5 | 138.7 (C) | | | Dpba-H3, H4 |
| 6,10 | 128.7 (CH) | 7.20, m | Dpba-H6, H7, H9, H10 | Dpba-H4, H6, H8, H10 |
| 7,9 | 128.5 (CH) | 7.31, t (7.6) | Dpba-H6, H7, H8, H9, H10 | Dpba-H7, H9 |
| 8 | 126.3 (CH) | 7.21, m | Dpba-H7, H9 | Dpba-H6, H10 |
| **Thr** | | | | |
| 1 | 165.0 (C) | | | Thr-H2, Hmp-H2, H5 |
| 2 | 53.8 (CH) | 5.03, dd (8.8, 2.8) | Thr-H3, NH | Thr-H4 |
| 3 | 69.2 (CH) | 5.29, m | Thr-H2, H4 | Thr-H4 |
| 4 | 16.4 ($CH_3$) | 1.14, d (6.2) | Thr-H3 | Thr-H3 |
| NH | | 8.00, d (9.1) | Thr-H2 | |
| **Hmp** | | | | |
| 1 | 168.1 (C) | | | Hmp-H2, Amopa-H4 |
| 2 | 64.8 (CH) | 4.56, d (6.1) | Hmp-H3 | Hmp-H3, H5 |
| 3 | 72.3 (CH) | 4.40, m | Hmp-H2, H4, OH | Hmp-H2, H5, H6 |
| 4 | 39.1 (CH) | 2.41, m | Hmp-H3, H5, H6 | Hmp-H2, H5, H6 |
| 5 | 51.1 ($CH_2$) | 3.17, m | Hmp-H4, H5 | Hmp-H2, H3, H4, H6 |
| | | 3.90, dd (9.5, 7.1) | Hmp-H4, H5 | |
| 6 | 10.3 ($CH_3$) | 1.00, d (6.8) | Hmp-H4 | Hmp-H4, H5 |
| OH | | 5.82, brs | Hmp-H3 | |
| **Amopa** | | | | |
| 1 | 169.1 (C) | | | Amopa-H2, H12, Tyr-H2 |
| 2 | 47.6 (CH) | 4.14, m | Amopa-H12 | Amopa-H12 |
| 3 | 202.7 (C) | | | Amopa-H2, H5, H12 |
| 4 | 55.5 (CH) | 4.80, br | Amopa-H5, NH | Amopa-H5 |
| 5 | 34.9 ($CH_2$) | 2.66, dd (4.7, 7.6) | Amopa-H4, H5 | Amopa-H7, H11 |
| | | 2.96, m | | |
| 6 | 137.4 (C) | | | Amopa-H5, H8, H10 |
| 7,11 | 128.9 (CH) | 7.16, m | Amopa-H7, H8, H10, H11 | Amopa-H5, H7, H9, H11 |
| 8,10 | 128.2 (CH) | 7.19, m | Amopa-H7, H8, H9, H10, H11 | Amopa-H8, H10 |
| 9 | 125.9 (CH) | 7.12, t (7.2) | Amopa-H8, H10 | Amopa-H7, H11 |
| 12 | 12.6 ($CH_3$) | 0.85, d (6.6) | Amopa-H2 | Amopa-H2 |
| NH | | 8.88, brd (8.9) | Amopa-H4 | |
| **Tyr** | | | | |
| 1 | 171.3 (C) | | | Tyr-H2, H3, Asp-H2 |
| 2 | 55.9 (CH) | 4.16, m | Tyr-H3, NH | Tyr-H3 |
| 3 | 37.0 ($CH_2$) | 2.50, m | Tyr-H2, H3 | Tyr-H2, H5, H9 |
| | | 2.99, m | Tyr-H2, H3 | |
| 4 | 127.8 (C) | | | Tyr-H2, H3, H5, H6, H8, H9 |
| 5,9 | 129.6 (CH) | 6.99, d (8.5) | Tyr-H5, H6, H8, H9 | Tyr-H3, H5, H9 |
| 6,8 | 115.0 (CH) | 6.63, d (8.5) | Tyr-H5, H6, H8, H9 | Tyr-H6, H8, OH |
| 10 | 155.8 (C) | | | Tyr-H5, H6, H8, H9, OH |
| OH | | 9.17, s | | |
| NH | | 7.24, m | Tyr-H2 | |
| **Asp** | | | | |
| 1 | 172.2 (C) | | | Asp-H2, H3 |
| 2 | 49.3 (CH) | 4.33, m | Asp-H3, NH | Asp-H3 |
| 3 | 33.6 ($CH_2$) | 2.38, dd (18.0, 13.0) | Asp-H2, H3 | |
| | | 2.55, m | Asp-H2, H3 | |
| 4 | 169.3 (C) | | | Asp-H2, H3, Thr-H3 |
| NH | | 8.48, brs | Asp-H2 | |

**Table S4.** $^{1}H$ (600 MHz) and $^{13}C$ (150 MHz) NMR data for minutumamide B2 (**4**) in DMSO-$d_6$

| position | $^{13}C$ (type) | $^{1}H$, m ($J$ Hz) | $^{1}H$-$^{1}H$ COSY | HMBC ($^{1}$**H** to $^{13}C$) |
|---|---|---|---|---|
| **Dpba** | | | | |
| 1 | 165.2 (C) | | | Dpba-H2, H3, Thr-H2, NH |
| 2 | 125.0 (CH) | 6.32, d (15.3) | Dpba-H3 | Dpba-H4 |
| 3 | 142.2 (CH) | 6.77, ddd (15.3, 7.1, 6.9) | Dpba-H2, H4 | Dpba-H4 |
| 4 | 37.5 ($CH_2$) | 3.49, brd (6.7) | Dpba-H3 | Dpba-H2, H3, H6, H10 |
| 5 | 138.8 (C) | | | Dpba-H3, H4, H7, H9 |
| 6,10 | 128.6 (CH) | 7.22, m | Dpba-H6, H7, H9, H10 | Dpba-H4, H6, H8, H10 |
| 7,9 | 128.7 (CH) | 7.32, t (7.6) | Dpba-H6, H7, H8, H9, H10 | Dpba-H7, H9 |
| 8 | 126.1 (CH) | 7.21, m | Dpba-H7, H9 | Dpba-H6, H10 |
| **Thr** | | | | |
| 1 | 166.5 (C) | | | Thr-H2, Hmp-H2, H5 |
| 2 | 53.9 (CH) | 5.08, dd (9.1, 2.6) | Thr-H3, NH | Thr-H4 |
| 3 | 68.9 (CH) | 5.32, m | Thr-H2, H4 | Thr-H4 |
| 4 | 16.3 ($CH_3$) | 1.11, d (6.2) | Thr-H3 | Thr-H3 |
| NH | | 8.29, d (8.8) | Thr-H2 | |
| **Hmp** | | | | |
| 1 | 168.6 (C) | | | Hmp-H2, Amopa-H4 |
| 2 | 65.3 (CH) | 4.55, d (5.7) | Hmp-H3 | Hmp-H5 |
| 3 | 72.2 (CH) | 4.38, m | Hmp-H2, H4, OH | Hmp-H2, H5, H6 |
| 4 | 39.1 (CH) | 2.10, m | Hmp-H3, H5, H6 | Hmp-H5, H6 |
| 5 | 51.6 ($CH_2$) | 3.08, t (10.1) | Hmp-H4, H5 | Hmp-H2, H3, H4, H6 |
| | | 3.94, m | Hmp-H4, H5 | |
| 6 | 10.3 ($CH_3$) | 0.95, d (6.6) | Hmp-H4 | Hmp-H4, H5 |
| OH | | 5.87, br | Hmp-H3 | |
| **Amopa** | | | | |
| 1 | 168.3 (C) | | | Amopa-H12, Tyr-H2 |
| 2 | 46.0 (CH) | 4.06, q (6.9) | Amopa-H12 | Amopa-H12 |
| 3 | 204.5 (C) | | | Amopa-H2, H5, H12 |
| 4 | 58.9 (CH) | 3.93, m | Amopa-H5, NH | Amopa-H5 |
| 5 | 34.2 ($CH_2$) | 2.77, m | Amopa-H4, H5 | Amopa-H7, H11 |
| | | 3.06, dd (13.9, 3.6) | Amopa-H4, H5 | |
| 6 | 138.9 (C) | | | Amopa-H5, H8, H10 |
| 7,11 | 130.1 (CH) | 7.42, d (7.4) | Amopa-H7, H8, H10, H11 | Amopa-H5, H7, H8, H10, H11 |
| 8,10 | 126.3 (CH) | 7.26, t (7.4) | Amopa-H7, H8, H9, H10, H11 | Amopa-H8, H10 |
| 9 | 126.1 (CH) | 7.20, m | Amopa-H8, H10 | Amopa-H7, H11 |
| 12 | 14.0 ($CH_3$) | 1.00, d (6.9) | Amopa-H2 | Amopa-H2 |
| NH | | 8.95, br | Amopa-H4 | |
| **Tyr** | | | | |
| 1 | 171.0 (C) | | | Tyr-H2, H3, Asp-H2 |
| 2 | 55.1 (CH) | 4.28, ddd (10.5, 9.2, 3.2) | Tyr-H3, NH | Tyr-H3 |
| 3 | 37.0 ($CH_2$) | 2.40, dd (14.0, 10.5) | Tyr-H2, H3 | Tyr-H2, H5, H9 |
| | | 3.00, dd (14.0, 3.2) | Tyr-H2, H3 | |
| 4 | 128.5 (C) | | | Tyr-H2, H6, H8 |
| 5,9 | 129.7 (CH) | 7.02, d (8.4) | Tyr-H5, H6, H8, H9 | Tyr-H3, H5, H9 |
| 6,8 | 115.0 (CH) | 6.62, d (8.4) | Tyr-H5, H6, H8, H9 | Tyr-H6, H8, OH |
| 10 | 155.6 (C) | | | Tyr-H5, H6, H8, H9, OH |
| OH | | 9.17, s | | |
| NH | | 7.13, br | Tyr-H2 | |
| **Asp** | | | | |
| 1 | 171. 8 (C) | | | Asp-H2, H3 |
| 2 | 48.0 (CH) | 4.64, ddd (11.0, 8.3, 3.9) | Asp-H3, NH | Asp-H3 |
| 3 | 34.7 ($CH_2$) | 2.63, dd (18.0, 11.0) | Asp-H2, H3 | Asp-H2 |
| | | 2.76, dd (18.0, 3.9) | Asp-H2, H3 | |
| 4 | 169.4 (C) | | | Asp-H2, H3, Thr-H3 |
| NH | | 7.26, m | Asp-H2 | |

**Table S5.** Temperature coefficients ($\Delta\delta/\Delta T$) of amide proton signals ($^{1}$H NMR) of minutumamides (**1**–**4**)

| | Minutumamide A1 (**1**) | Minutumamide A2 (**2**) | Minutumamide B1 (**3**) | Minutumamide B2 (**4**) |
|---|---|---|---|---|
| **Thr** | | | | |
| 333 K (ppm) | 7.69 | 7.87 | 7.80 | 8.09 |
| 293 K (ppm) | 8.00 | 8.12 | 8.10 | 8.37 |
| $\Delta\delta/\Delta T$ (ppb/K) | –7.75 | –6.25 | –7.50 | –7.00 |
| **Amopa** | | | | |
| 333 K (ppm) | 8.64 | 8.80 | 8.77 | 8.81 |
| 293 K (ppm) | 8.82 | 8.97 | 8.92 | 8.98 |
| $\Delta\delta/\Delta T$ (ppb/K) | –4.50 | –4.25 | –3.75 | –4.25 |
| **Tyr** | | | | |
| 333 K (ppm) | 7.37 | 7.11 | 7.19 | 7.12 |
| 293 K (ppm) | 7.59 | 7.16 | 7.39 | 7.15 |
| $\Delta\delta/\Delta T$ (ppb/K) | –5.50 | –0.25 | –5.00 | –0.75 |
| **Asp** | | | | |
| 333 K (ppm) | 8.37 | 7.258 | 8.40 | 7.263 |
| 293 K (ppm) | 8.44 | 7.262 | 8.45 | 7.266 |
| $\Delta\delta/\Delta T$ (ppb/K) | –1.75 | –0.10 | –1.25 | –0.75 |

**Table S6.** $^{1}H$ (600 MHz) and $^{13}C$ (150 MHz) NMR data for nostopeptolide KVJ3 (**7**) in DMSO-$d_6$

| position | $^{13}C$ (type) | $^{1}H$, m ($J$ Hz) | $^{1}H$-$^{1}H$ COSY | HMBC ($^{1}$**H** to $^{13}C$) |
|---|---|---|---|---|
| **Ha** | | | | |
| 1 | 172.9 (C) | | | Ha-H2, H3, Thr-H2, NH |
| 2 | 34.9 ($CH_2$) | 2.20, m | Ha-H3 | Ha-H3, H4 |
| | | 2.28, m | Ha-H3 | |
| 3 | 25.2 ($CH_2$) | 1.50, m | Ha-H2, H4 | Ha-H2, H4, H5 |
| 4 | 31.0 ($CH_2$) | 1.23, m | Ha-H3, H5 | Ha-H2, H3, H5, H6 |
| 5 | 22.0 ($CH_2$) | 1.27, m | Ha-H4, H6 | Ha-H3, H4, H6 |
| 6 | 13.9 ($CH_3$) | 0.85, t (6.8) | Ha-H5 | Ha-H4, H5 |
| **Thr** | | | | |
| 1 | 166.4 (C) | | | Thr-H2, Hmp1-H2 |
| 2 | 53.5 (CH) | 4.92, dd (8.6, 2.0) | Thr-H3, NH | Thr-H4 |
| 3 | 69.1 (CH) | 5.32, m | Thr-H2, Thr-H4 | Thr-H4 |
| 4 | 16.9 ($CH_3$) | 1.13, d (6.3) | Thr-H3 | |
| NH | | 8.40, d (8.6) | Thr-H2 | |
| **Hmp1** | | | | |
| 1 | 167.4 (C) | | | Hmp1-H2, Amopa-NH |
| 2 | 64.6 (CH) | 4.51, d (6.1) | Hmp1-H3 | Hmp1-OH |
| 3 | 71.7 (CH) | 4.36, m | Hmp1-H2, H4, OH | Hmp1-H5, H6, OH |
| 4 | 39.2 (CH) | 2.14, m | Hmp1-H3, H5, H6 | Hmp1-H6 |
| 5 | 51.2 ($CH_2$) | 3.19, t (10.2) | Hmp1-H4, H5 | Hmp1-H3, H6 |
| | | 3.81, m | Hmp1-H4, H5 | |
| 6 | 10.7 ($CH_3$) | 0.98, d (6.7) | Hmp1-H4 | Hmp1-H5 |
| OH | | 5.52, d (4.1) | Hmp1-H3 | |
| **Amopa** | | | | |
| 1 | 167.9 (C) | | | Amopa-H2, H12, Ser-NH |
| 2 | 47.7 (CH) | 4.19, q (6.4) | Amopa-H12 | Amopa-H12 |
| 3 | 202.7 (C) | | | Amopa-H2, H4, H5, H12 |
| 4 | 54.5 (CH) | 5.48, br | Amopa-H5, NH | Amopa-H5 |
| 5 | 35.2 ($CH_2$) | 2.69, m | Amopa-H4, H5 | |
| | | 2.84, dd (14.3, 7.5) | Amopa-H4, H5 | |
| 6 | 137.7 (C) | | | Amopa-H5, H8, H11 |
| 7,11 | 129.6 (CH) | 7.26, d (7.3) | Amopa-H7, H8, H10, H11 | Amopa-H5, H7, H11, H9 |
| 8,10 | 127.8 (CH) | 7.16, t (7.3) | Amopa-H7, H8, H9, H10, H11 | Amopa-H8, H10 |
| 9 | 125.8 (CH) | 7.10, t (7.3) | Amopa-H8, H10 | Amopa-H7, H11 |
| 12 | 11.4 ($CH_3$) | 0.95, d (6.4) | Amopa-H2 | Amopa-H2 |
| NH | | 8.54, d (8.3) | Amopa-H4 | |
| **Ser** | | | | |
| 1 | 173.1 (C) | | | Ser-H3, Hmp2-H2 |
| 2 | 53.2 (CH) | 4.41, m | Ser-H3, NH | Ser-NH |
| 3 | 61.2 ($CH_2$) | 3.68, m | Ser-H2, H3 | Ser-NH |
| | | 3.78, m | Ser-H2, H3 | |
| OH | | 5.75, brs | Ser-H3 | |
| NH | | 7.59, d (5.8) | Ser-H2 | |
| **Hmp2** | | | | |
| 1 | 168.3 (C) | | | Hmp2-H2, Tyr-H2, NH |
| 2 | 68.5 (CH) | 4.26, d (4.6) | Hmp2-H3 | Hmp2-H5, OH |
| 3 | 71.4 (CH) | 4.12, m | Hmp2-H2, H4, OH | Hmp2-H2, H5, H6, OH |
| 4 | 38.4 (CH) | 2.10, m | Hmp2-H3, H5, H6 | Hmp2-H5, H6, OH |
| 5 | 52.1 ($CH_2$) | 3.35, m | Hmp2-H4, H5 | Hmp2-H6 |
| | | 3.94, brt (8.6) | Hmp2-H4, H5 | |
| 6 | 10.6 ($CH_3$) | 0.93, d (6.7) | Hmp2-H4 | |
| OH | | 5.06, d (5.4) | Hmp2-H3 | |
| **Tyr** | | | | |
| 1 | 172.4 (C) | | | Tyr-H2, H3, Gly-H2, NH |
| 2 | 54.9 (CH) | 4.23, m | Tyr-H3, NH | Tyr-H3 |
| 3 | 35.9 ($CH_2$) | 2.71, m | Tyr-H2, H3 | Tyr-H2, H5, H9 |
| | | 3.03, m | Tyr-H2, H3 | |
| 4 | 128.6 (C) | | | Tyr-H6, H8 |
| 5,9 | 129.7 (CH) | 6.99, d (8.5) | Tyr-H5, H6, H8, H9 | Tyr-H5, H6, H8, H9 |
| 6,8 | 114.8 (CH) | 6.60, d (8.5) | Tyr-H5, H6, H8, H9 | Tyr-H6, H8, OH |
| 7 | 155.7 (C) | | | Tyr-H6, H8, OH |
| OH | | 9.10, s | | |
| NH | | 7.44, d (8.1) | Tyr-H2 | |
| **Gly** | | | | |
| 1 | 168.9 (C) | | | Gly-H2, Thr-H3 |
| 2 | 40.1 ($CH_2$) | 3.59, m | Gly-H2, NH | Gly-NH |
| | | 3.76, m | Gly-H2, NH | |
| NH | | 7.93, t (6.1) | | |

## 4. Supplemental Figures

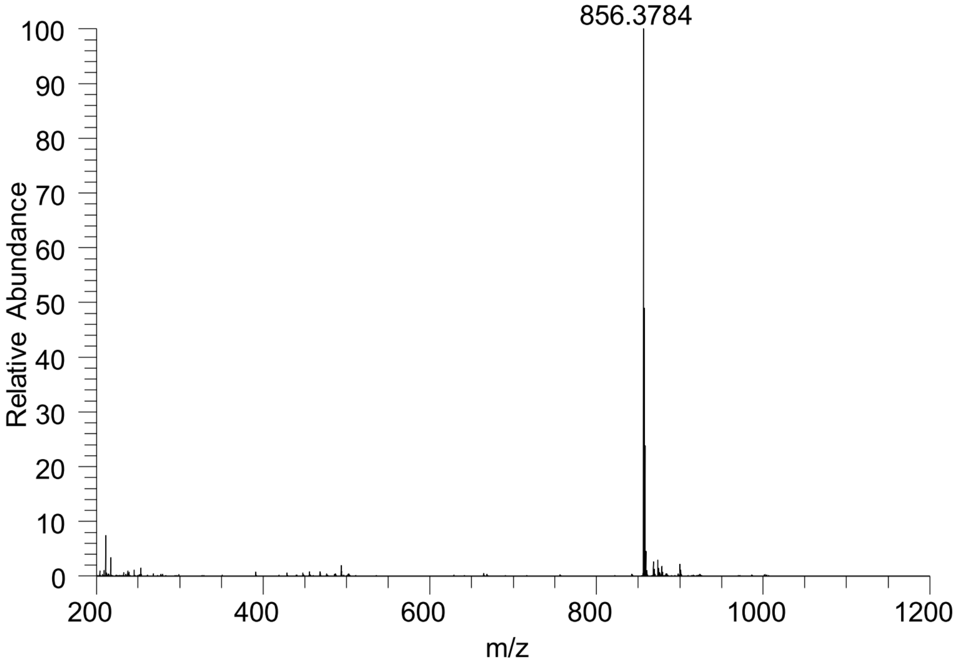


**Figure S1.** High-resolution mass spectrum in positive ion mode of minutumamide A1 (**1**).

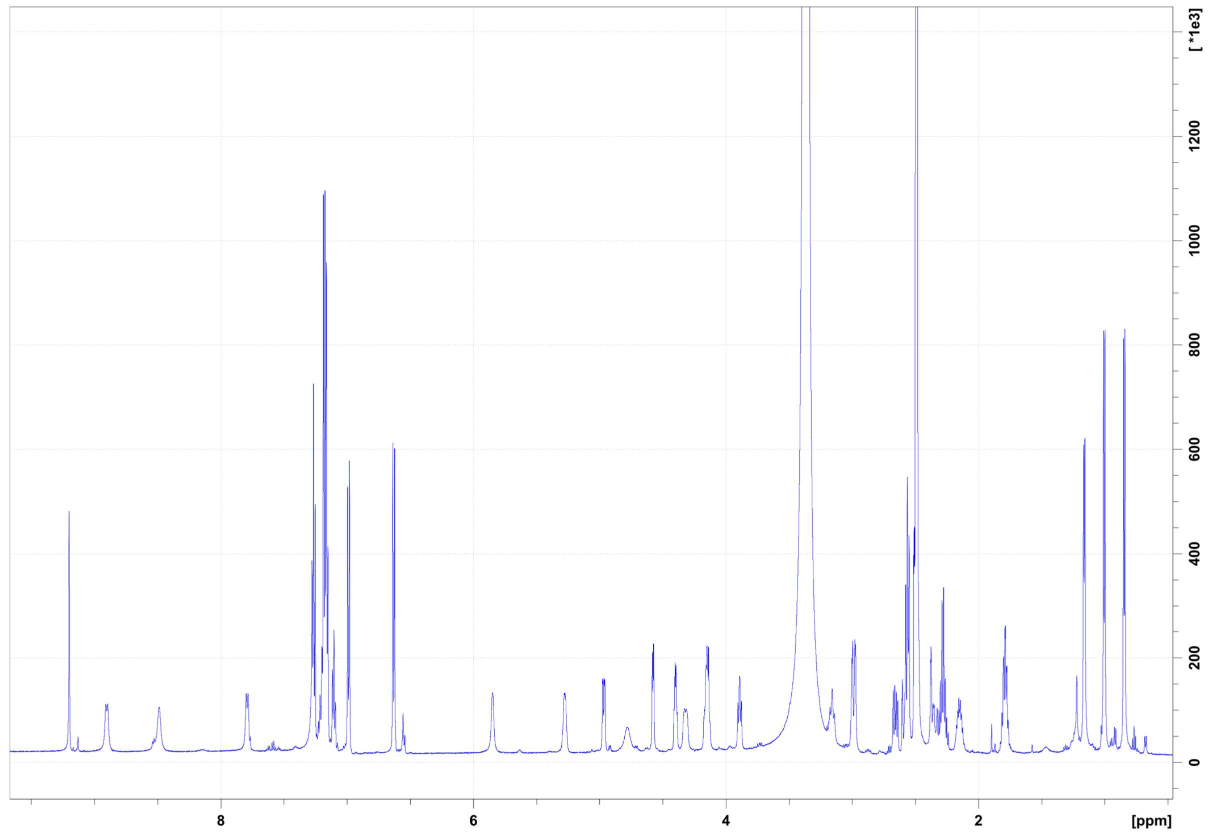


**Figure S2.** $^{1}H$ NMR spectrum of minutumamide A1 (**1**) in DMSO-$d_6$ (600 MHz).

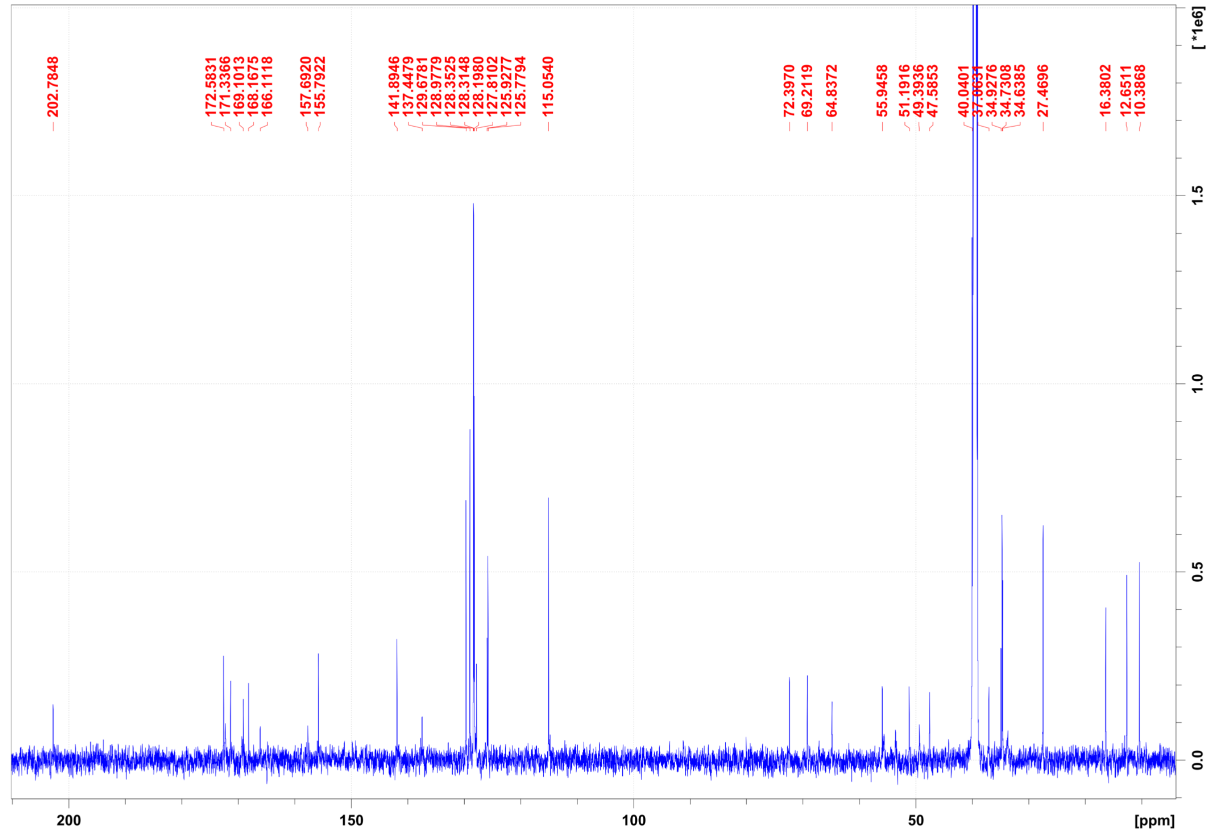


**Figure S3.** $^{13}C$ NMR spectrum of minutumamide A1 (**1**) in DMSO-$d_6$ (150 MHz).

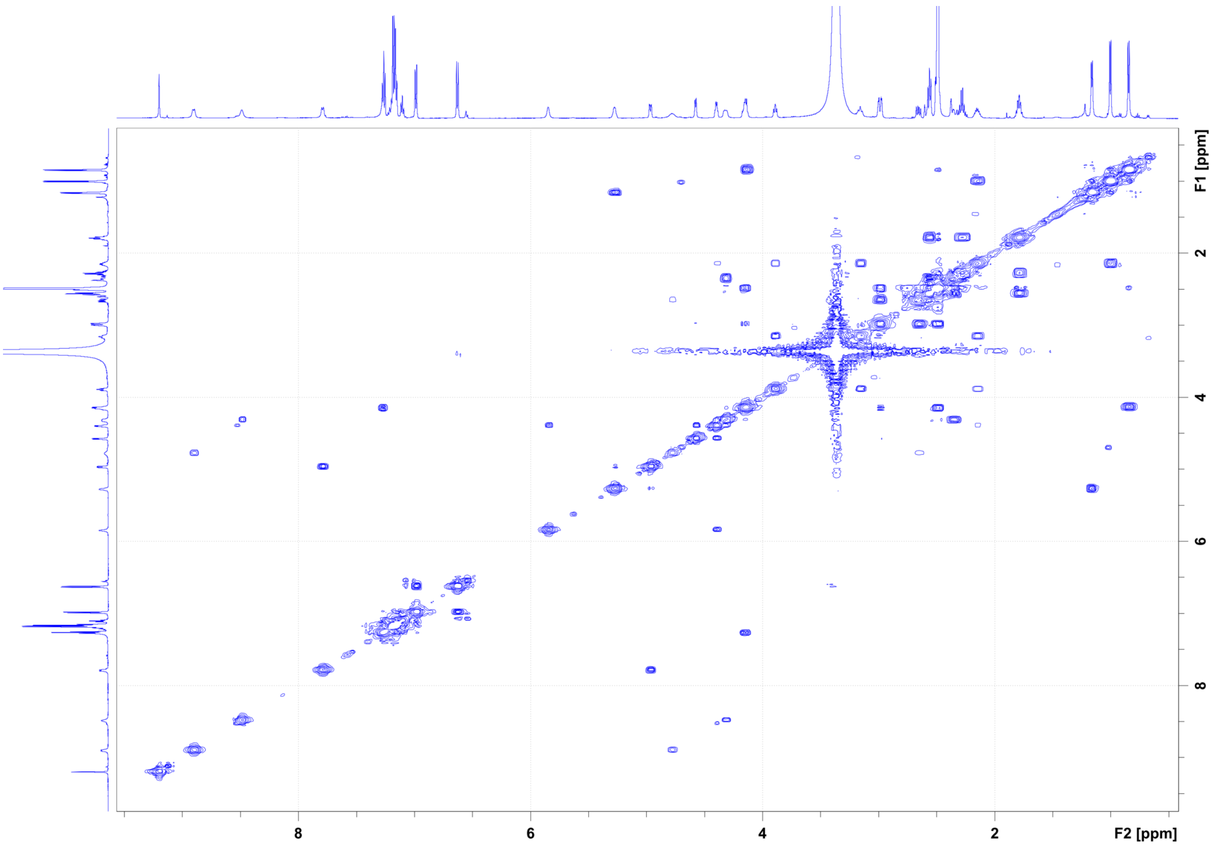

**Figure S4.** $^{1}H$-$^{1}H$ COSY spectrum of minutumamide A1 (**1**) in DMSO-$d_6$ (600 MHz).

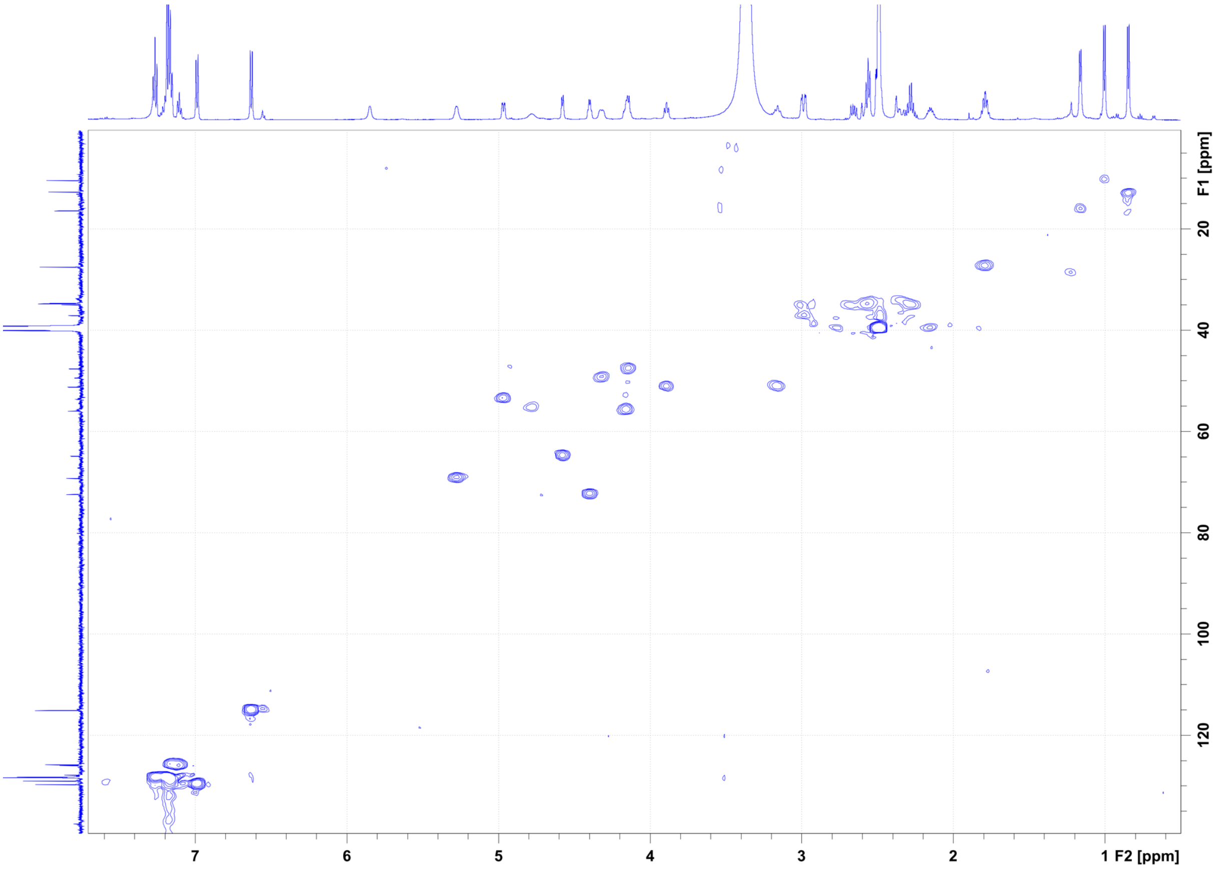

**Figure S5.** HSQC spectrum of minutumamide A1 (**1**) in DMSO-$d_6$ (600 MHz).

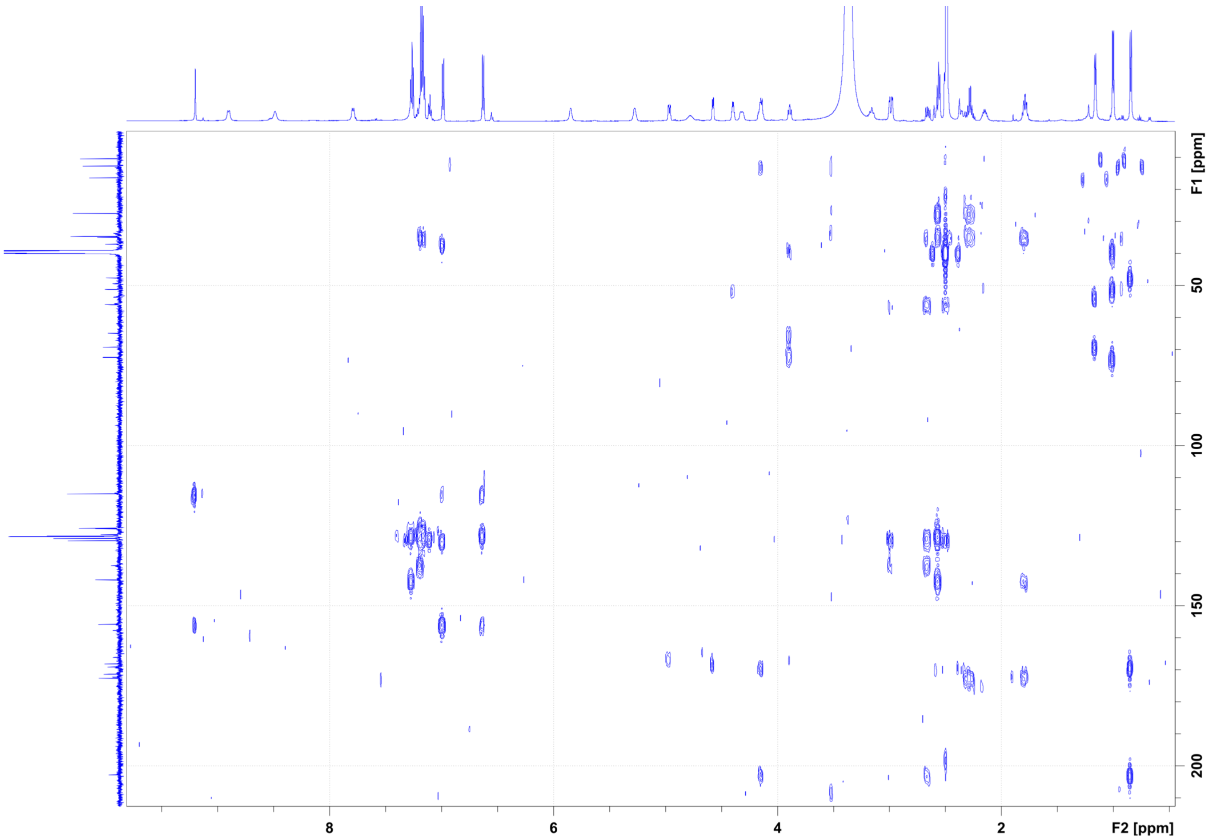


**Figure S6.** HMBC spectrum of minutumamide A1 (**1**) in DMSO-$d_6$ (600 MHz).

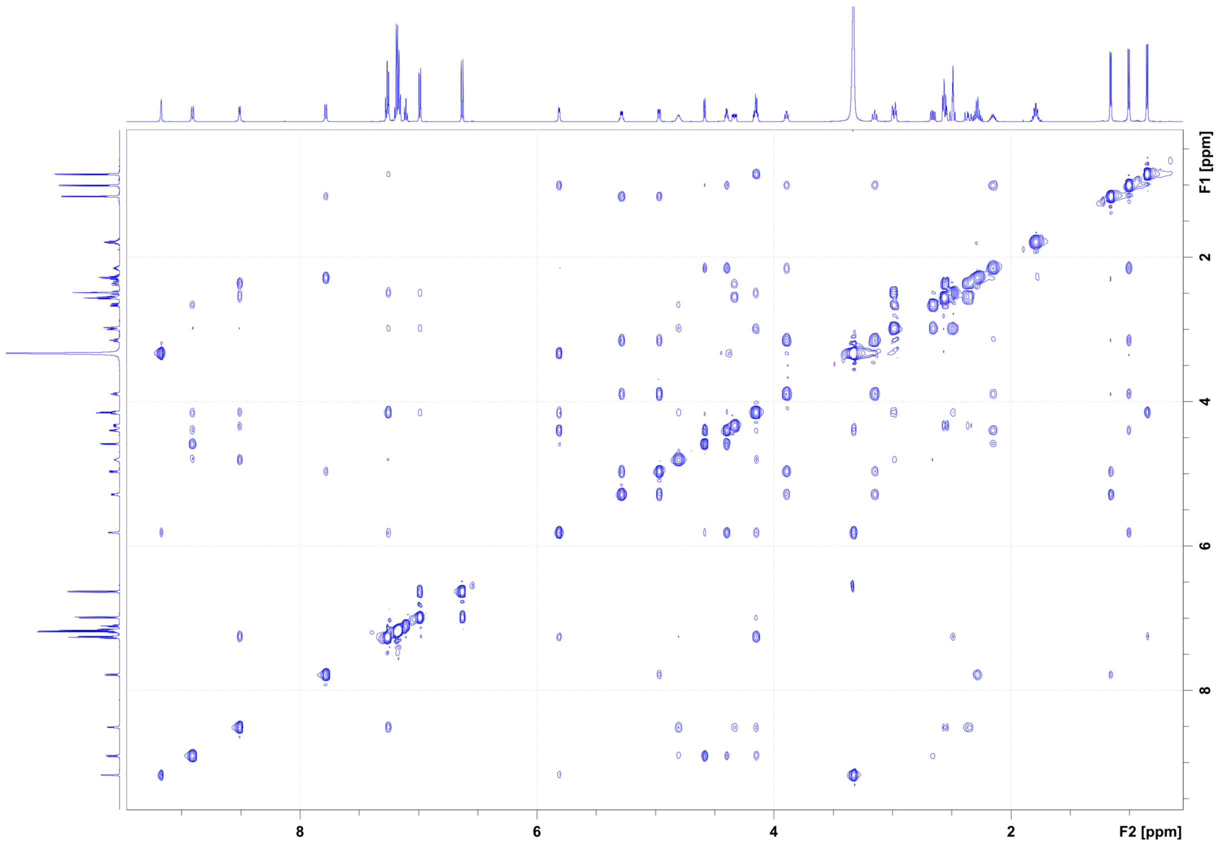


**Figure S7.** NOESY spectrum of minutumamide A1 (**1**) in DMSO-$d_6$ (600 MHz).

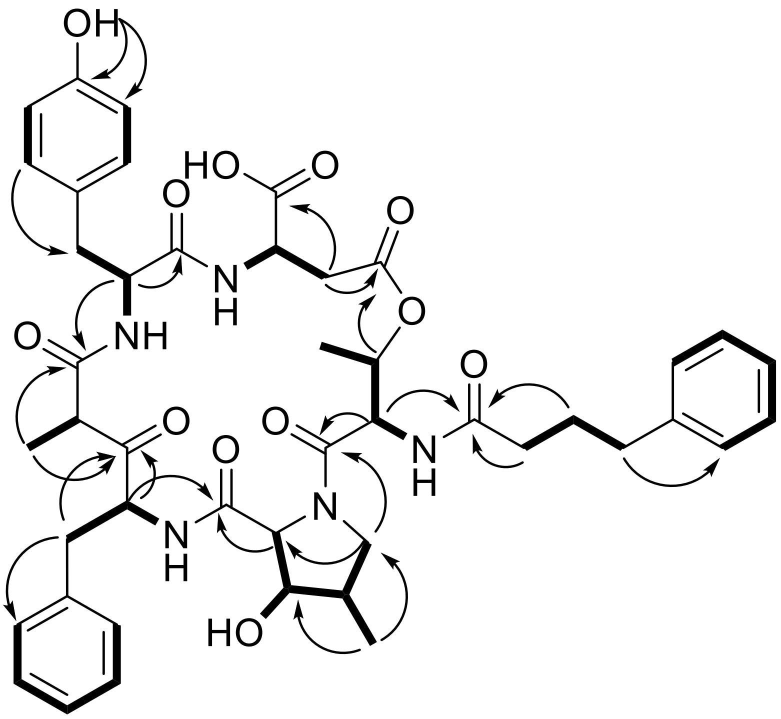


**Figure S8.** Observed and selected $^{1}H$-$^{1}H$ COSY (bold line) and HMBC (arrow) correlations of minutumamide A1 (**1**).

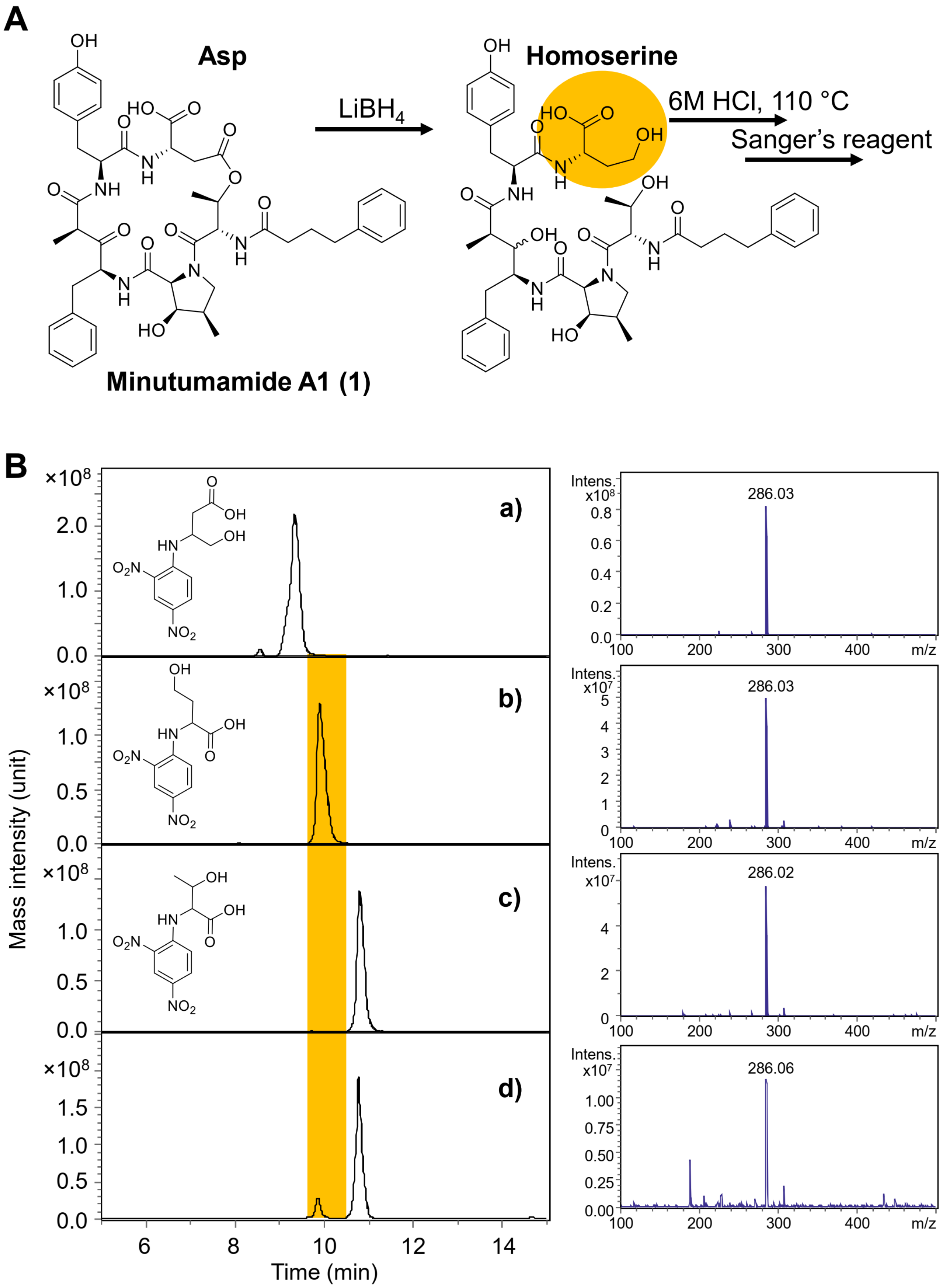


**Figure S9.** Determination of an ester bond position between Thr and Asp in minutumamide A1 (**1**). **A.** Scheme of chemical degradation of minutumamide A1 (**1**). **B.** The LC-MS profile (EIC, *m/z* 286.00 $[M+H]^+$) and observed ions of Sanger's reagent derivatives of β-homoserine **(a)**, homoserine **(b)**, threonine **(c)**, and acid hydrolysate of reduced minutumamide A1 (**1**) **(d)**.

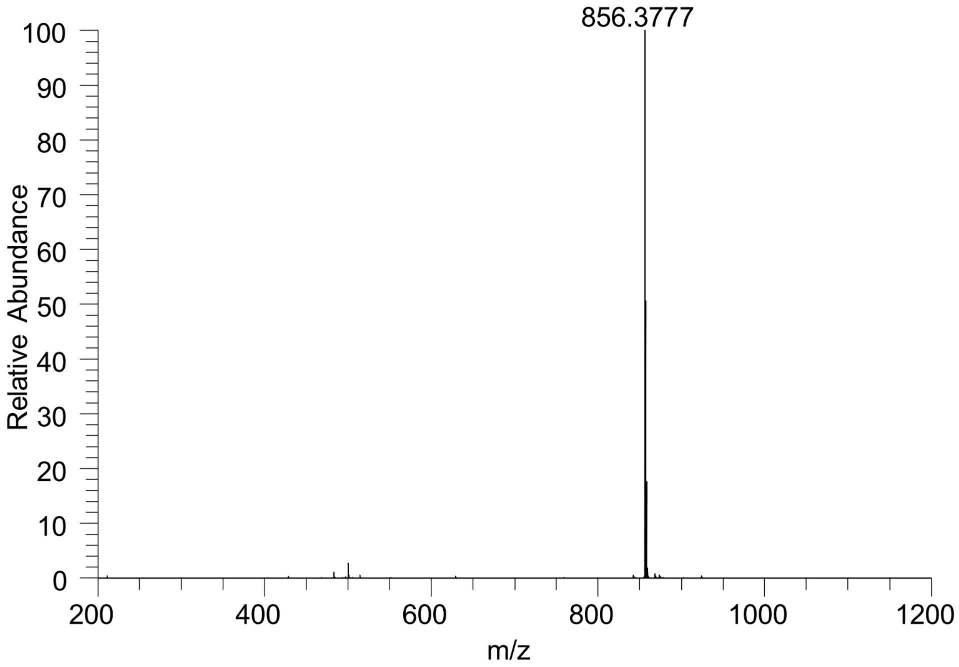


**Figure S10.** High-resolution mass spectrum in positive ion mode of minutumamide A2 (**2**).

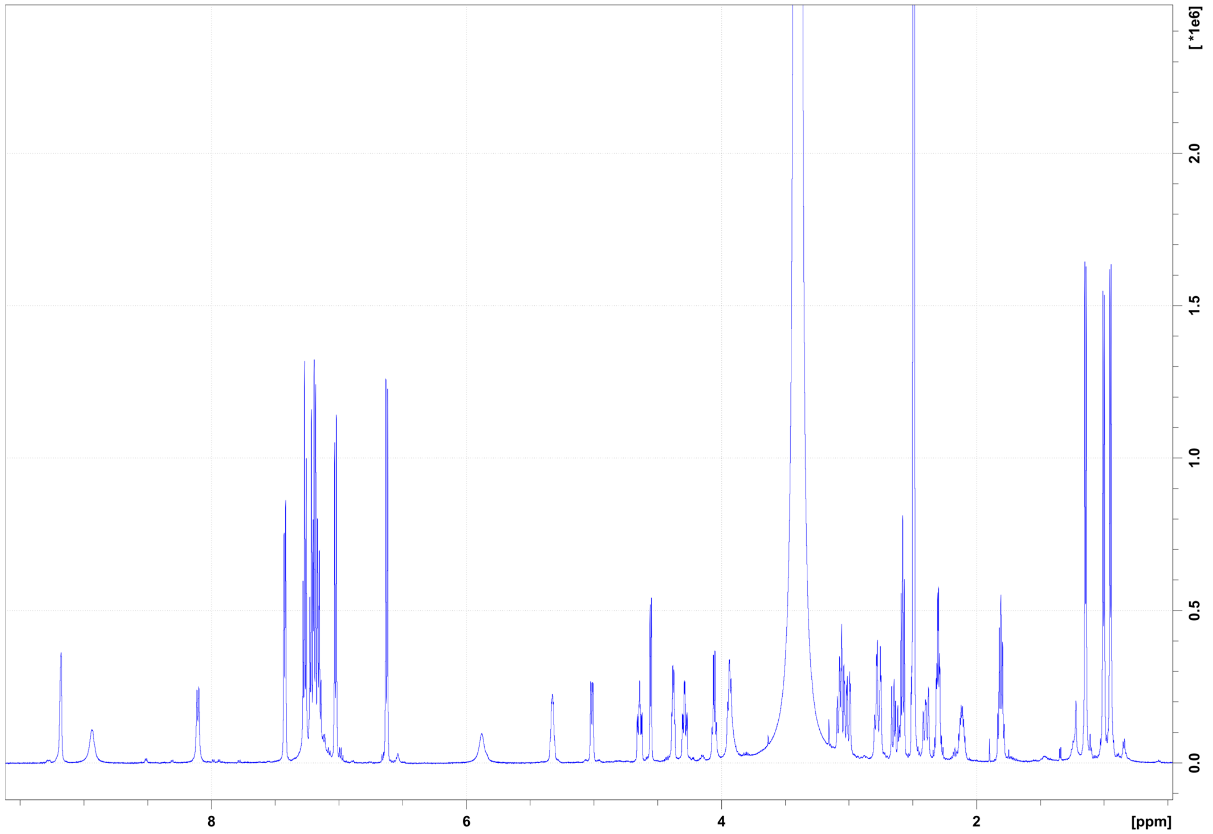

**Figure S11.** $^{1}$H NMR spectrum of minutumamide A2 (**2**) in DMSO-$d_6$ (600 MHz).

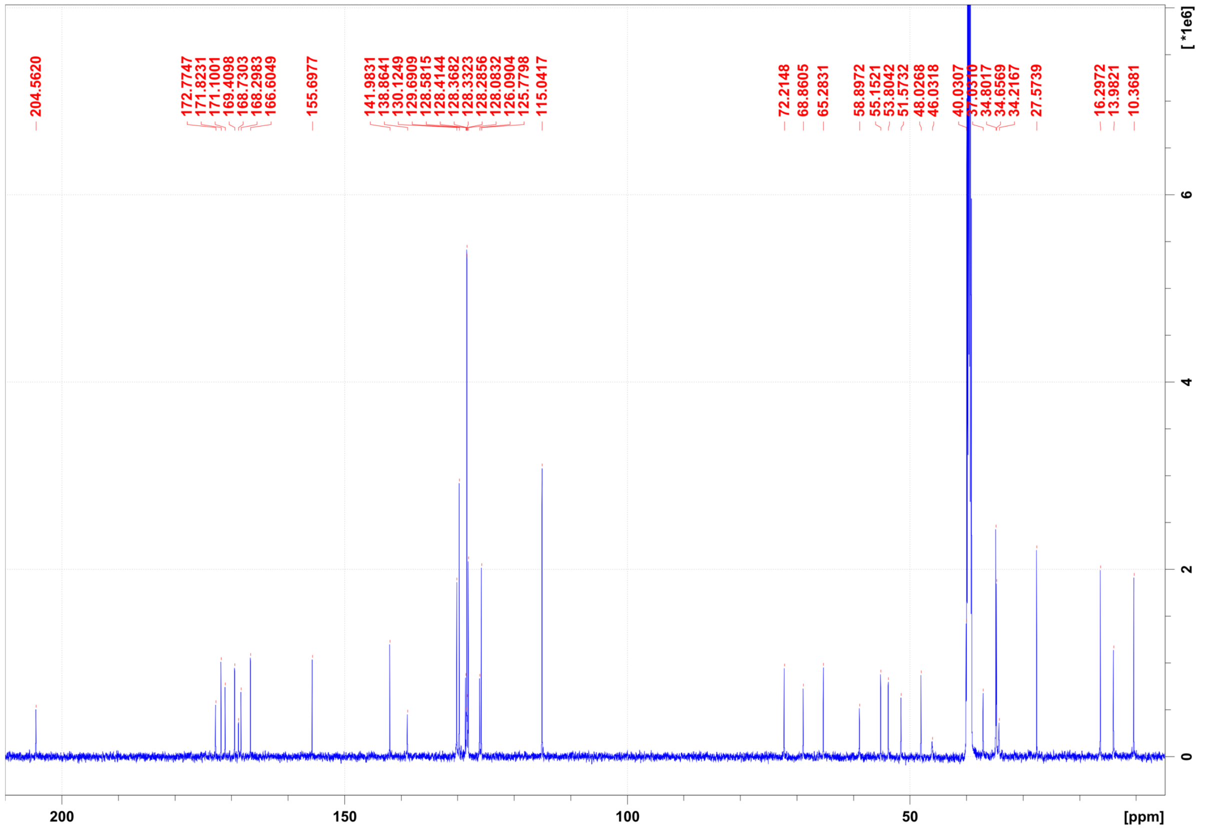


**Figure S12.** $^{13}$C NMR spectrum of minutumamide A2 (**2**) in DMSO-$d_6$ (150 MHz).

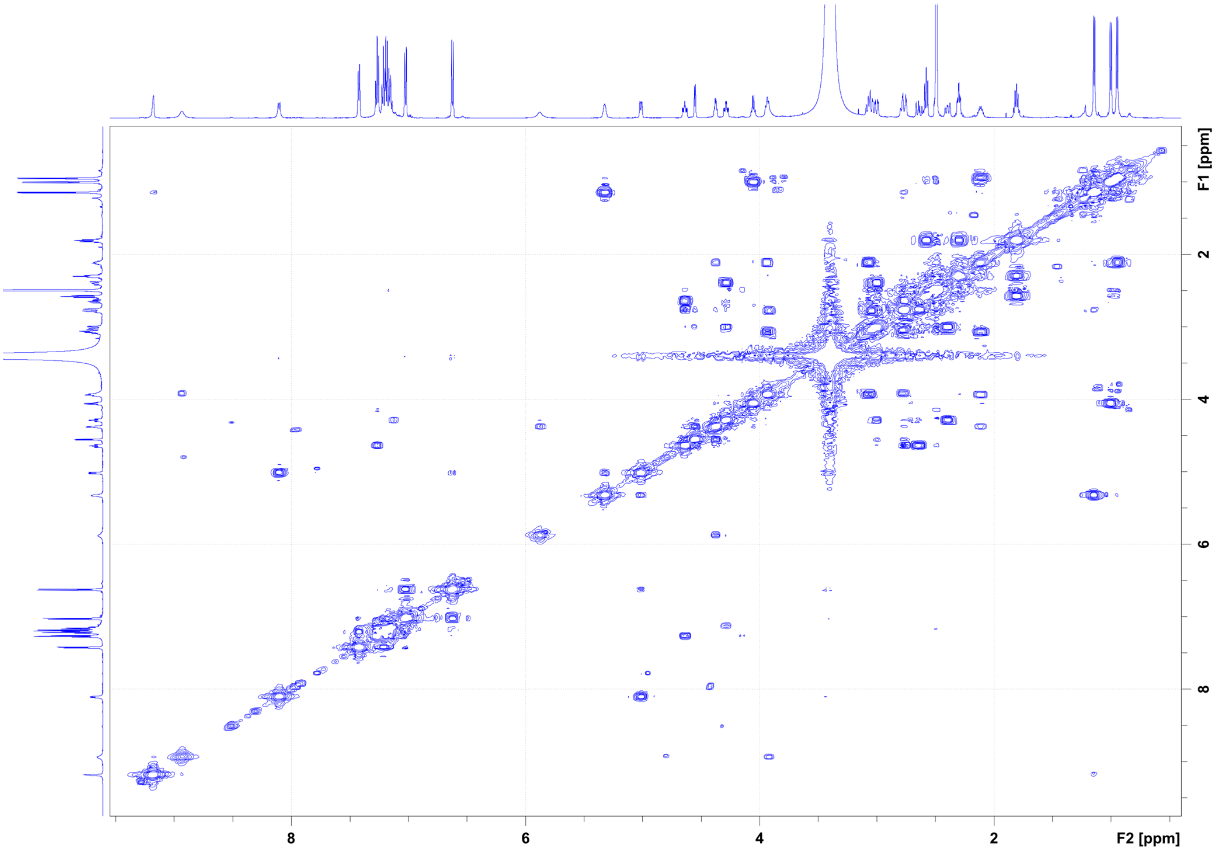


**Figure S13.** $^1$H-$^1$H COSY spectrum of minutumamide A2 (**2**) in DMSO-$d_6$ (600 MHz).

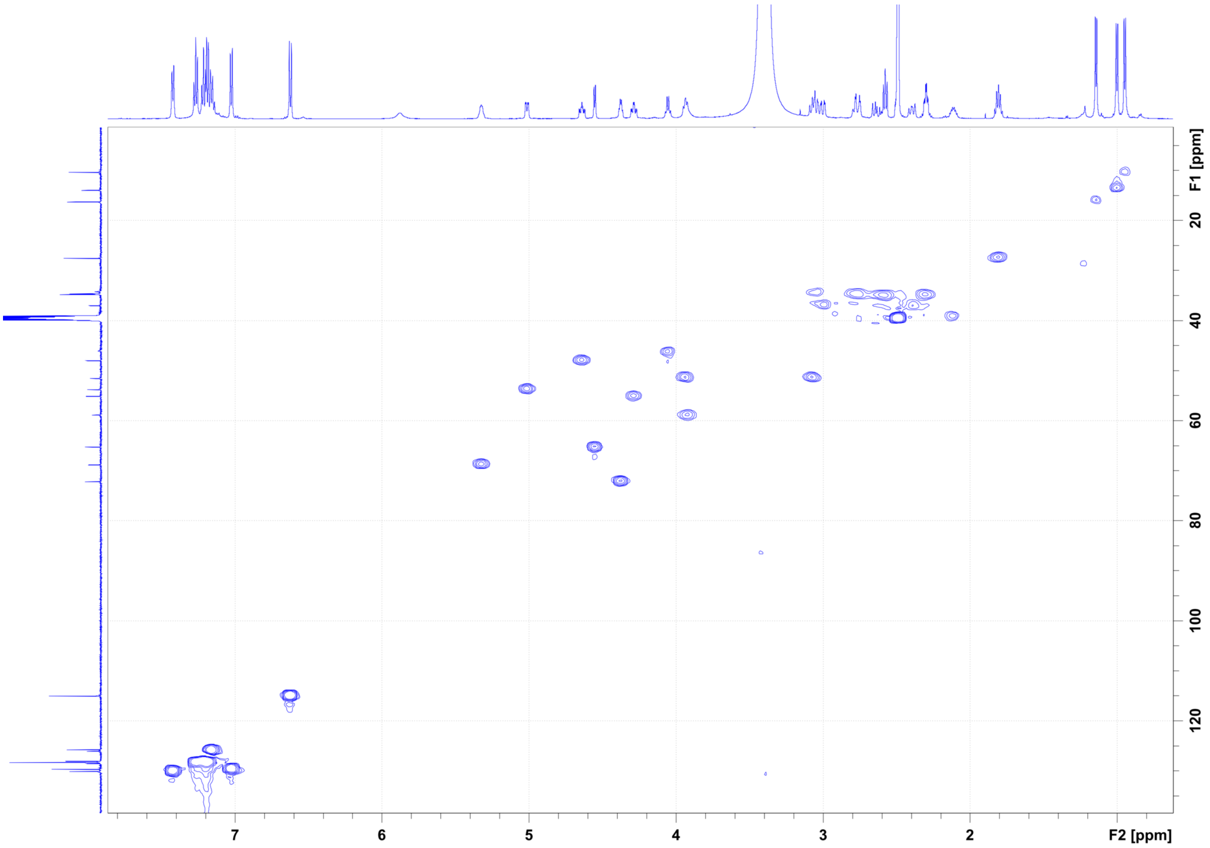


**Figure S14.** HSQC spectrum of minutumamide A2 (**2**) in DMSO-$d_6$ (600 MHz).

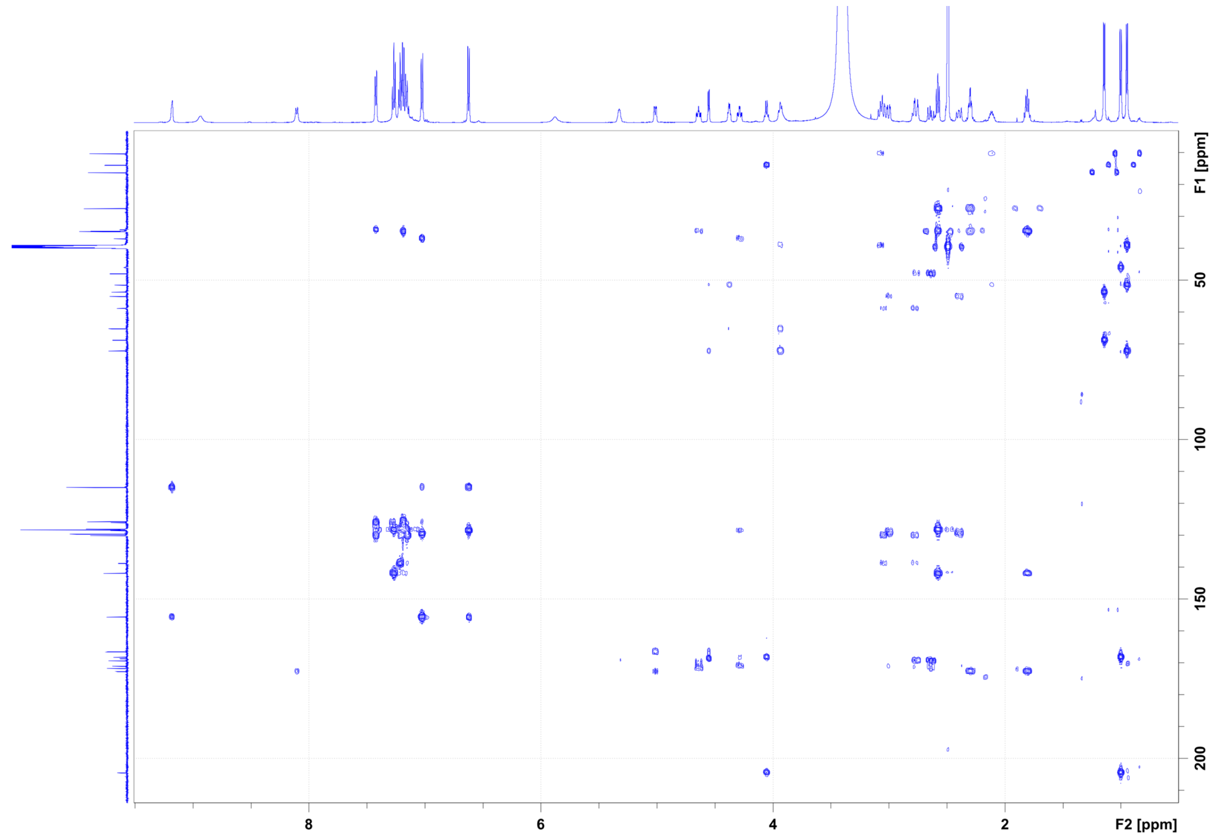


**Figure S15.** HMBC spectrum of minutumamide A2 (**2**) in DMSO-$d_6$ (600 MHz).

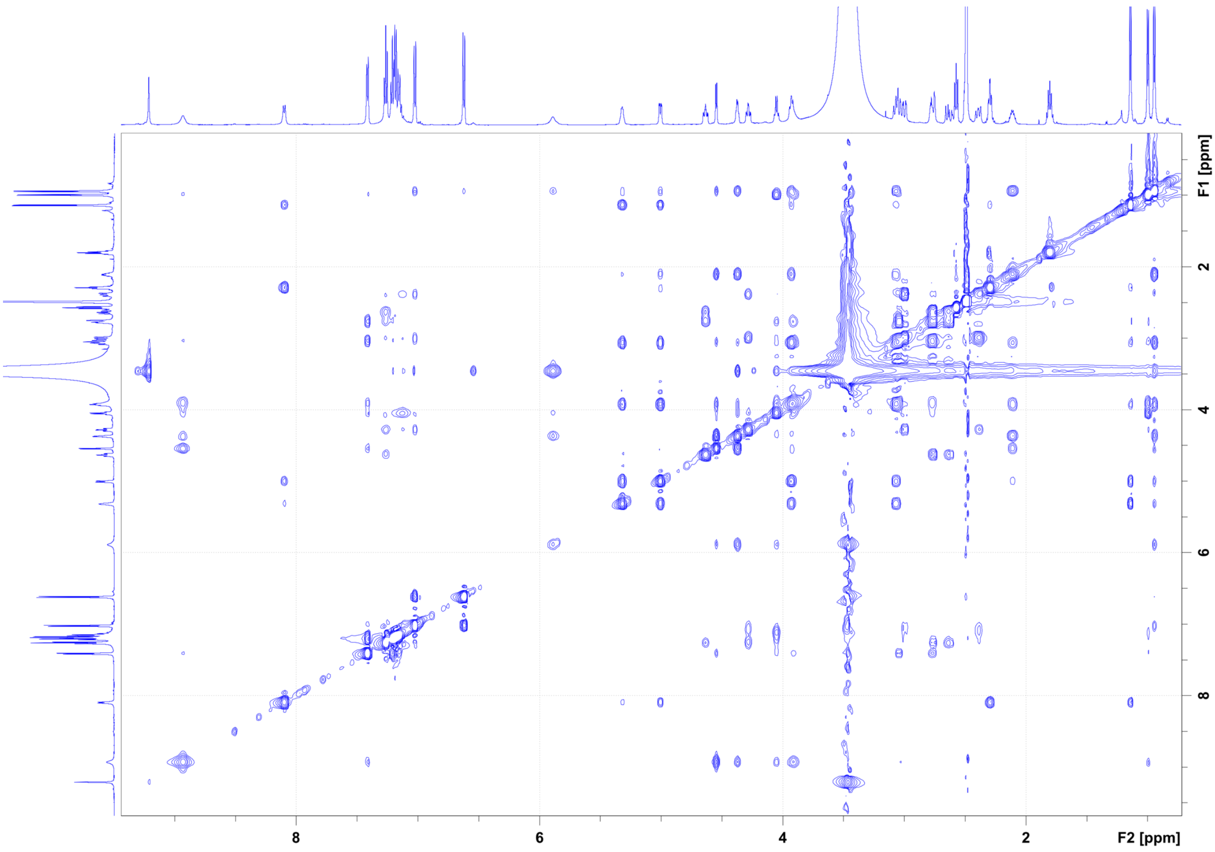


**Figure S16.** NOESY spectrum of minutumamide A2 (**2**) in DMSO-$d_6$ (600 MHz).

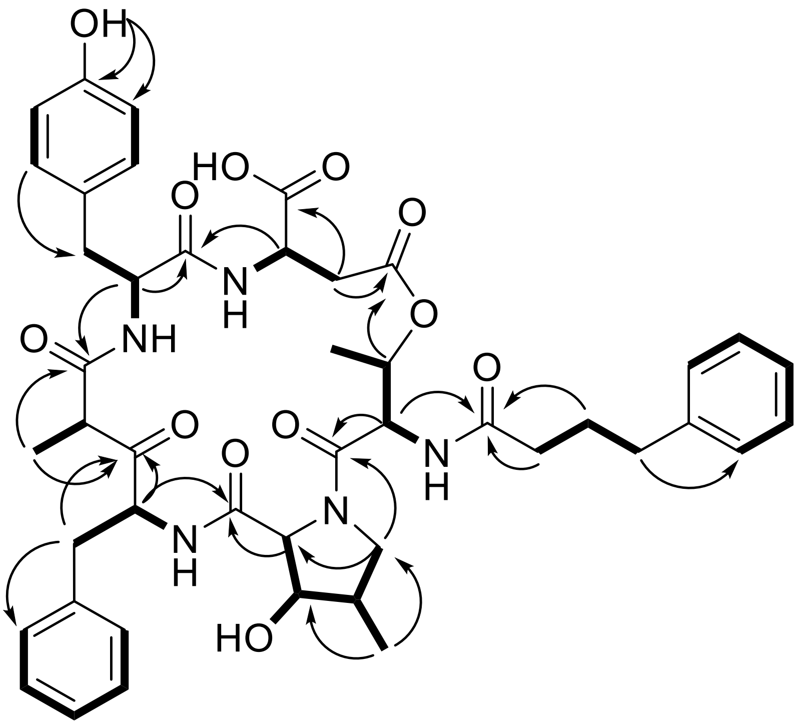


**Figure S17.** Observed and selected $^{1}H$-$^{1}H$ COSY (bold line) and HMBC (arrow) correlations of minutumamide A2 (**2**).

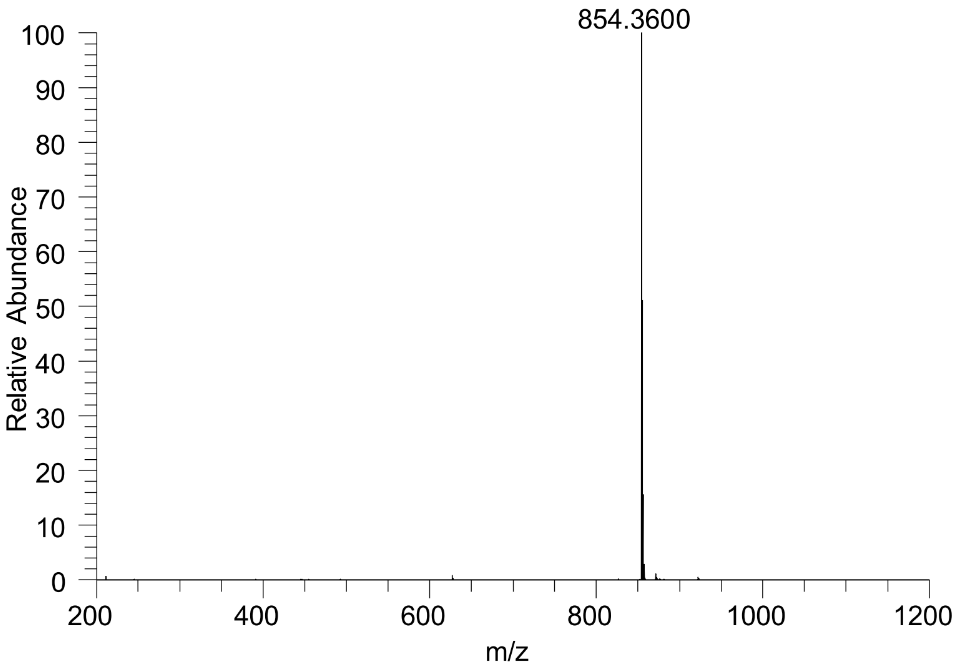

**Figure S18.** High-resolution mass spectrum in positive ion mode of minutumamide B1 (**3**).

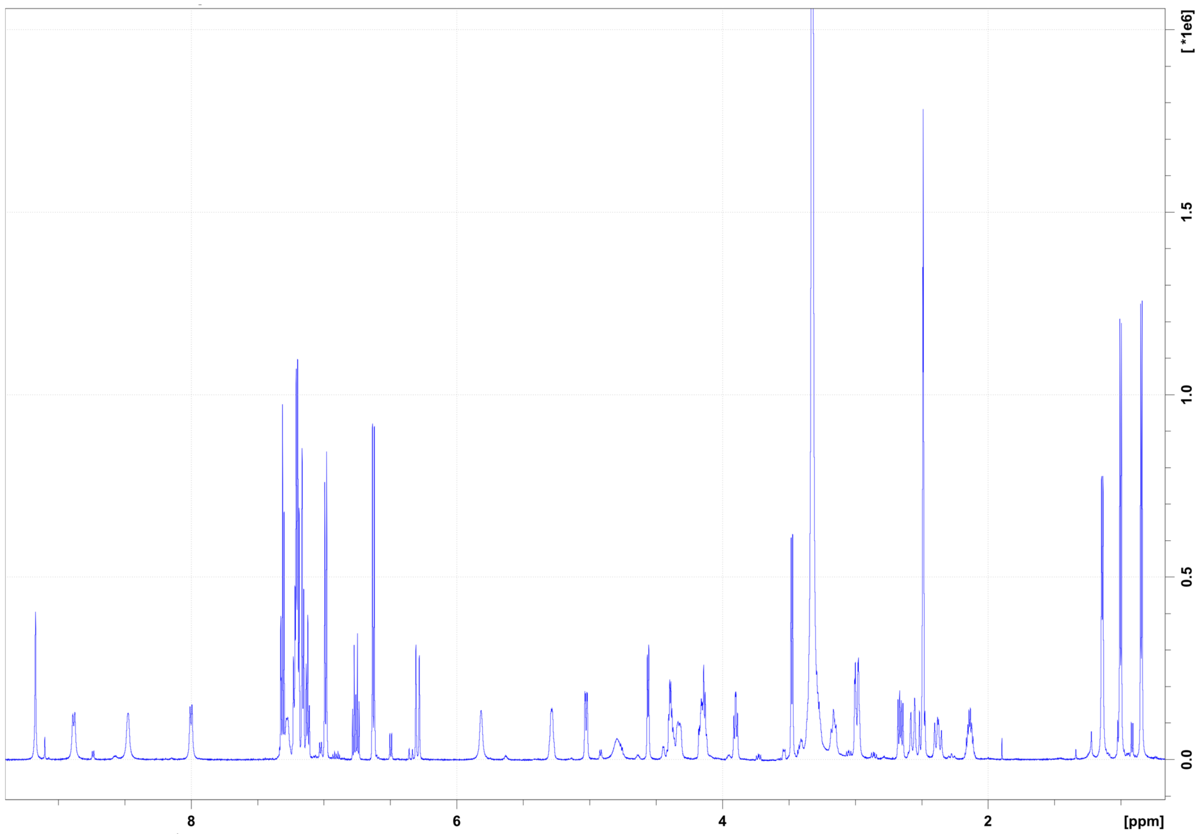


**Figure S19.** $^{1}$H NMR spectrum of minutumamide B1 (**3**) in DMSO-$d_6$ (600 MHz).

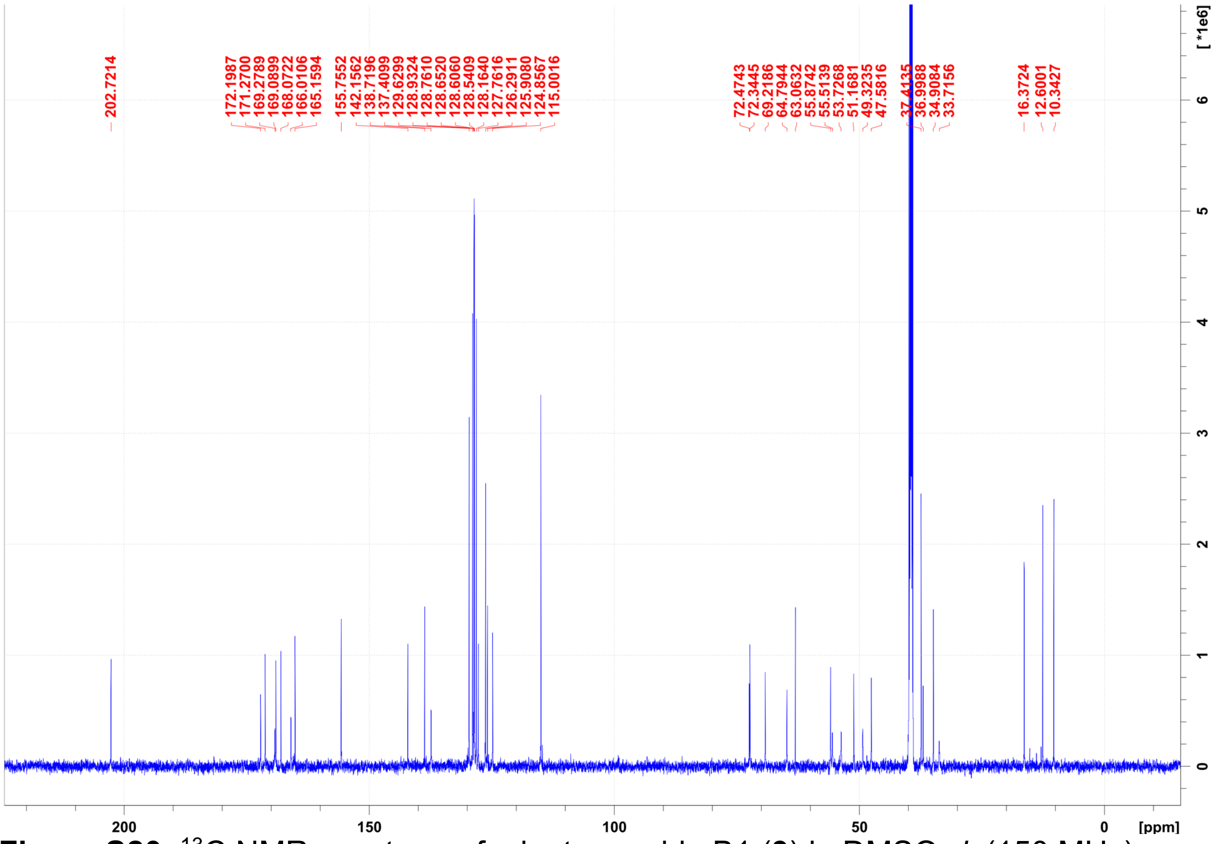


**Figure S20.** $^{13}$C NMR spectrum of minutumamide B1 (**3**) in DMSO-$d_6$ (150 MHz).

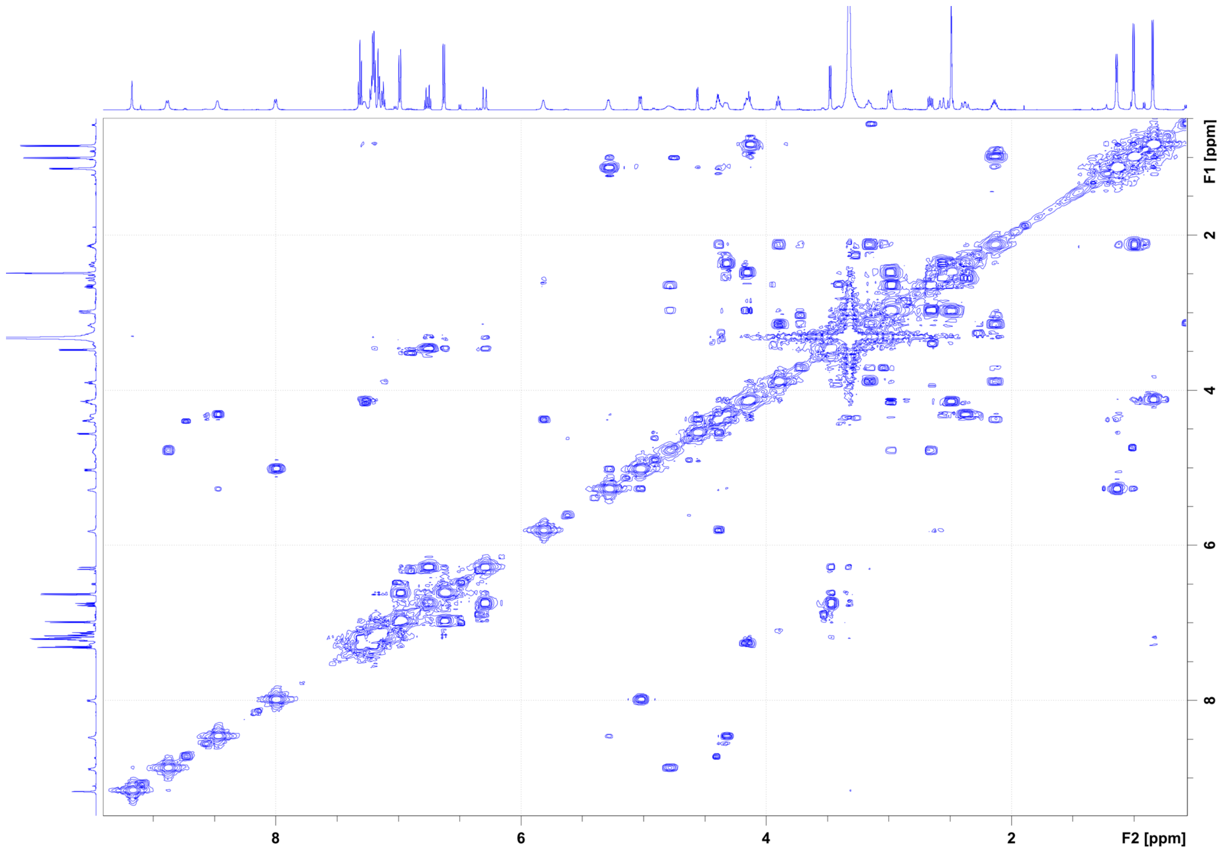


**Figure S21.** $^1$H-$^1$H COSY spectrum of minutumamide B1 (**3**) in DMSO-*d*$_6$ (600 MHz).

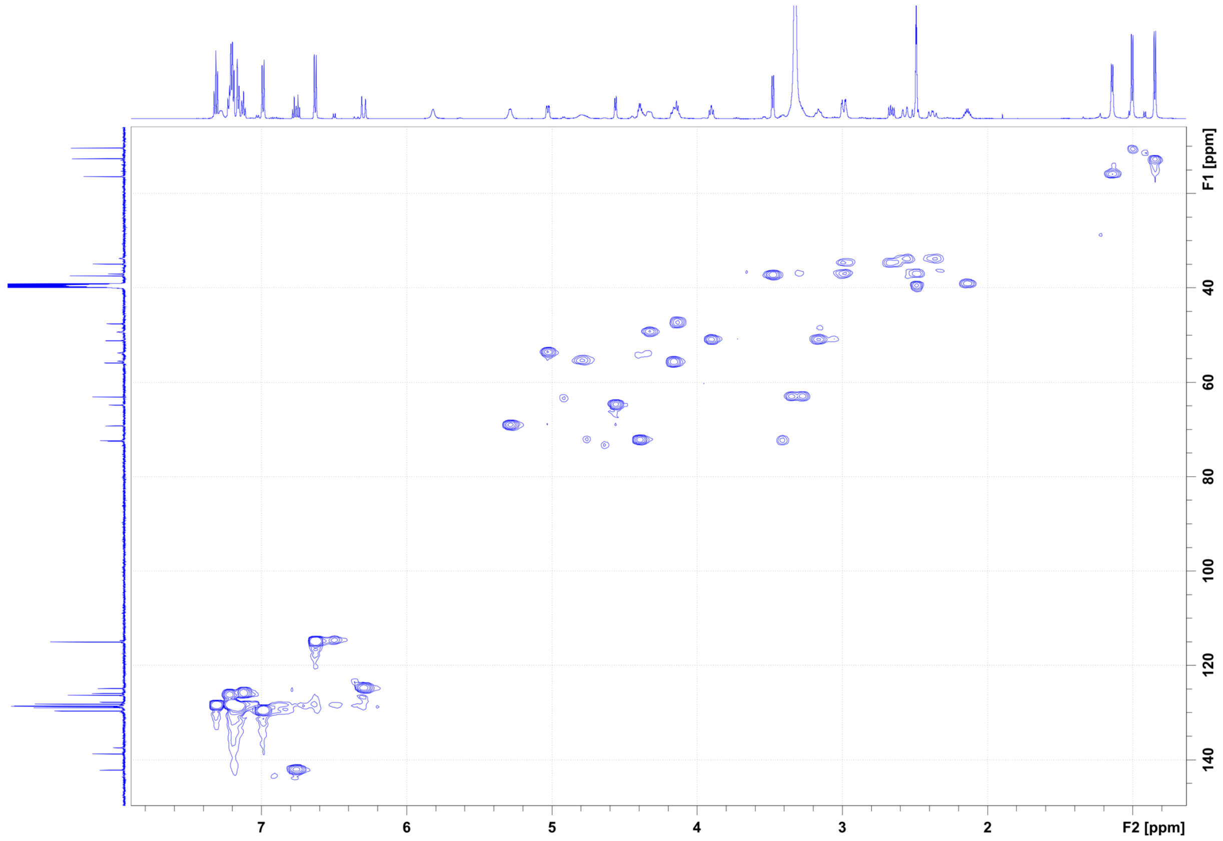


**Figure S22.** HSQC spectrum of minutumamide B1 (**3**) in DMSO-*d*$_6$ (600 MHz).

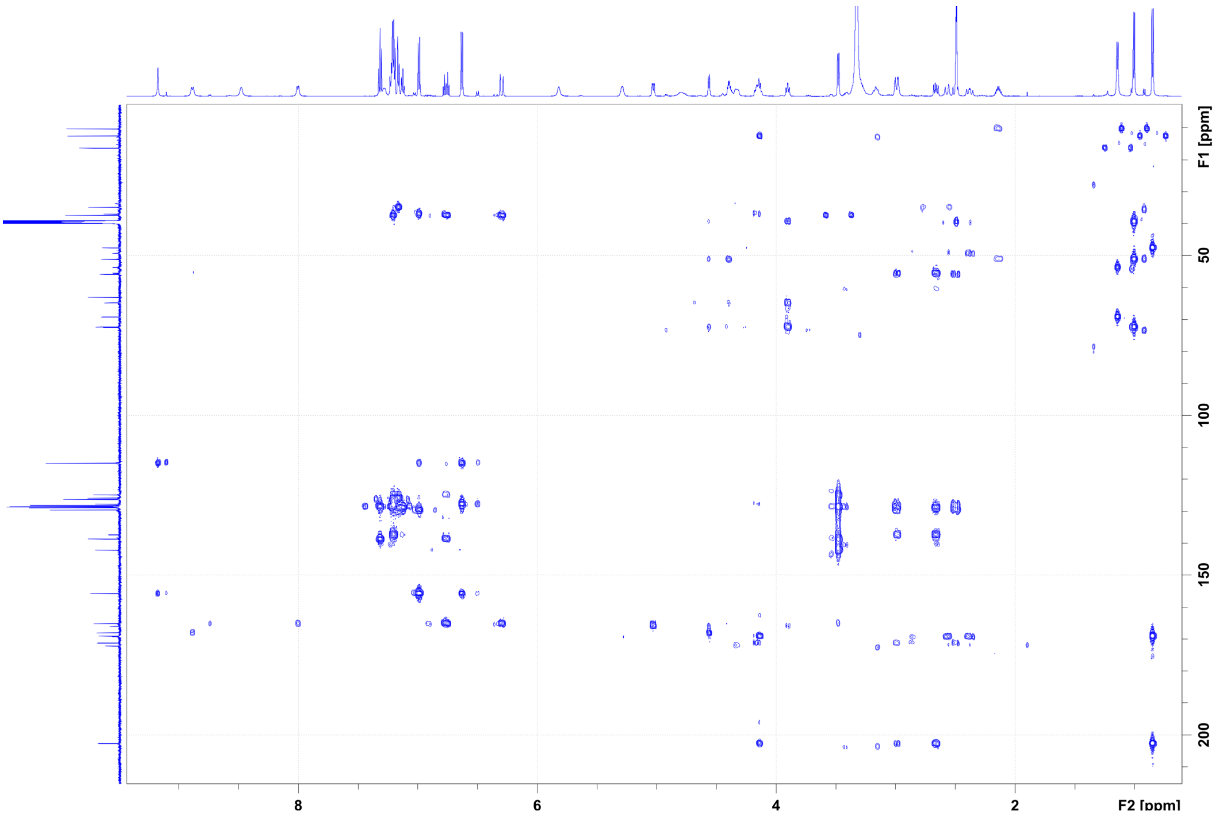


**Figure S23.** HMBC spectrum of minutumamide B1 (**3**) in DMSO-$d_6$ (600 MHz).

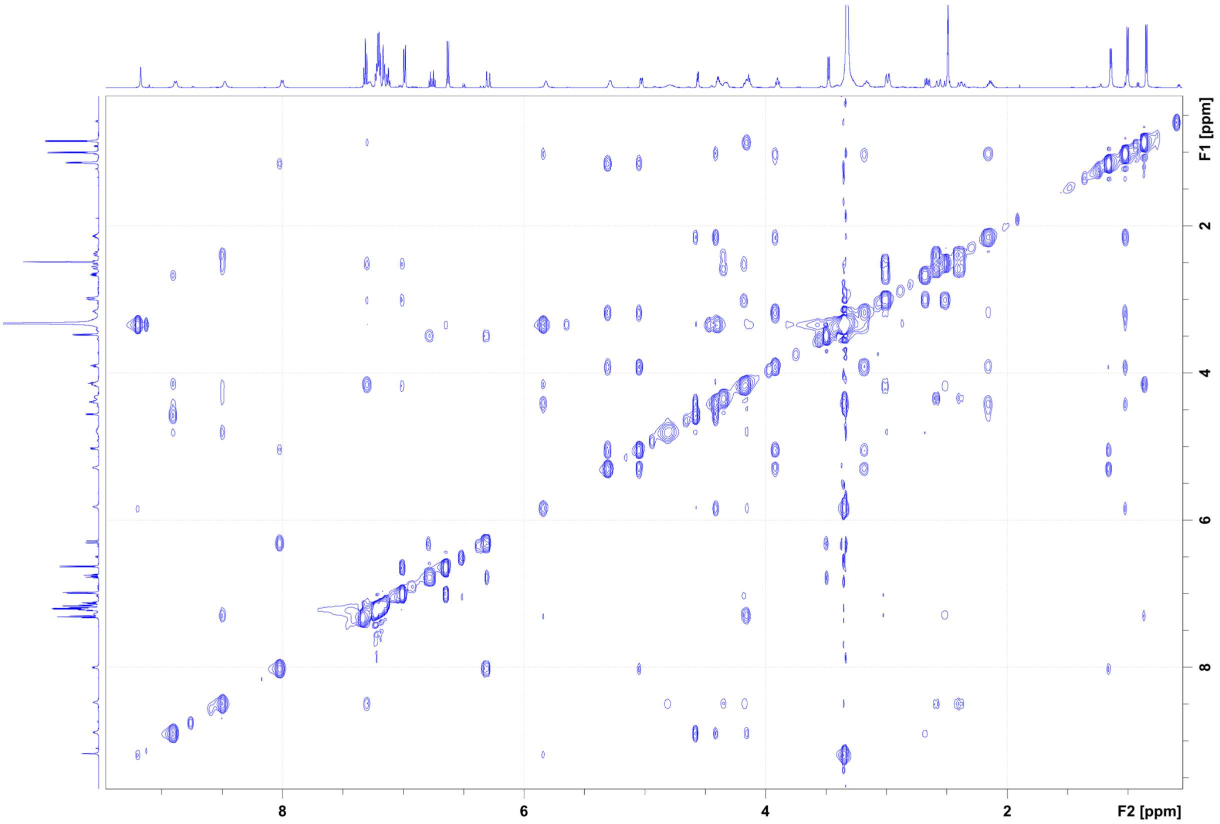


**Figure S24.** NOESY spectrum of minutumamide B1 (**3**) in DMSO-$d_6$ (600 MHz).

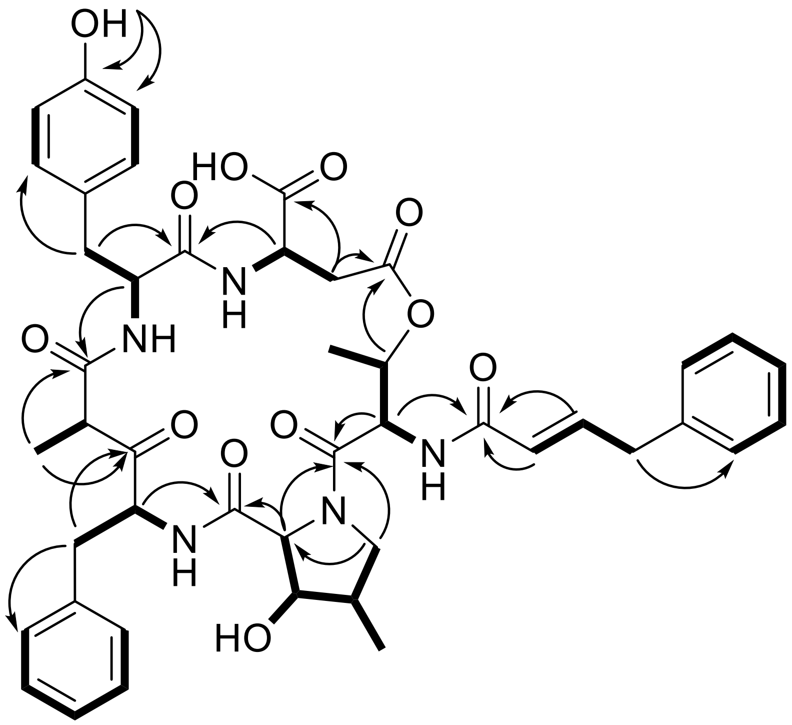


**Figure S25.** Observed and selected $^{1}H$-$^{1}H$ COSY (bold line) and HMBC (arrow) correlations of minutumamide B1 (**3**).

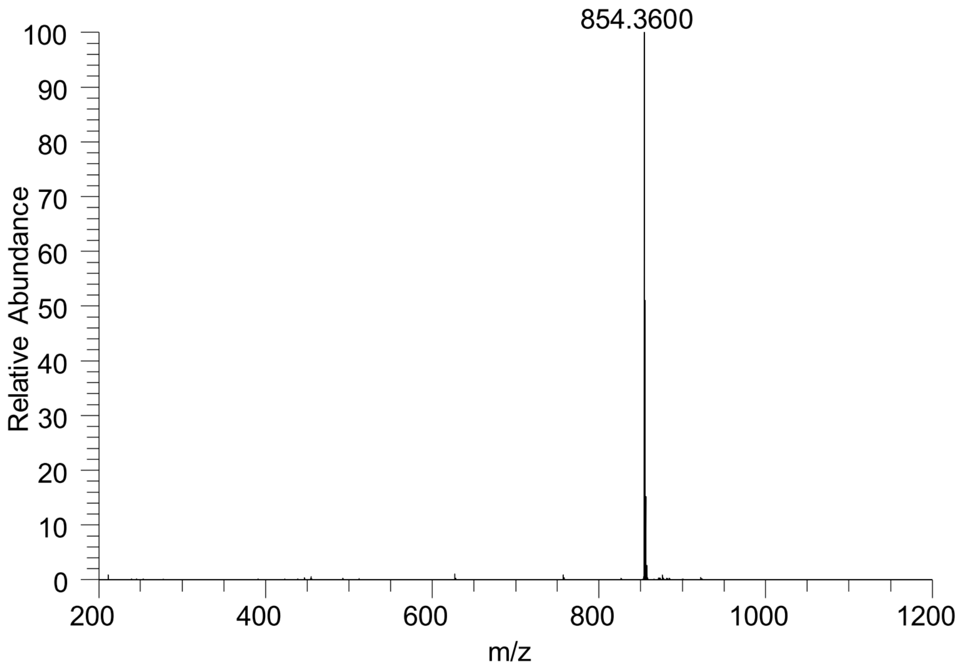

**Figure S26.** High-resolution mass spectrum in positive ion mode of minutumamide B2 (**4**).

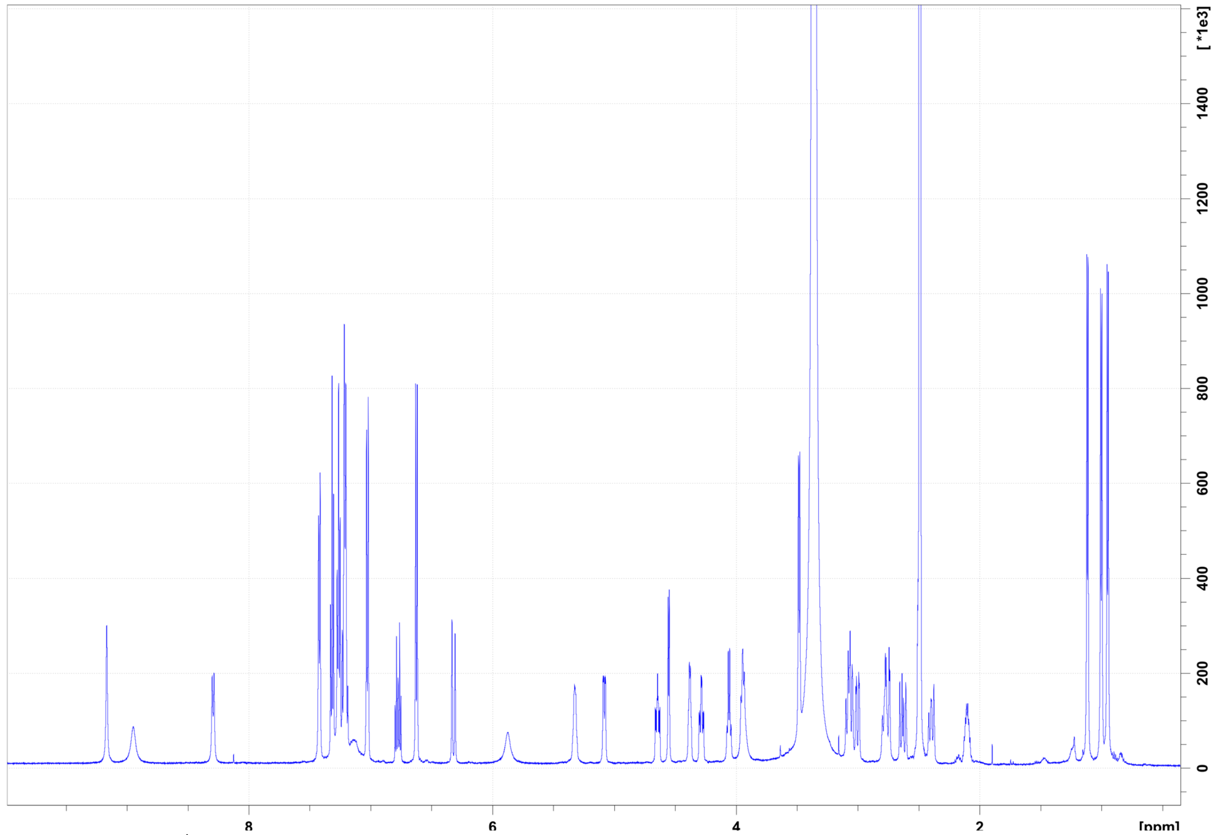


**Figure S27.** $^{1}H$ NMR spectrum of minutumamide B2 (**4**) in DMSO-$d_6$ (600 MHz).

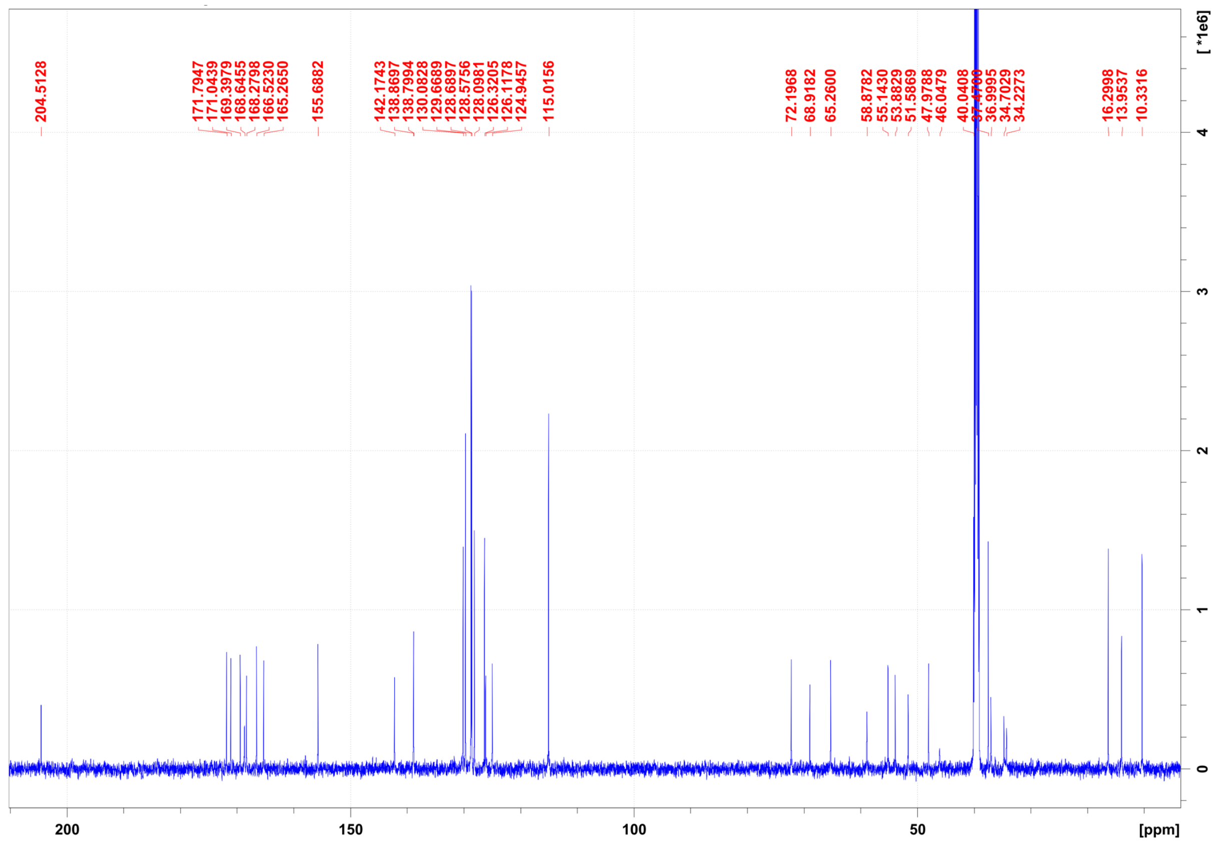


**Figure S28.** $^{13}C$ NMR spectrum of minutumamide B2 (**4**) in DMSO-$d_6$ (150 MHz).

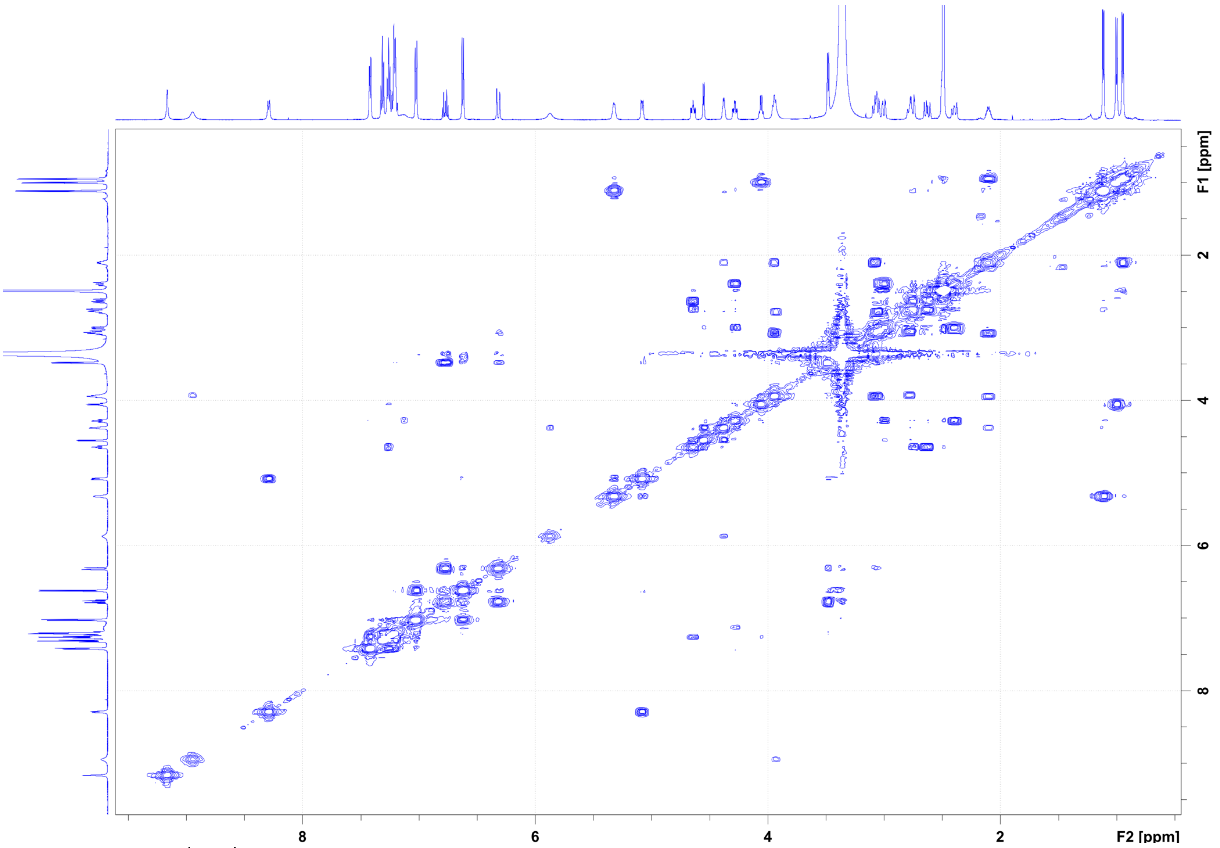


**Figure S29.** $^{1}H$-$^{1}H$ COSY spectrum of minutumamide B2 (**4**) in DMSO-$d_6$ (600 MHz).

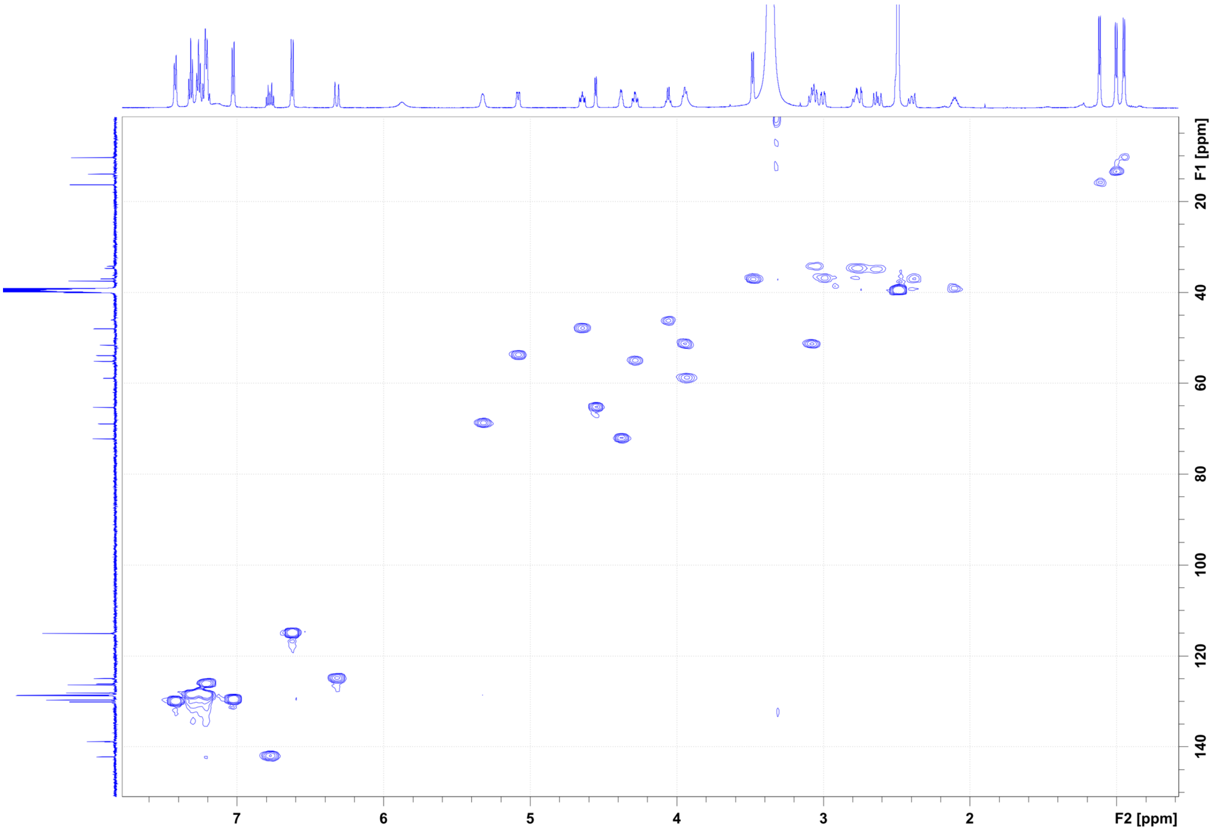


**Figure S30.** HSQC spectrum of minutumamide B2 (**4**) in DMSO-$d_6$ (600 MHz).

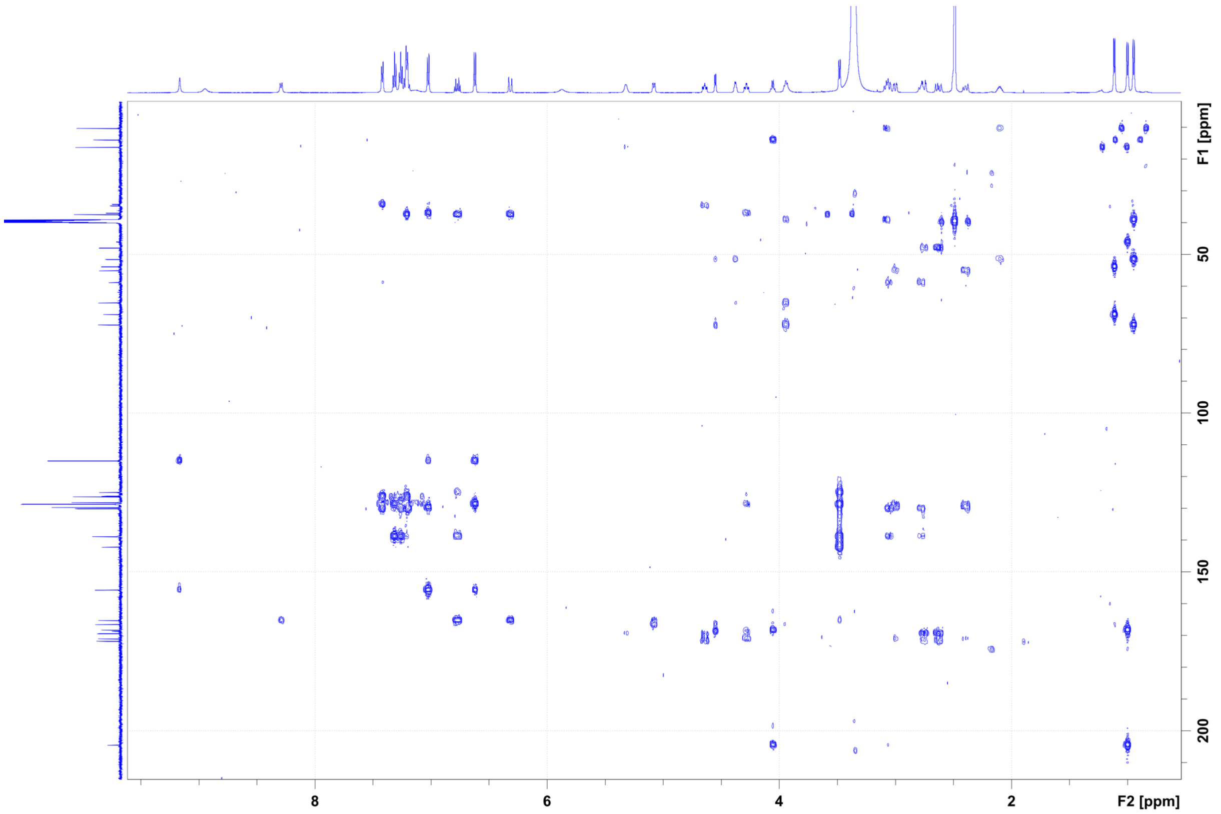


**Figure S31.** HMBC spectrum of minutumamide B2 (**4**) in DMSO-$d_6$ (600 MHz).

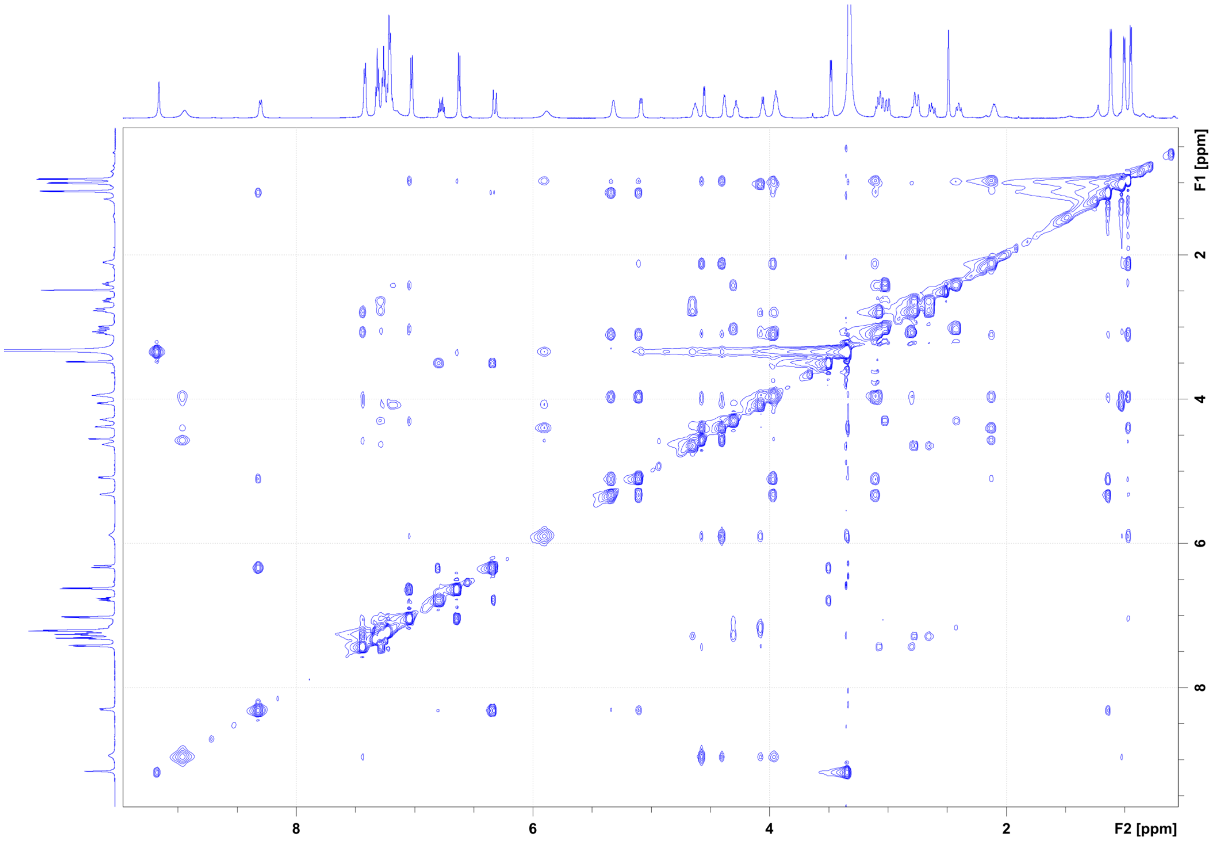


**Figure S32.** NOESY spectrum of minutumamide B2 (**4**) in DMSO-$d_6$ (600 MHz).

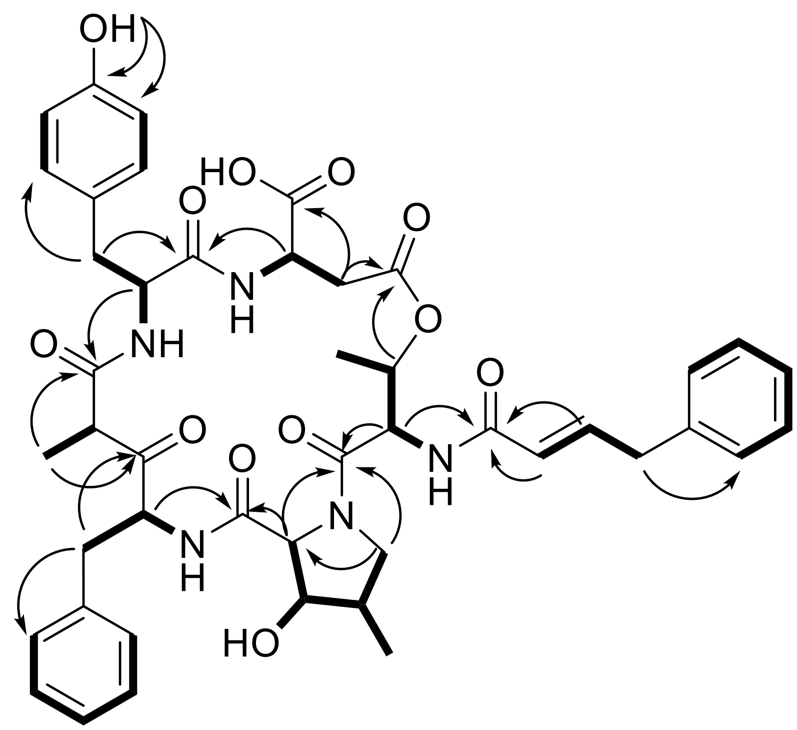


**Figure S33.** Observed and selected $^1H$-$^1H$ COSY (bold line) and HMBC (arrow) correlations of minutumamide B2 (**4**).

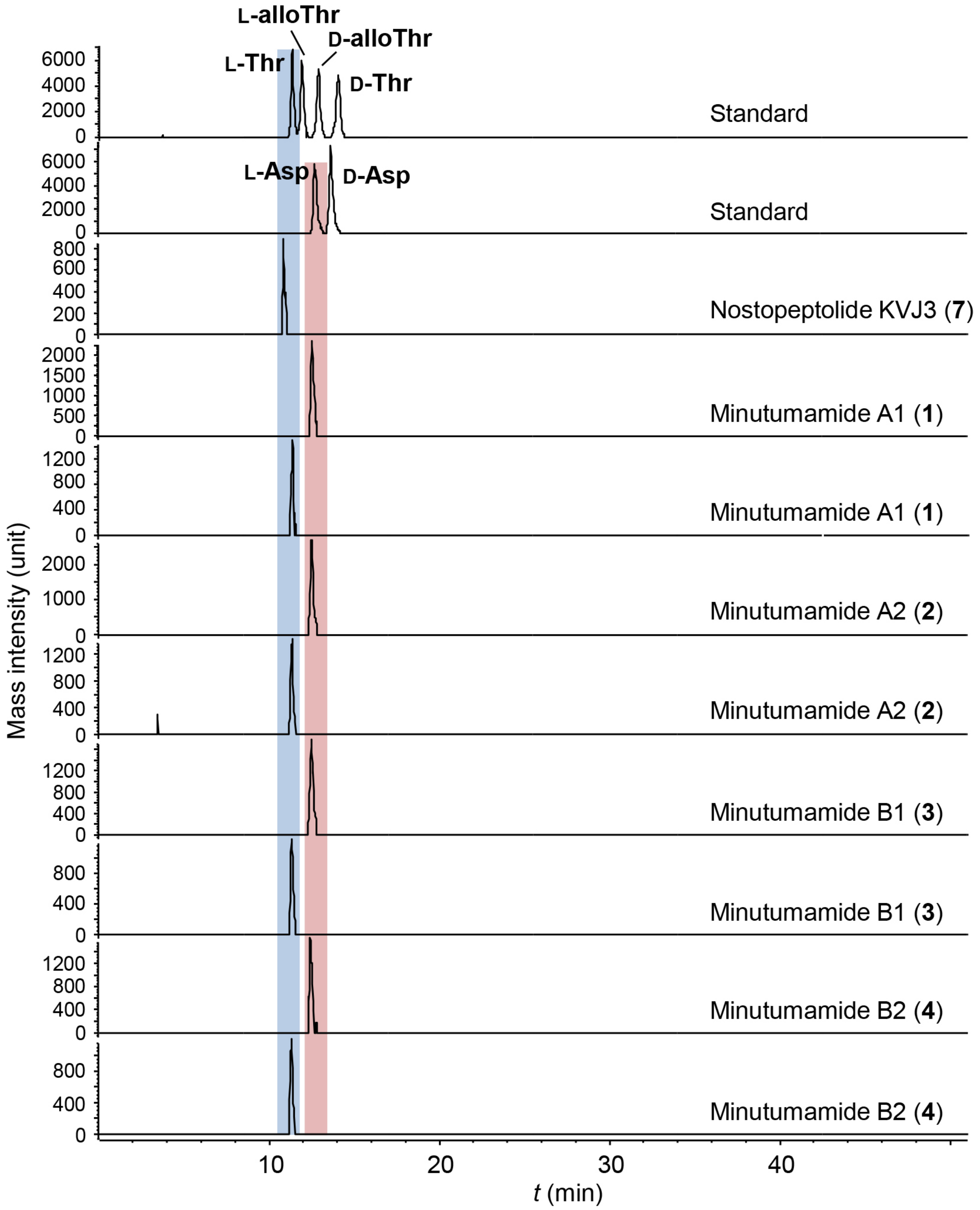


**Figure S34.** HPLC-MS profile showing the extracted ion chromatogram (EIC) of L-DAA-Thr and -Asp derivatives from acid hydrolysates of nostopeptolide KVJ3 (**7**) and minutumamides (**1**–**4**). EIC of L-DAA-Thr; *m/z* 372.373 $[M + H]^+$, EIC of L-DAA-Asp; *m/z* 386.387 $[M + H]^+$.

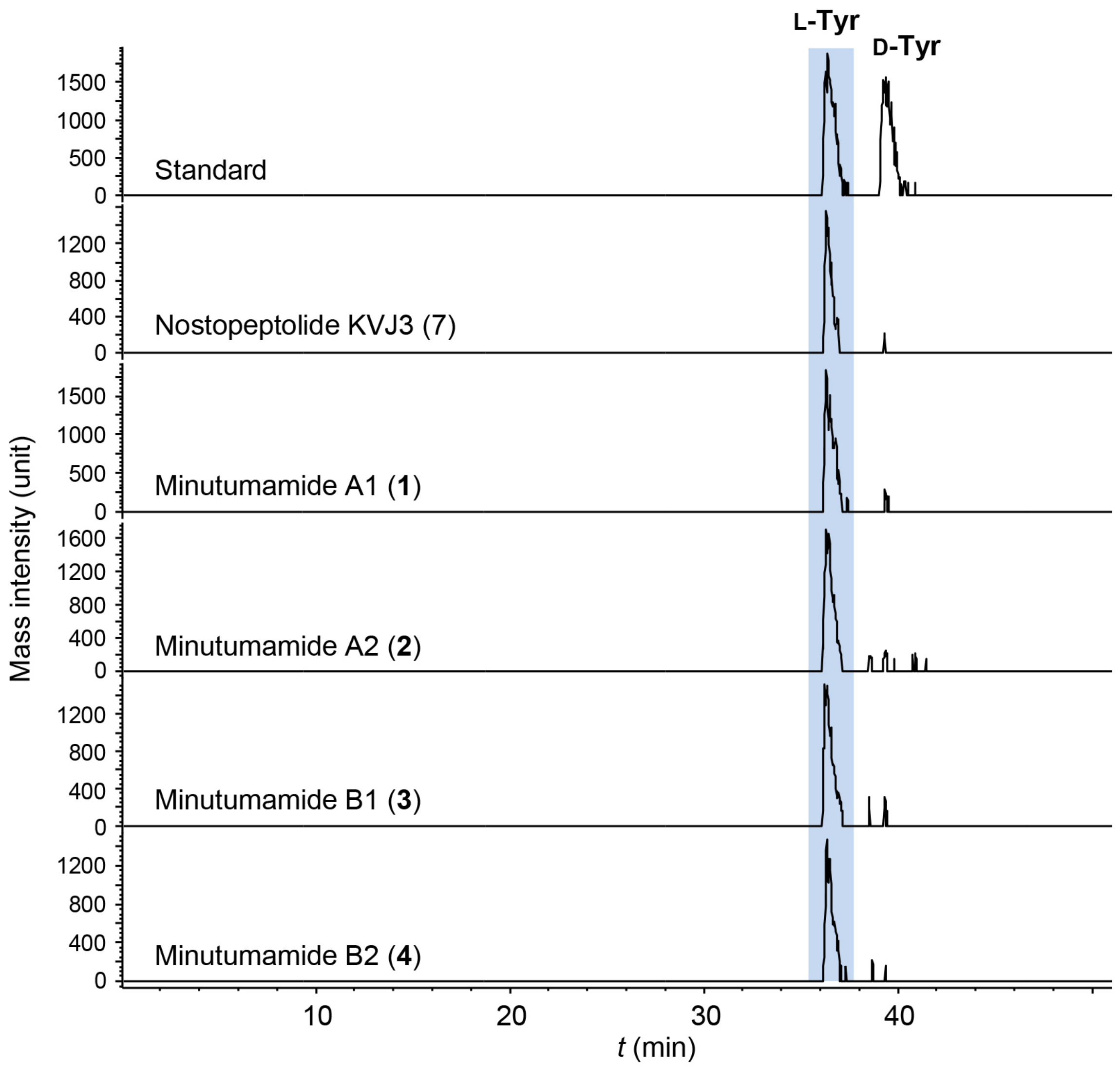


**Figure S35.** HPLC-MS profile showing the extracted ion chromatogram (EIC) of *N,O*-di-L-DAA-Tyr derivatives from acid hydrolysates of nostopeptolide KVJ3 (**7**) and minutumamides (**1**–**4**). EIC of *N,O*-di-L-DAA-Tyr; *m/z* 686.687 [M + H]$^+$.

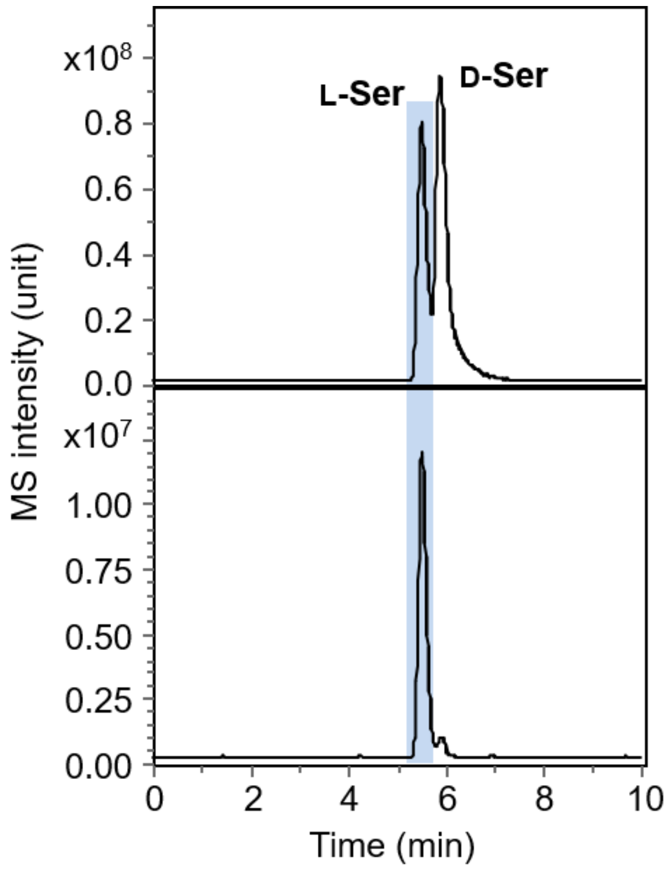


**Figure S36.** HPLC-MS profile showing the extracted ion chromatogram (EIC) of L-DAA-Ser derivatives from acid hydrolysates of nostopeptolide KVJ3 (bottom panel). EIC of L-DAA-Ser; *m/z* 358.10 $[M + H]^+$.

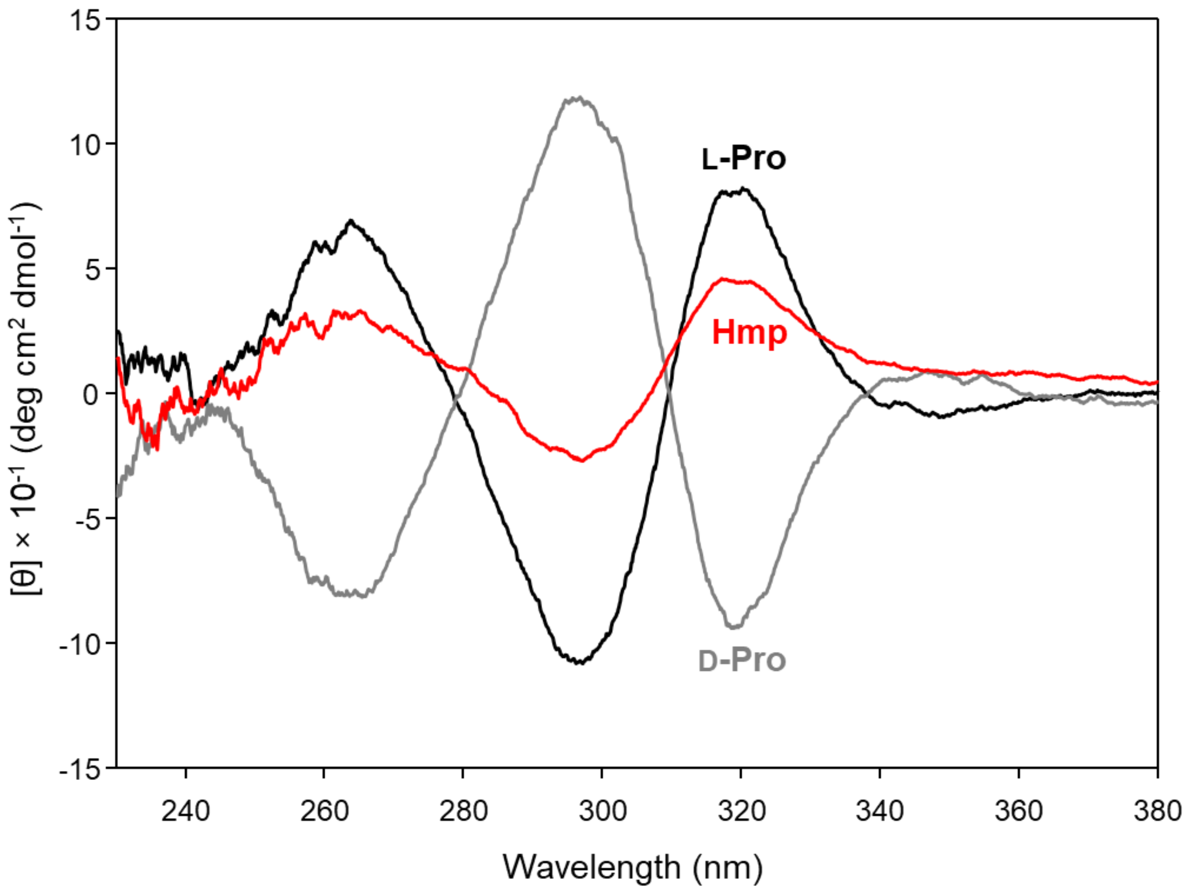


**Figure S37.** CD spectra of *in situ* L-Pro, D-Pro, and Hmp fluorescamine derivatives.

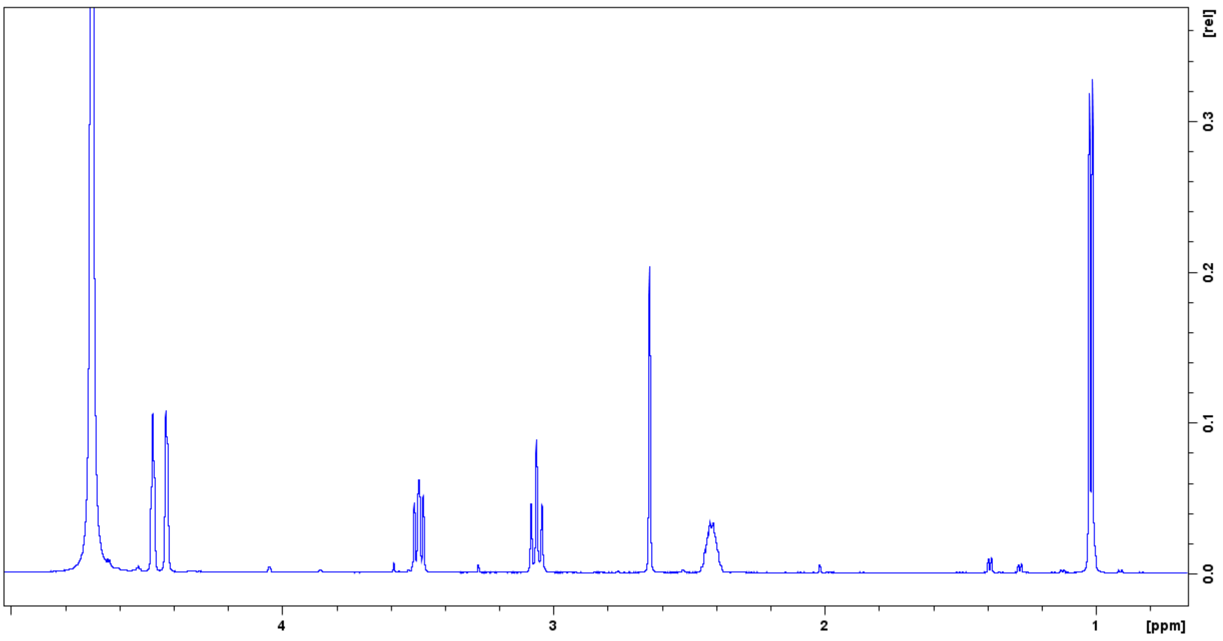


**Figure S38.** $^1H$ NMR spectrum of Hmp from minutumamide A2 (**2**) in $D_2O$.

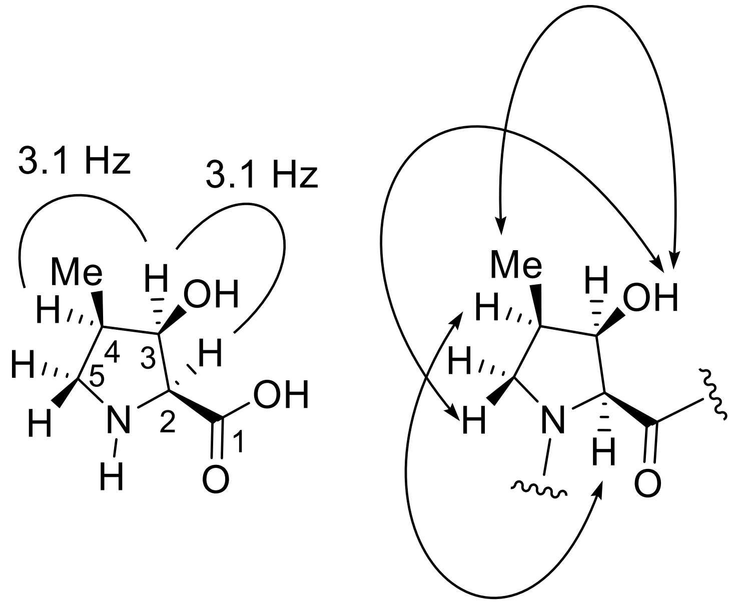


**Figure S39.** Observed small coupling constants $^{3}J_{H,H}$ of isolated (2*S*, 3*R*, 4*R*) Hmp and NOE correlations (arrows) of (2*S*, 3*R*, 4*R*) Hmp in minutumamide A2 (**2**).

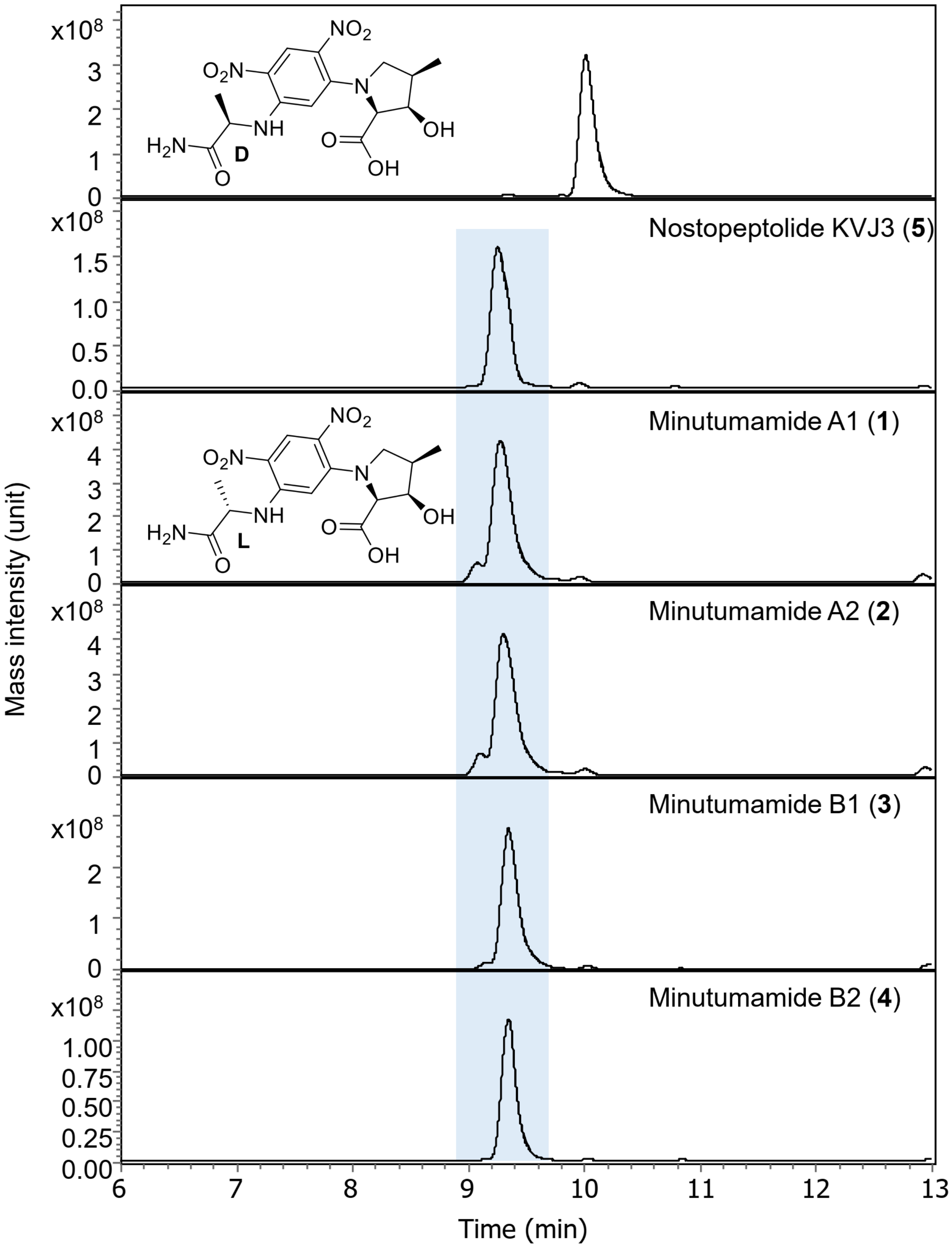


**Figure S40.** HPLC-MS profile showing the extracted ion chromatogram (EIC) of L-DAA-Hmp derivatives from acid hydrolysates of peptides. L-DAA-(2*S*, 3*R*, 4*R*) Hmp and D-DAA-(2*S*, 3*R*, 4*R*) Hmp from minutumamide A1 (**1**) were used as standards. EIC of L,D-DAA-Hmp; *m/z* 398.10 $[M + H]^+$.

**A**

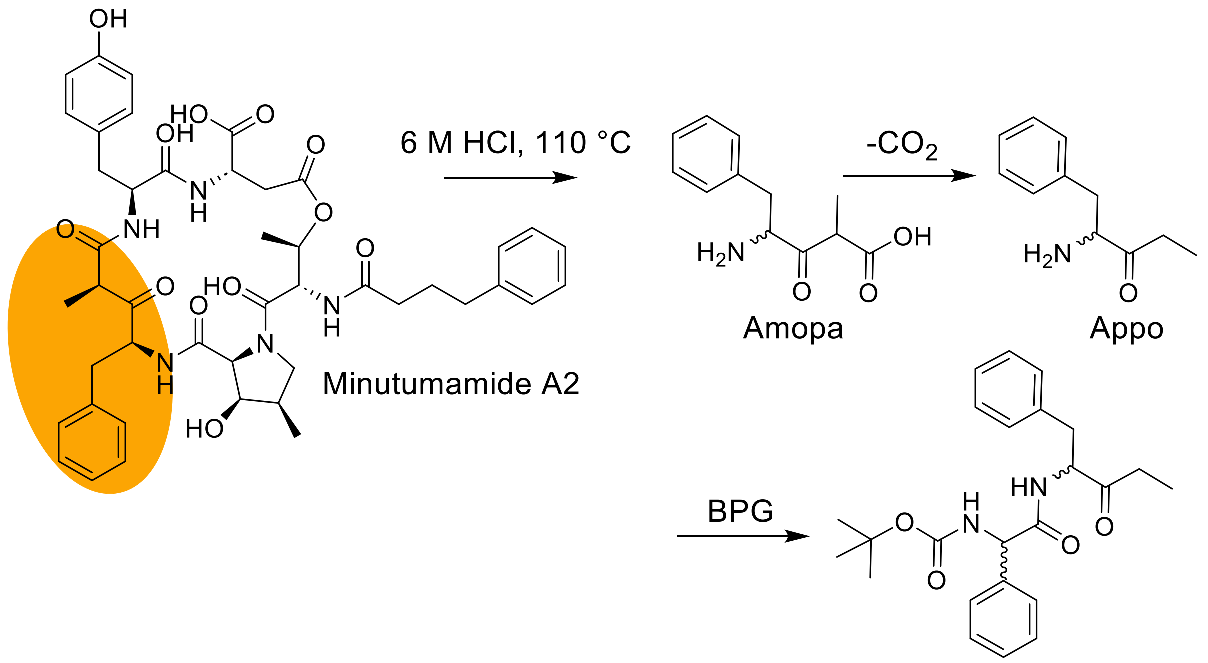


**B**

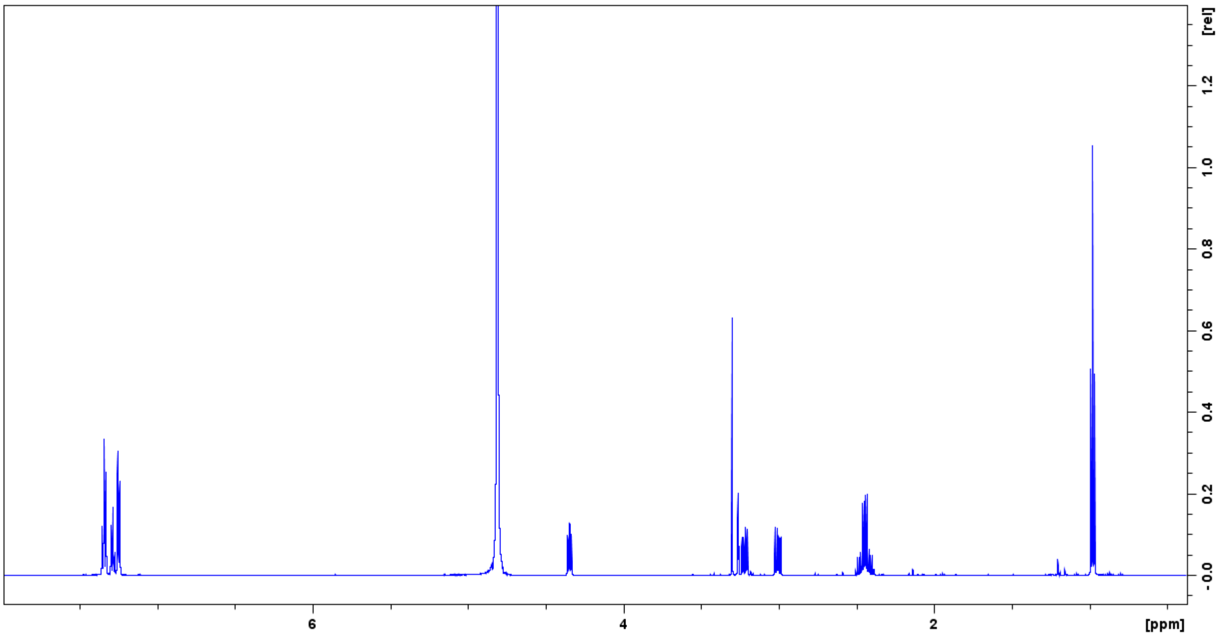


**Figure S41.** The stereochemistry elucidation of Appo obtained by the acid hydrolyzation of minutumamide A2 (**2**). **A**. Scheme of stereochemistry elucidation of Amopa. **B**. $^{1}H$ NMR spectrum of Appo from minutumamide A2 (**2**) in $CD_3OD$. BPG; Boc-phenylglycine.

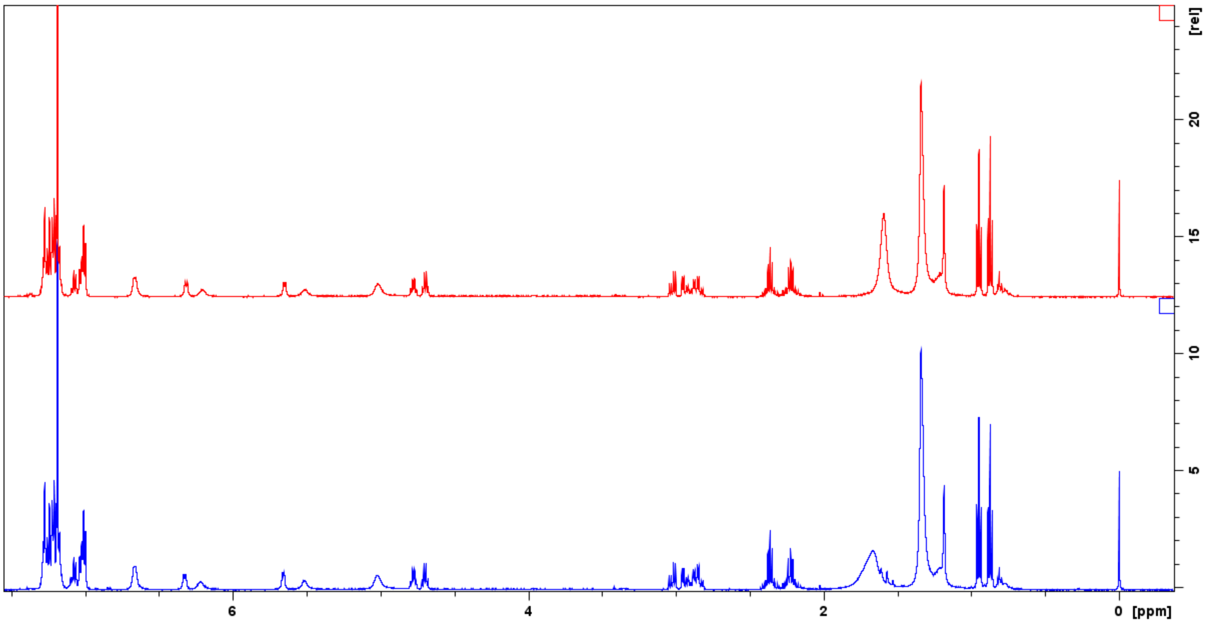


**Figure S42.** The racemic mixture of Appo clarified by chiral reagent BPG. $^{1}$H NMR spectra of D,L-BPG-Appo (D-BPG-Appo; upper spectrum, L-BPG-Appo; lower spectrum) in $CDCl_3$.

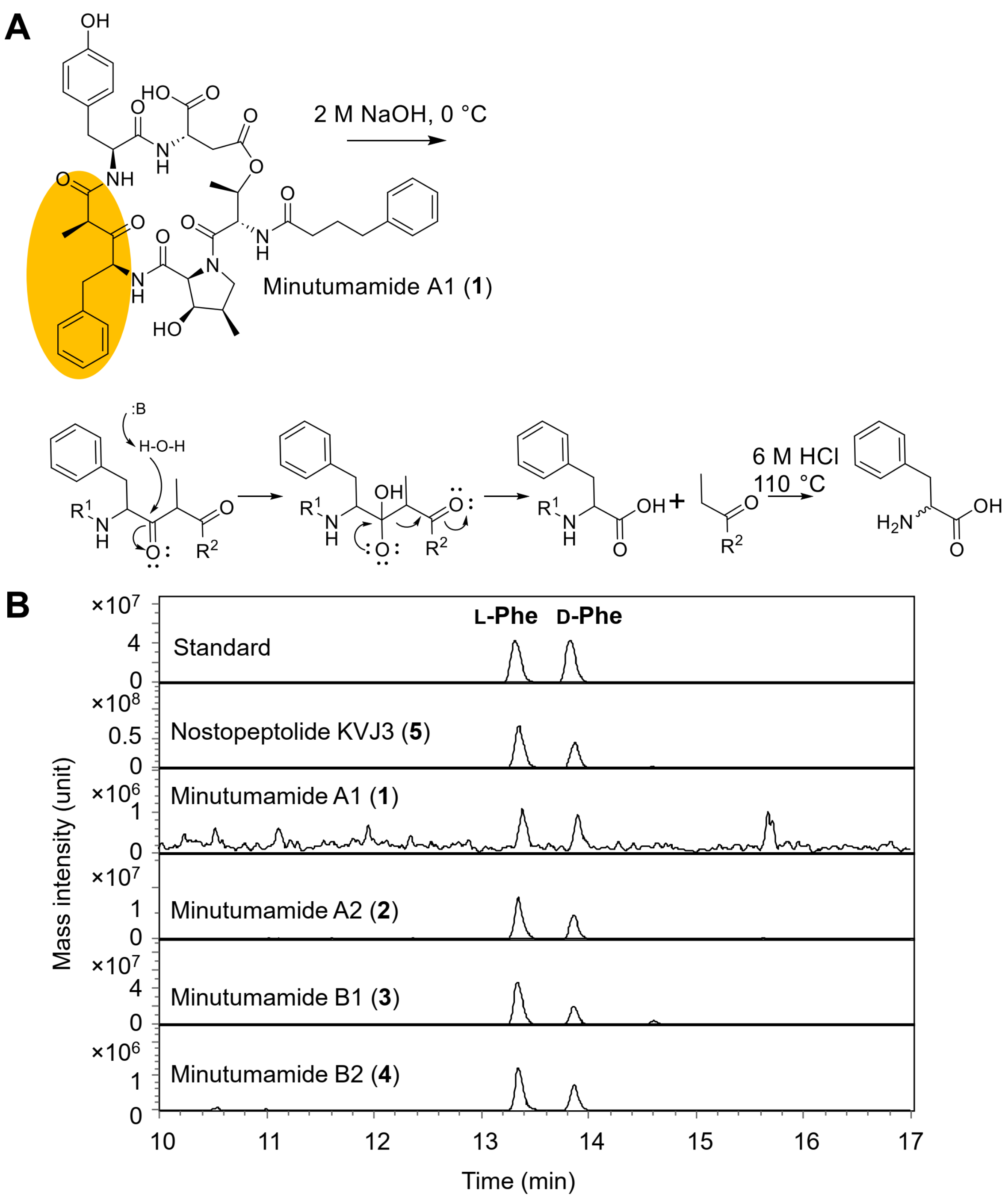


**Figure S43.** The racemic mixture of phenylalanine obtained by retro Claisen reaction of Amopa in nostopeptolide KVJ3 (**7**) and minutumamides. **A.** Scheme of chemical degradation of minutumamide A–D (**1**–**4**) and proposed retro Claisen reaction in Amopa. **B.** LC-MS profile (EIC *m/z* 418.10 [M + H]$^+$) of L-DAA-Phe obtained from retro Claisen reaction followed by acid hydrolyzation.

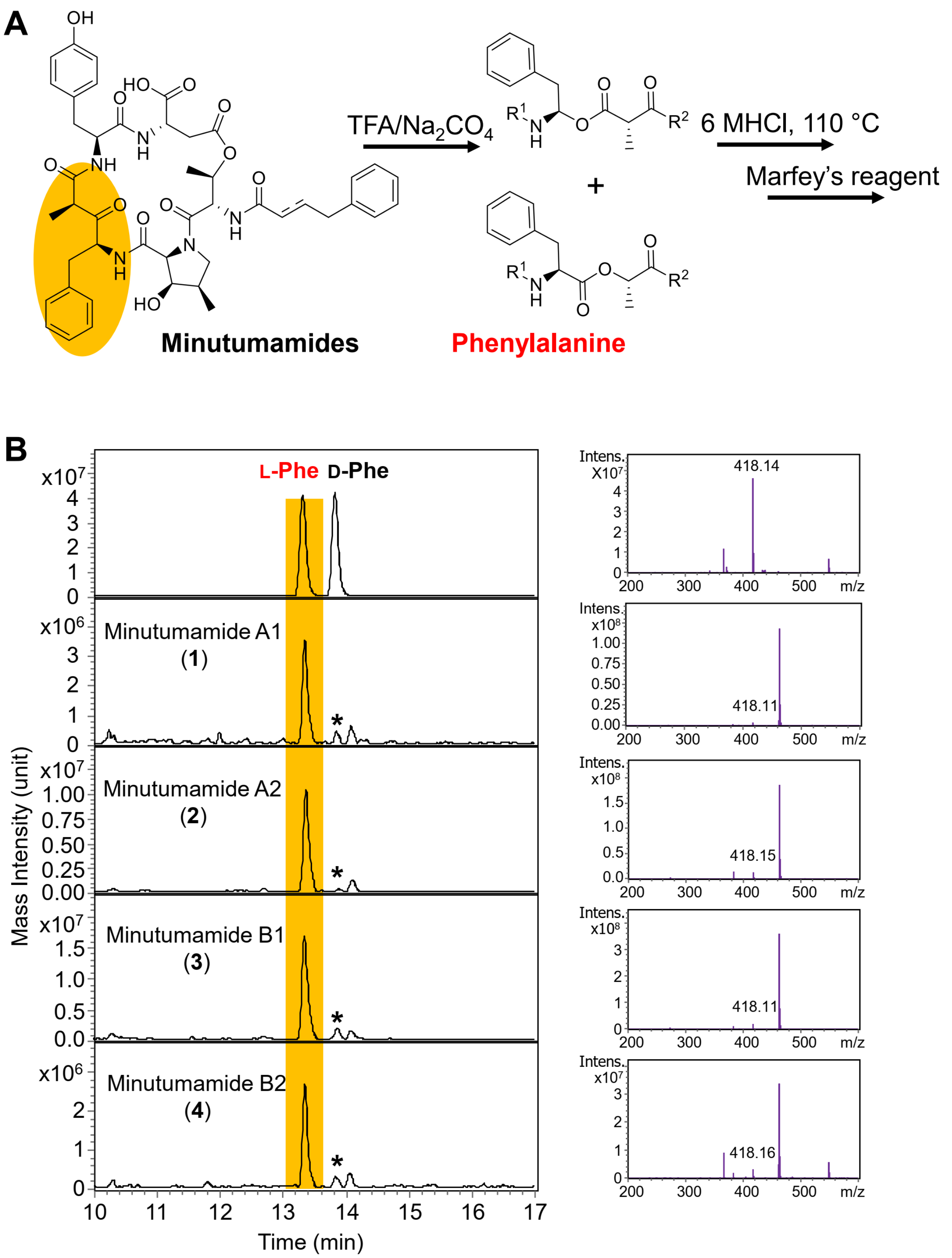


**Figure S44.** The stereochemistry determination of phenylalanine obtained by Baeyer Villiger oxidation (BVO) of Amopa in minutumamides (**1**–**4**). **A.** Scheme of chemical degradation and modification of minutumamides (**1**–**4**). **B.** LC-MS profile (EIC *m/z* 418.10 $[M + H]^+$) and their ions of L-DAA-phenylalanine obtained from BVO followed by acid hydrolyzation. *; Small amount of racemic D-Phe (L-DAA-D-Phe) was observed.

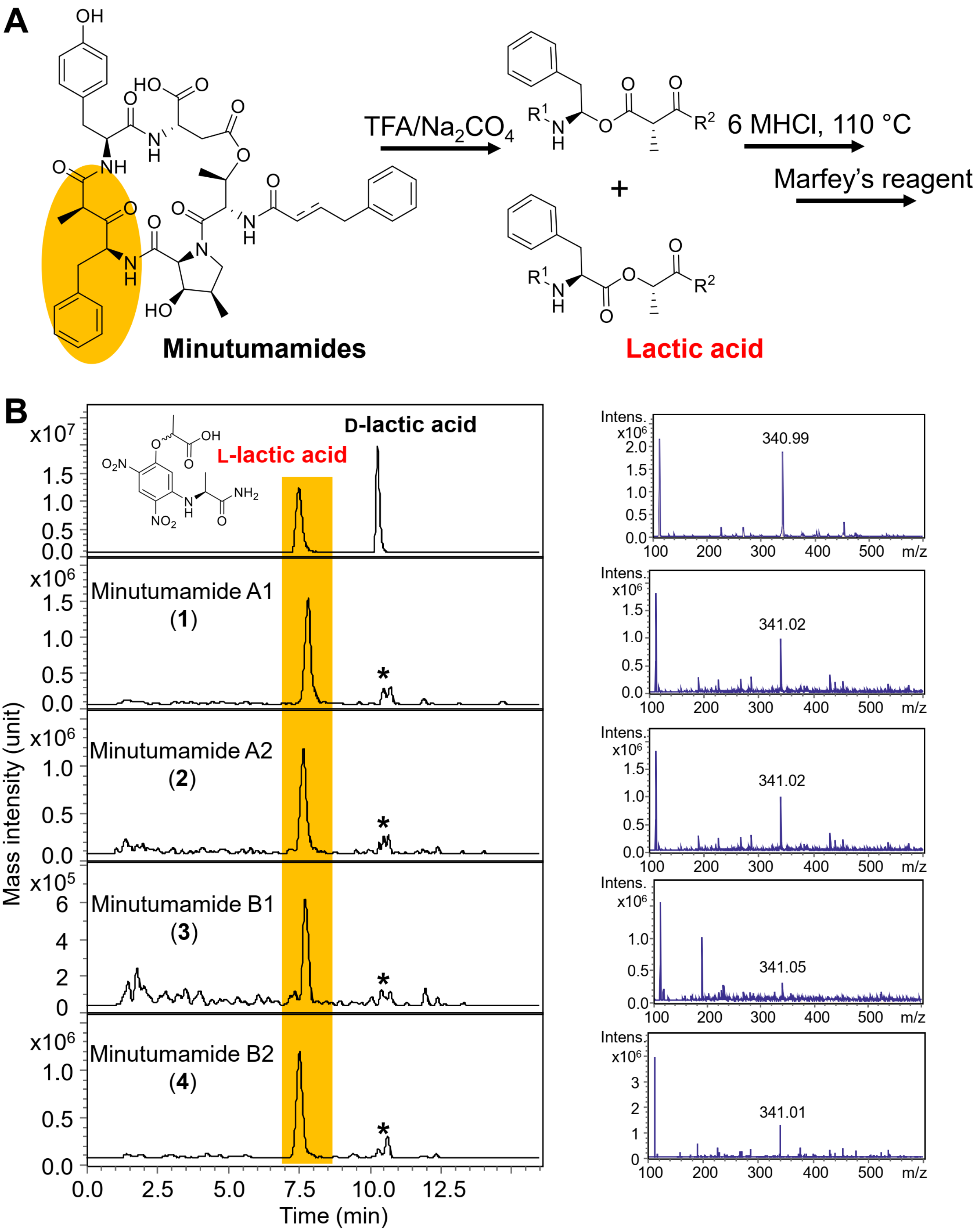


**Figure S45.** The stereochemistry determination of lactic acid obtained by Baeyer Villiger oxidation (BVO) of Amopa in minutumamides (**1**–**4**). **A.** Scheme of chemical degradation and modification of minutamamides (**1**–**4**). **B.** LC-MS profile (EIC *m/z* 341.00 [M - H]$^-$) and their ions of L-DAA-lactic acid obtained from BVO followed by acid hydrolyzation. *; Small amount of racemic D-lactic acid (L-DAA-D-lactic acid) was observed.

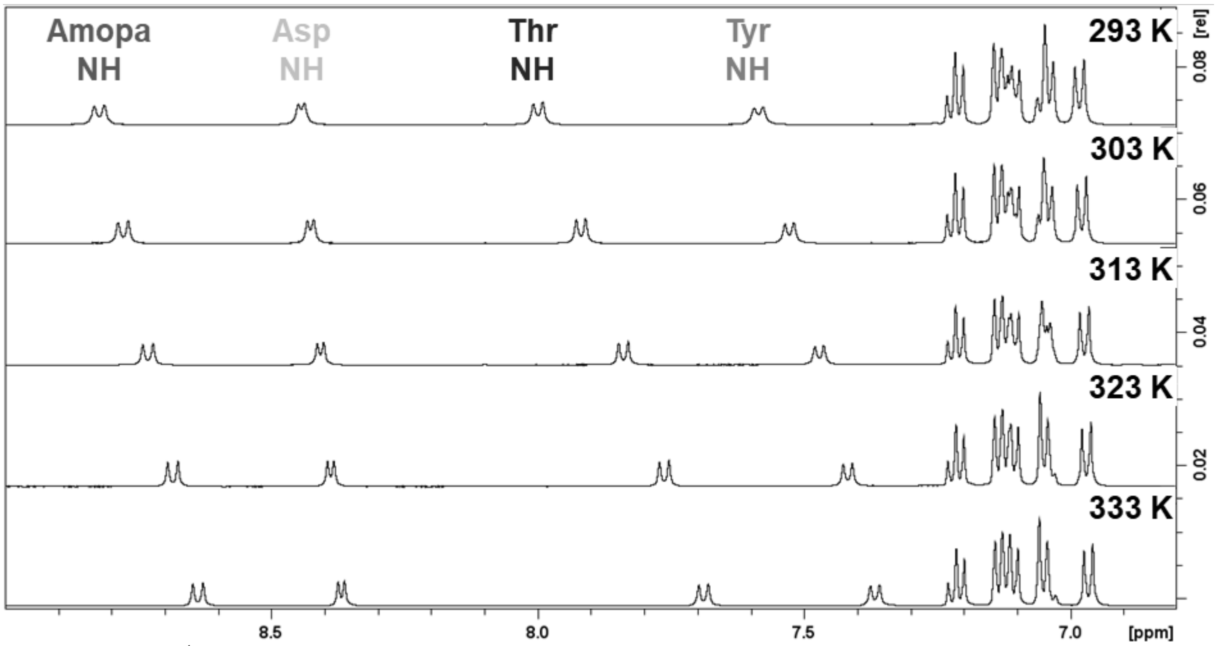


**Figure S46.** $^{1}$H NMR spectra in amide proton region of minutumamide A1 (**1**) at 293 – 333 K.

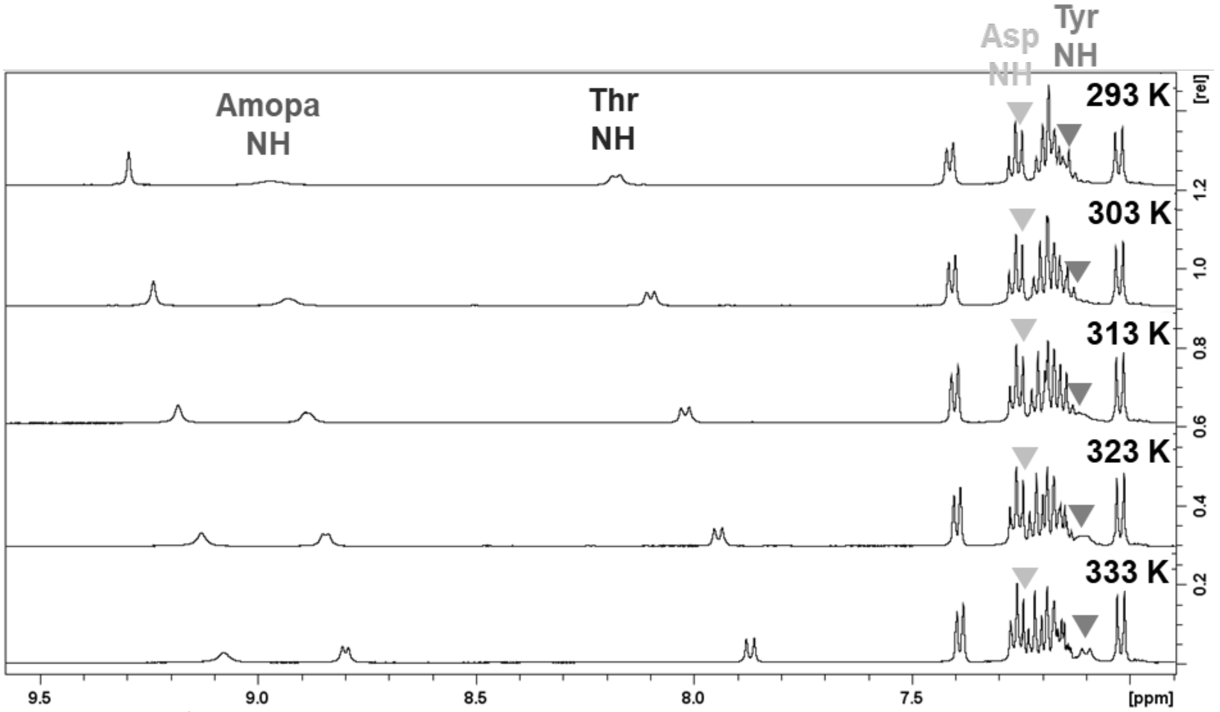


**Figure S47.** $^{1}$H NMR spectra in amide proton region of minutumamide A2 (**2**) at 293 – 333 K.

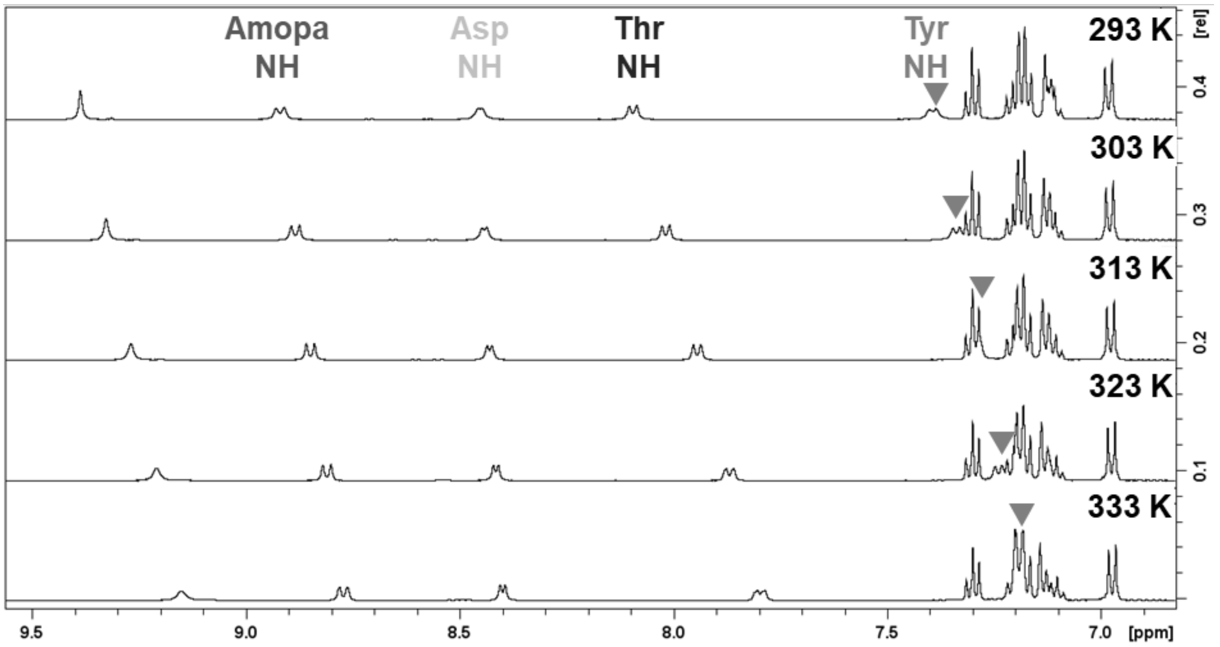


**Figure S48.** $^1$H NMR spectra in amide proton region of minutumamide B1 (**3**) at 293 – 333 K.

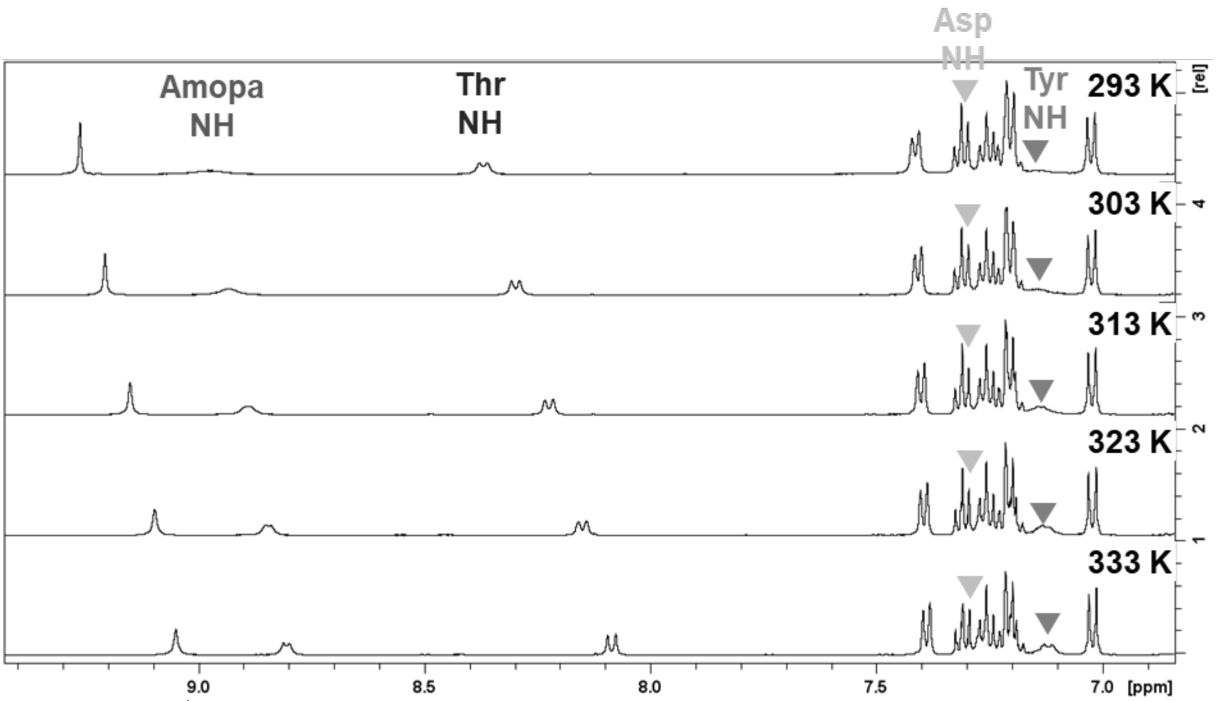


**Figure S49.** $^1$H NMR spectra in amide proton region of minutumamide B2 (**4**) at 293 – 333 K.

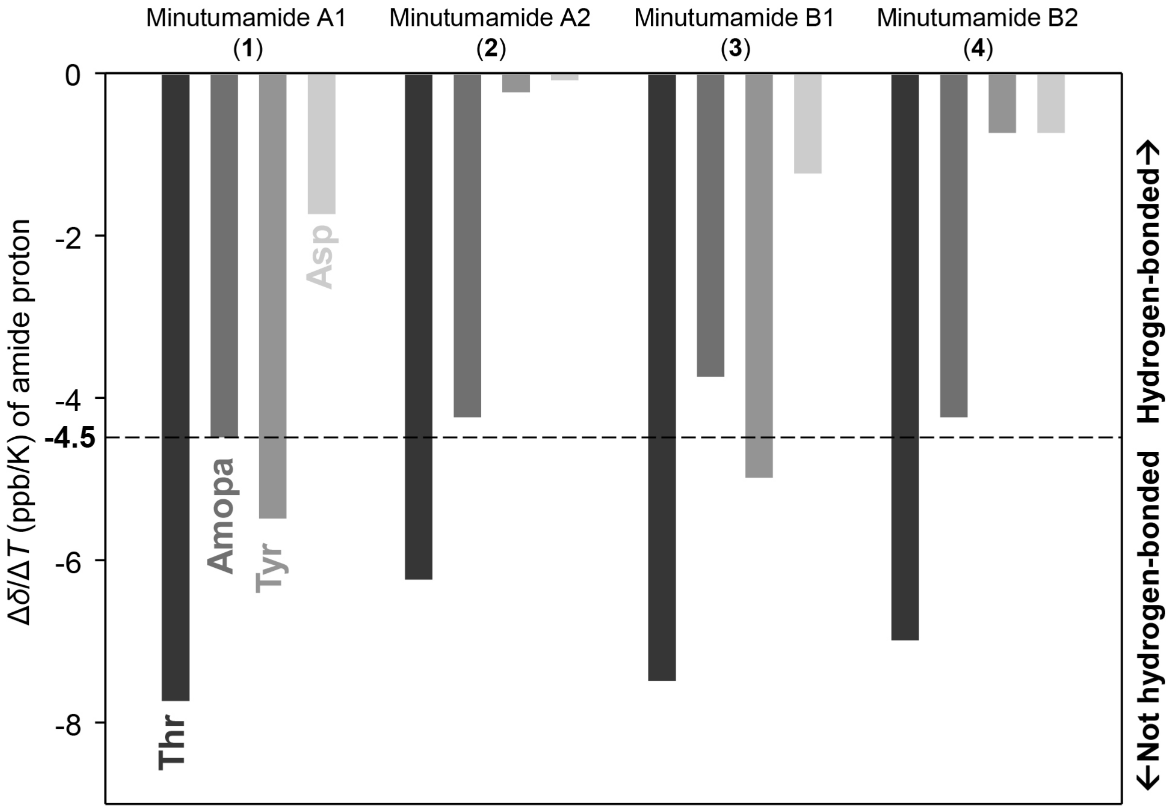


**Figure S50.** Difference of hydrogen bonded amide protons between minutumamide A1 (**1**) and A2 (**2**) (B1 (**3**) and B2 (**4**)). The value of –4.5 ppb/K (dotted line) is the cutoff value for hydrogen-bonded amides proposed by Baxter and Williamson[16]. Gray scale bars correspond with each amino acids.

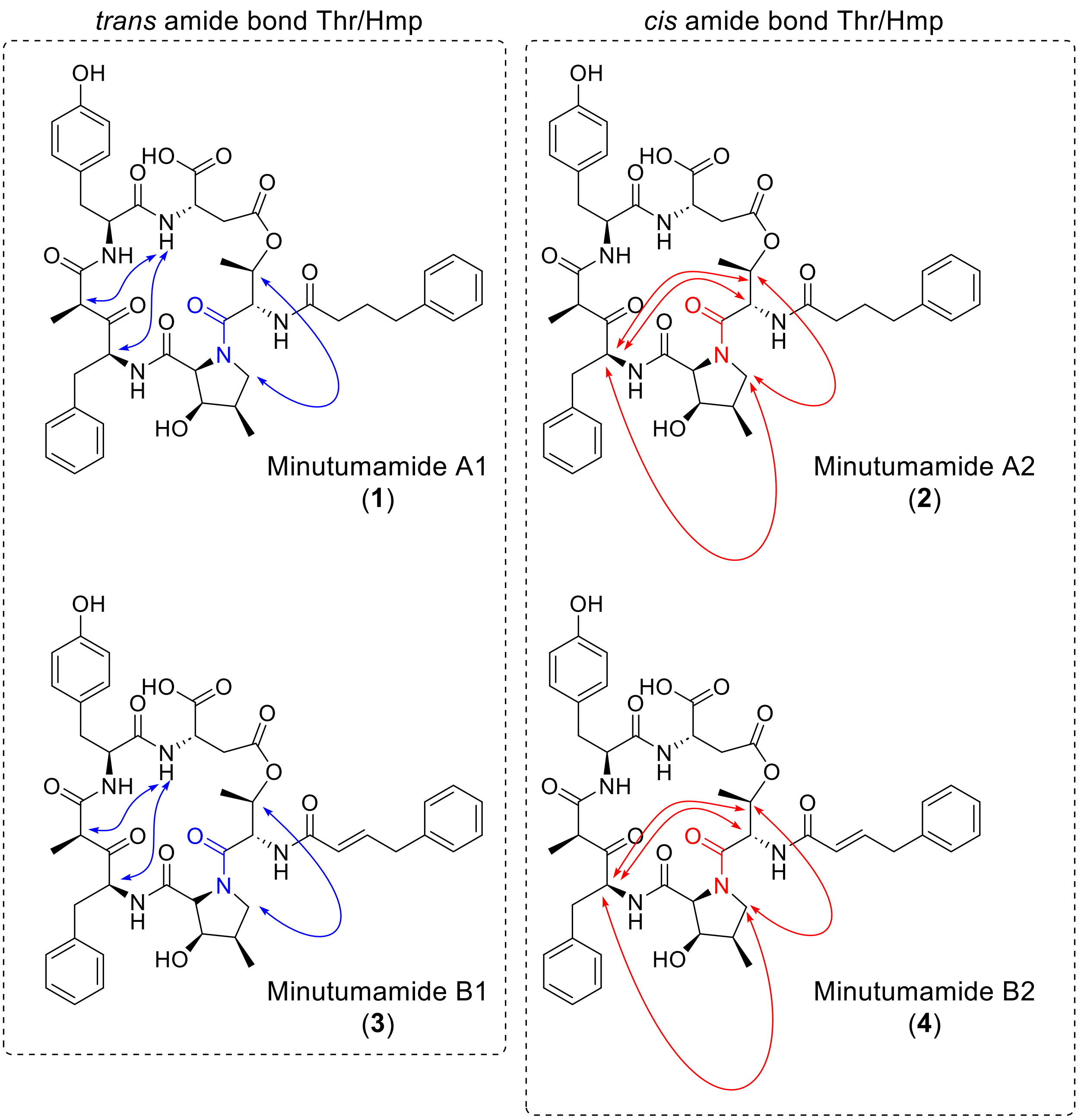


**Figure S51.** Observed and selected long-range NOEs (arrows) among minutumamides. Minutumamide A1 (**1**) and B1 (**3**) possess a *trans* amide bond between Thr and Hmp (blue arrows and amide bond). Minutumamide A2 (**2**) and B2 (**4**) possess a *cis* amide bond between Thr and Hmp (red arrows and amide bond).

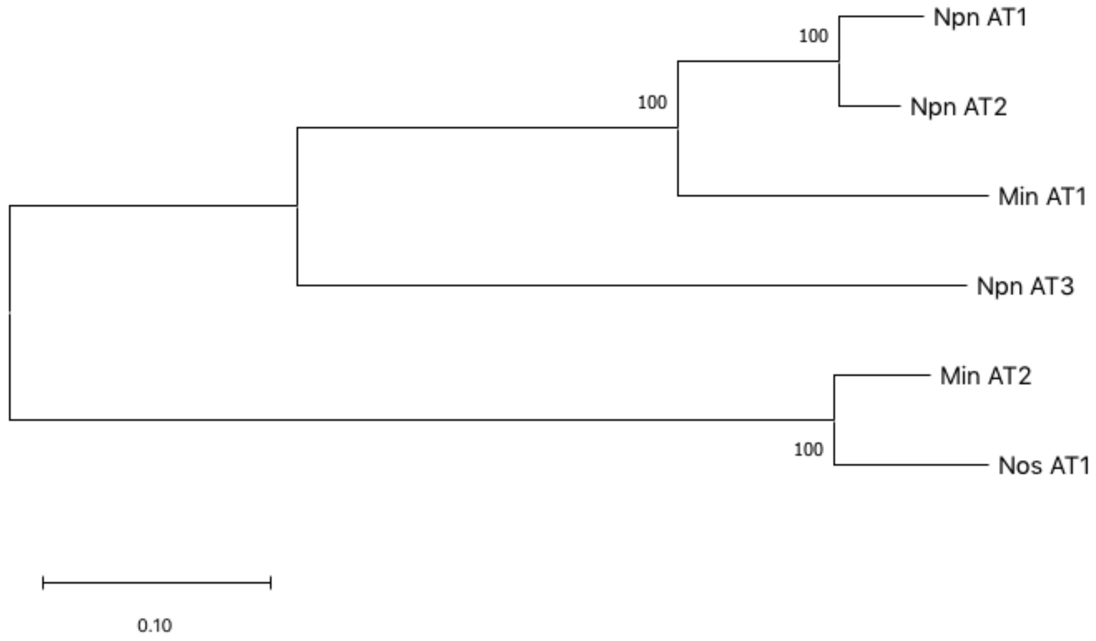


**Figure S52.** Phylogenetic analysis of acyltransferase (AT) domains from minutumamide (Min), nostopeptolide (Nos), and nostophycin (Npn) biosynthesis. The close relationship between domains encoded in the *min* cluster and domains encoded by *npn*, as well as *nos* biosynthesis genes indicates a shared evolutionary history.

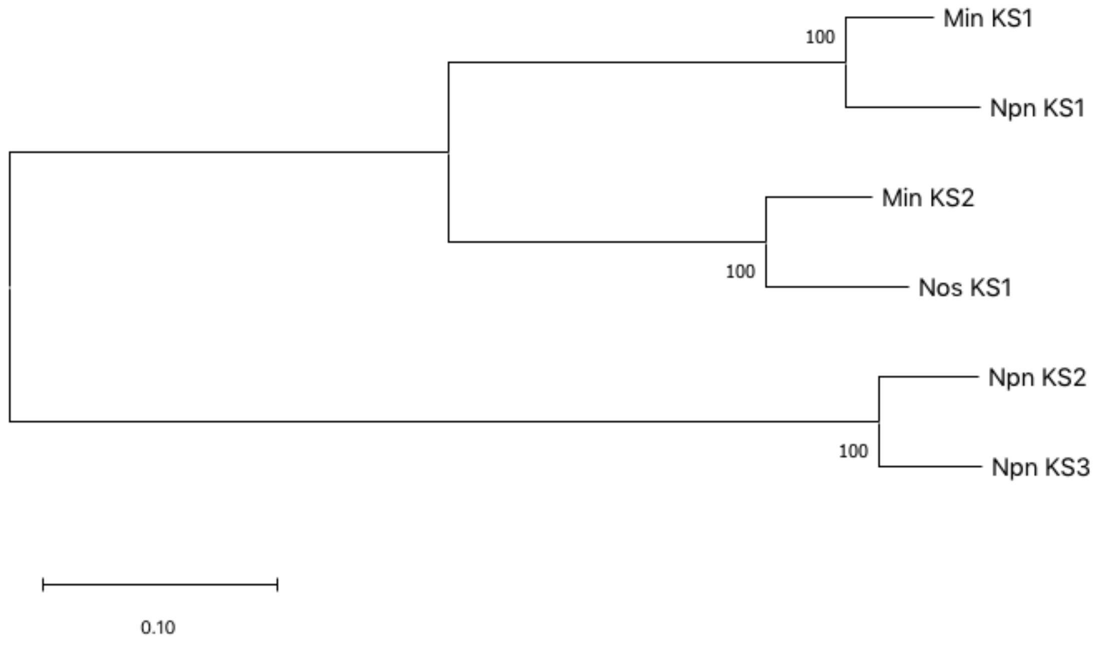

**Figure S53.** Phylogenetic analysis of ketosynthase (KS) domains from minutumamide (Min), nostopeptolide (Nos), and nostophycin (Npn) biosynthesis. The close relationship between domains encoded in the *min* cluster and domains encoded by *npn*, as well as *nos* biosynthesis genes indicates a shared evolutionary history.

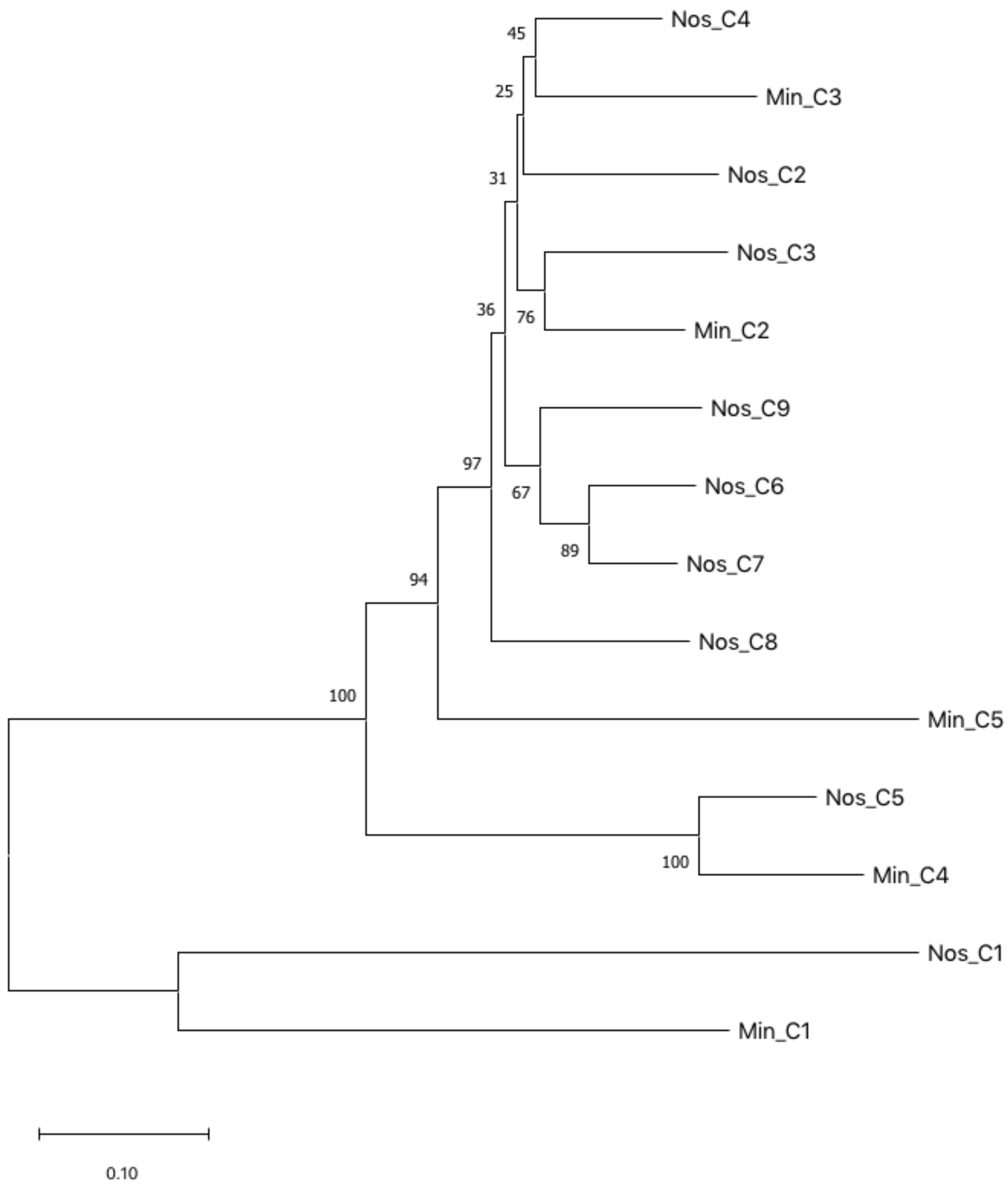


**Figure S54.** Phylogenetic analysis of condensation (C) domains from minutumamide (Min) and nostopeptolide (Nos) biosynthesis. The close relationship between domains encoded in the *min* cluster and domains encoded by *nos* biosynthesis genes indicates a shared evolutionary history.

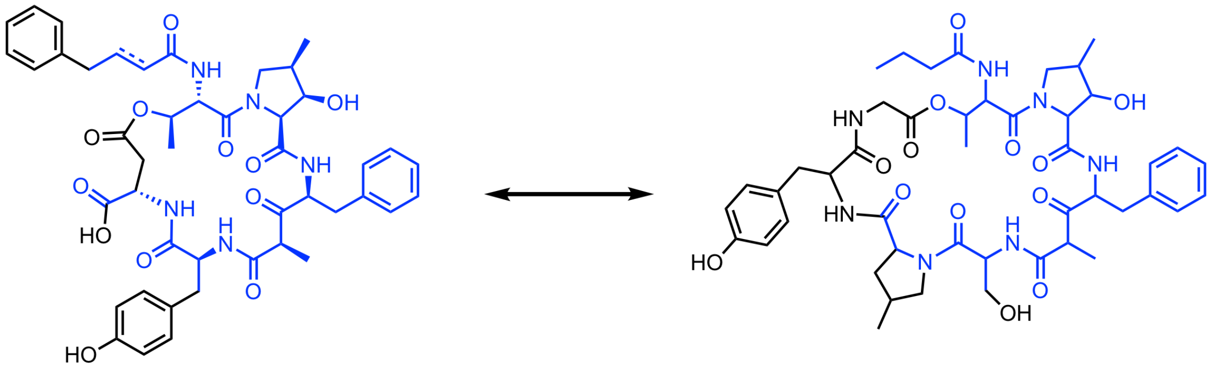


Minutumamides (**1**–**4**)    *In-silico* prediction of Nostopeptolide KVJ3

**Figure S55.** Structure-guided genome mining. Proposed structure of a ring-contracted 7-membered nostopeptolide congener from *Nostoc* sp. KVJ3 that markedly resembles the macrolactone ring of minutunamides (**1**–**4**) but lacks the characteristic aromatic side chain of minutumamide.

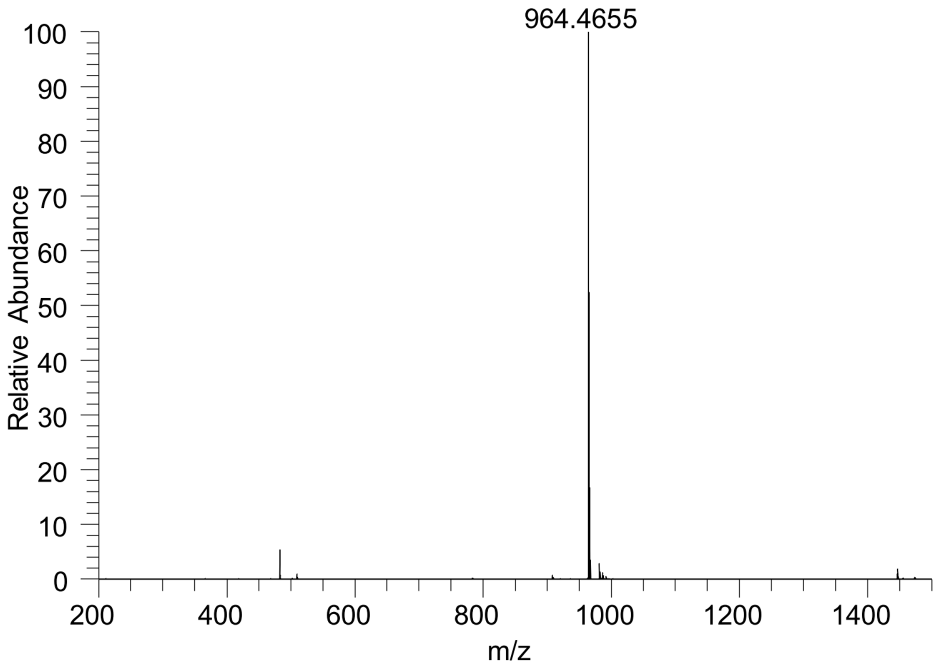


**Figure S56.** High-resolution mass spectrum in positive ion mode of nostopeptolide KVJ3 (**7**).

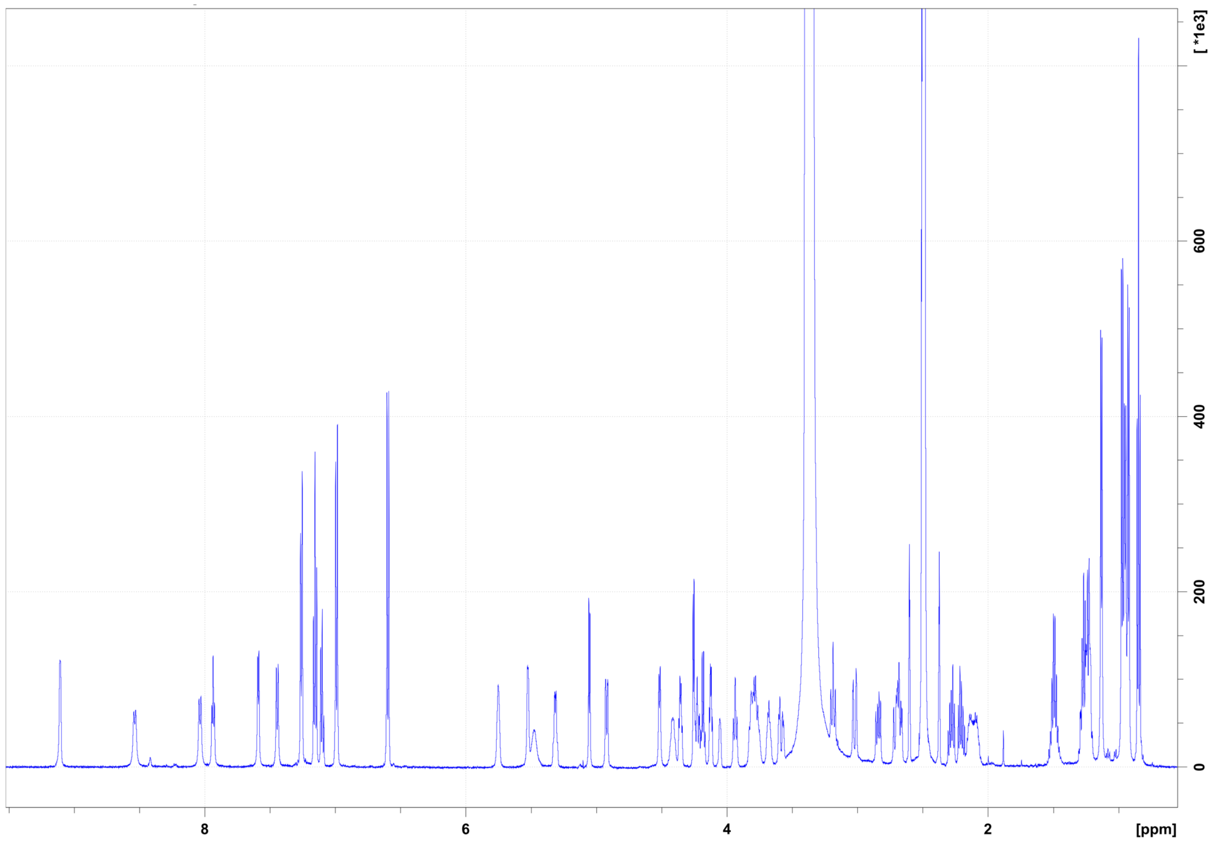

**Figure S57.** $^{1}H$ NMR spectrum of nostopeptolide KVJ3 (**7**) in DMSO-$d_6$ (600 MHz).

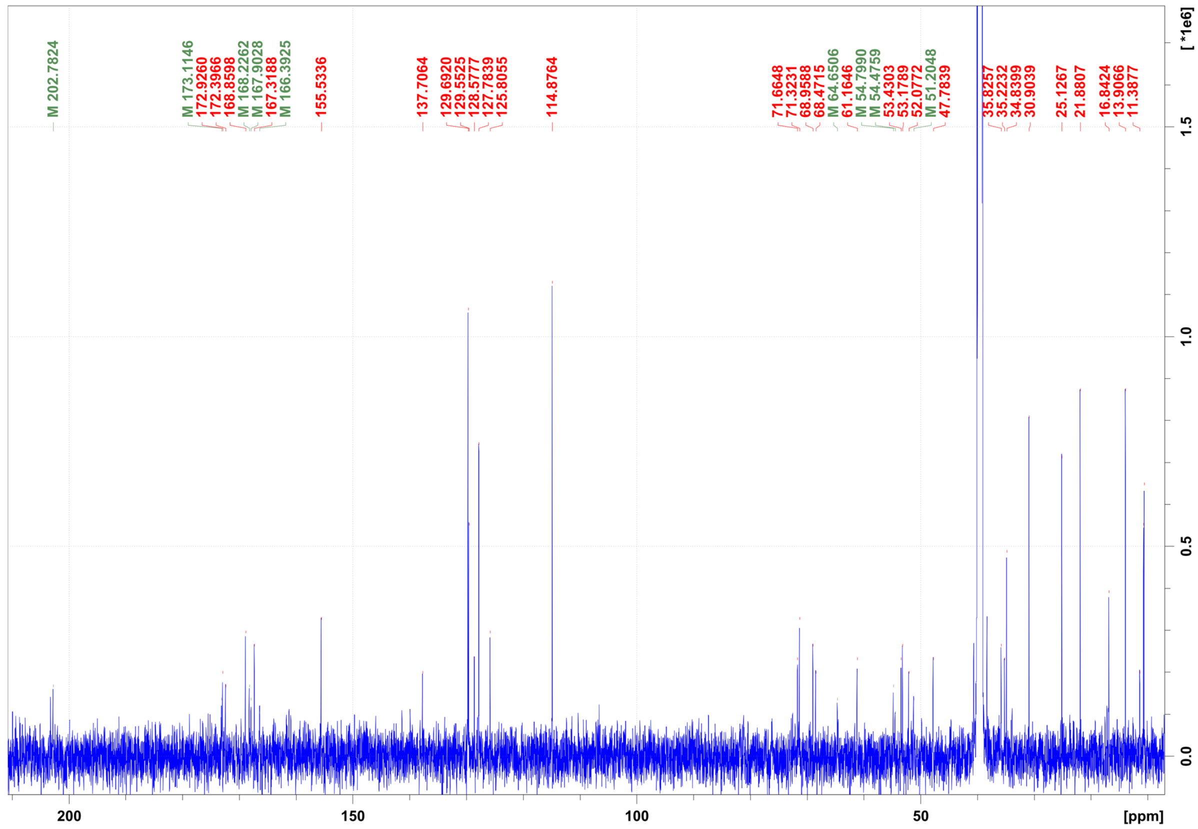


**Figure S58.** $^{13}C$ NMR spectrum of nostopeptolide KVJ3 (**7**) in DMSO-$d_6$ (150 MHz).

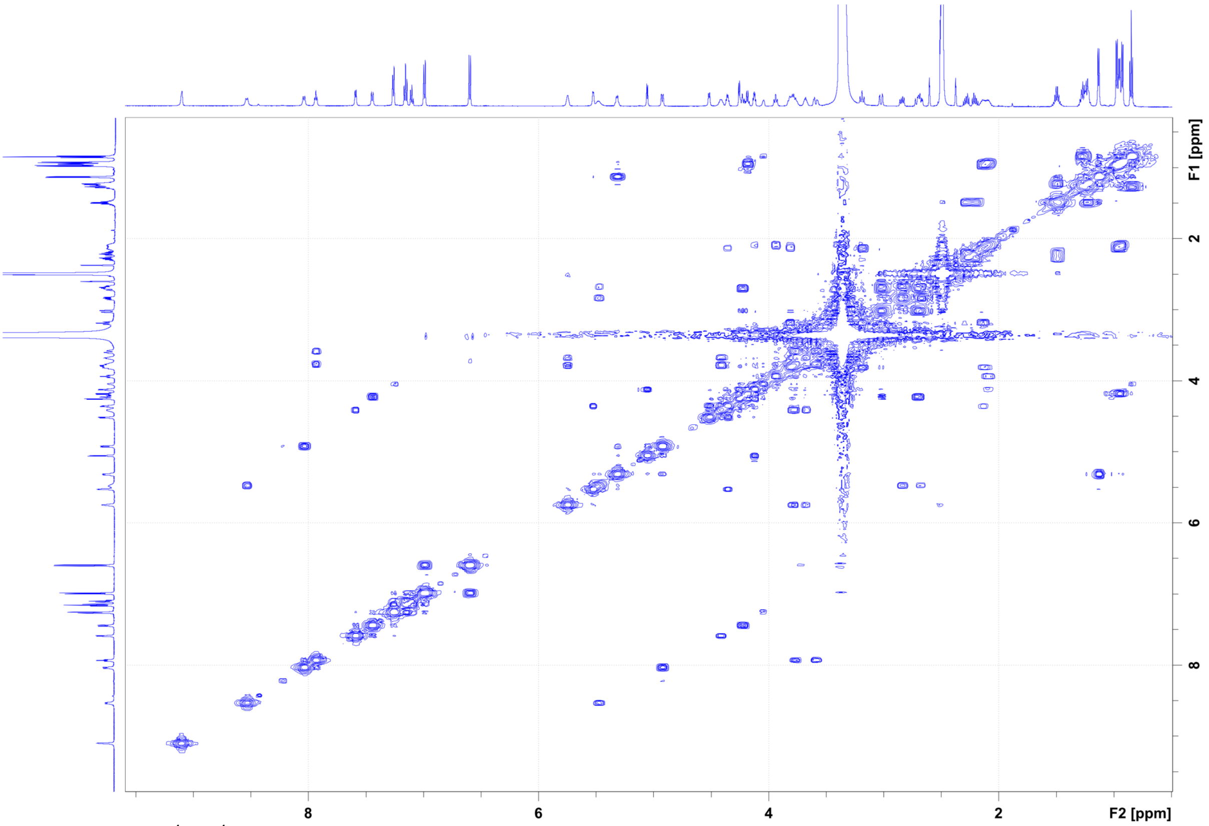


**Figure S59.** $^1H$-$^1H$ COSY spectrum of nostopeptolide KVJ3 (**7**) in DMSO-$d_6$ (600 MHz).

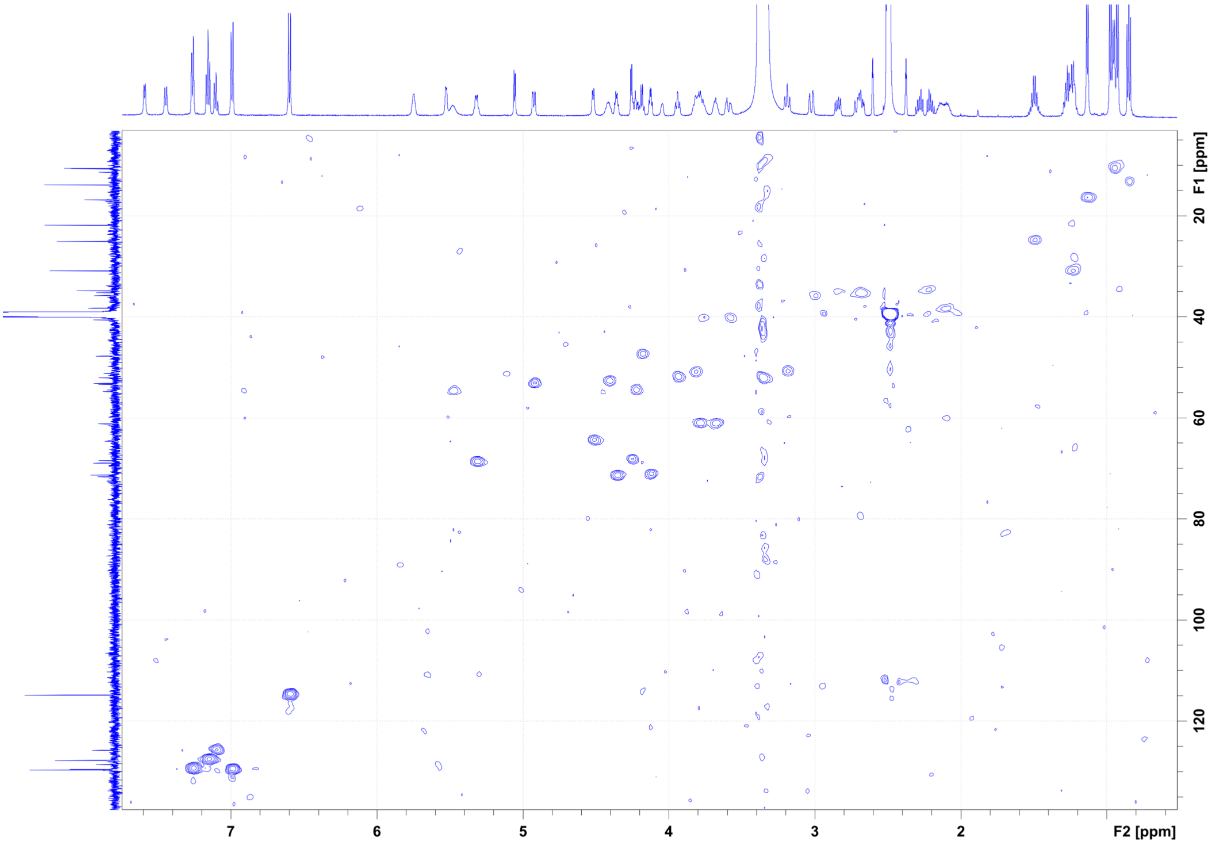


**Figure S60.** HSQC spectrum of nostopeptolide KVJ3 (**7**) in DMSO-$d_6$ (600 MHz).

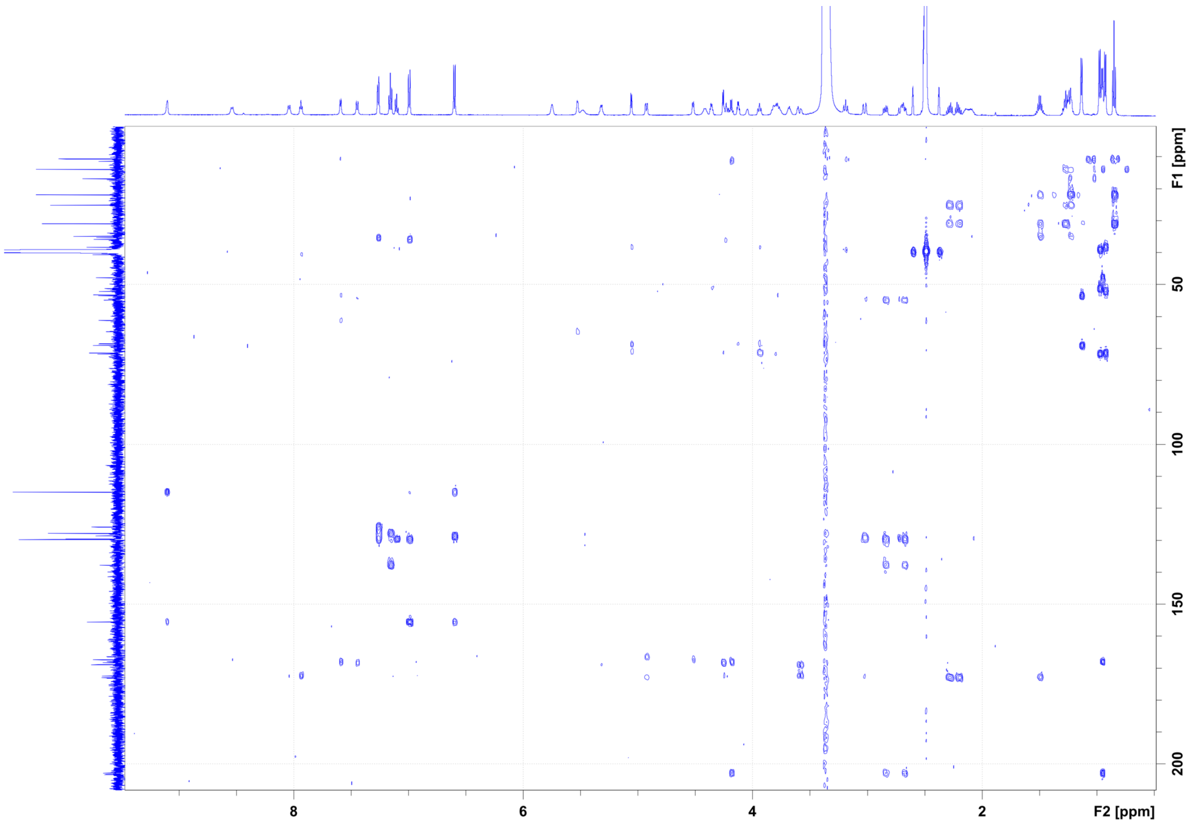


**Figure S61.** HMBC spectrum of nostopeptolide KVJ3 (**7**) in DMSO-$d_6$ (600 MHz).

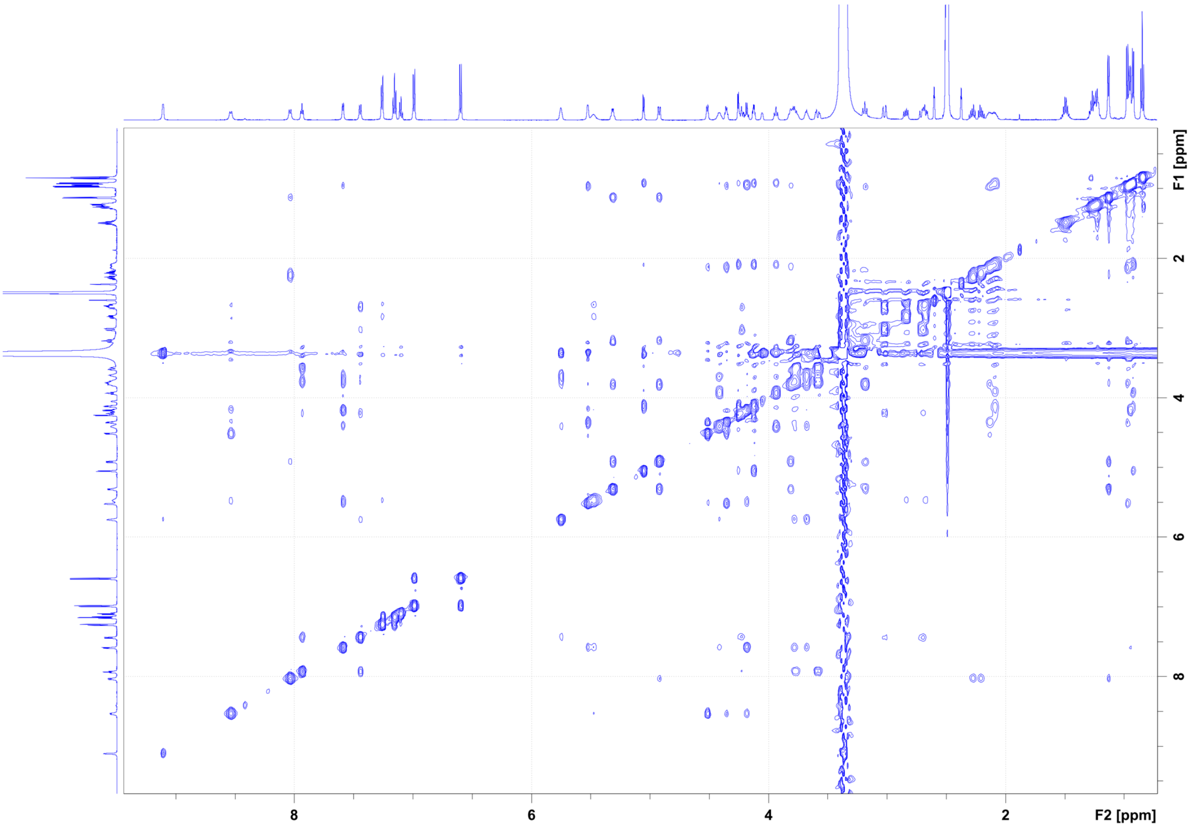


**Figure S62.** NOESY spectrum of nostopeptolide KVJ3 (**7**) in DMSO-$d_6$ (600 MHz).

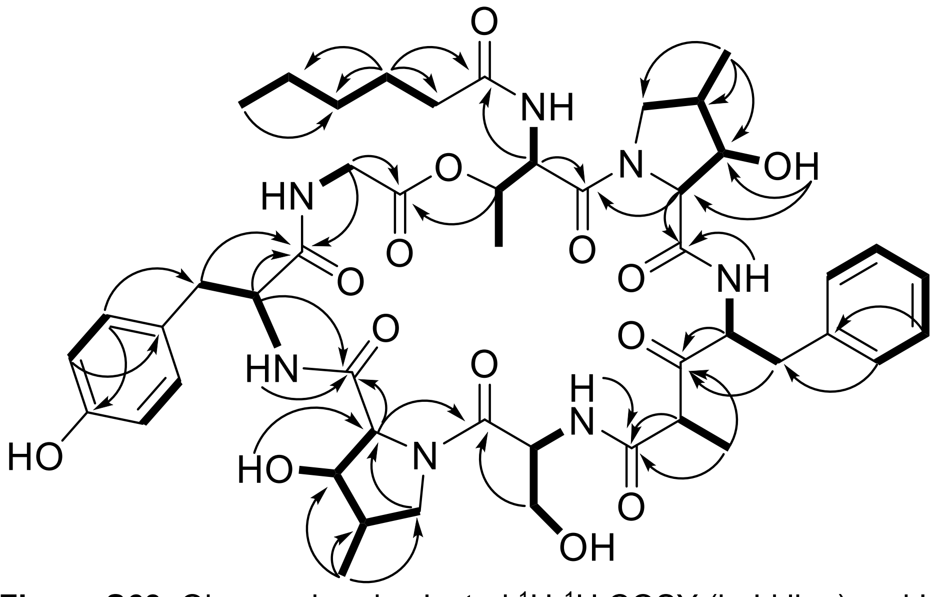


**Figure S63.** Observed and selected $^{1}H$-$^{1}H$ COSY (bold line) and HMBC (arrow) correlations of nostopeptolide KVJ3 (**7**).

**A)**

```
CLUSTAL O(1.2.4) multiple sequence alignment


MinB_C1           YPLSYGQQALWFLWQLAPESAAYNMVFACRICSQVNVTTLQNTFQLLINRHPQLRSTFSK 60
ClyF_C1_LEGE      YPLSYGQQAMWFLWQLAPESGFYNVAFTCRICSHVNVTTLQKTFQTLIERHPQLRSSFPK 60
ClyF_C1_PCC       YPLSYGQQAMWFLWQLAPENGFYNVAFTCRICSYVNVTTLQKTFQTLIERHPQLRSSFPK 60
                  *********:*********.. **:.*:***** *******:*** **:*******:* *

MinB_C1           QGNQVVQQIHQTQLPDFQQINASNWSEQELEQRVIQEYKQPFDLENGSVIRVRLFTSSPQ 120
ClyF_C1_LEGE      QGNKPVQQTHQTQVADFQQINASTWSEHELDHRVFQESQQPFDLENGSVMRVRLFTHSEQ 120
ClyF_C1_PCC       QGNKPVQKIHQTELPNFQQINASTWSDQELHHQVFQESQQPFDLENGSVMRVSLFTRSQQ 120
                  ***: **: ***:: :*******.**::**.::*:** :**********:** *** * *

MinB_C1           EHILLMSVHHIAGDGWSMPLLMEELITSYPALESGVQPSLTPLQHSYIDYIHWQRELLTT 180
ClyF_C1_LEGE      EHILLLTVHHIAIDAWCLPLLMEEMIMIYPALESGVQPPLTPLTNSYIDYVRWQRELLTS 180
ClyF_C1_PCC       EHILLLTVHHIAIDAWCLPLLMEEMIMTYPALESGVEPPLTPLKNSYIDYVRWQRELLTS 180
                  *****::***** *.*.:******:*  ********:* **** :*****::*******:

MinB_C1           VQGEKLWNYWQQQLAGELPVLNLPTDKPRPPIQTYNGASYNFTLSPELTEKLKKLAQTEG 240
ClyF_C1_LEGE      VPGEKLWNYWQKKLAGDLPVLNLPTDRPRPPIQTYNGASHRFTLSSKLTEQLKQLAQREG 240
ClyF_C1_PCC       TPGEKLWEYWQKKLAGDLPILNLPTDRPRPPIQTYNGASHRFTLSPKLTAQIKQLAQKEG 240
                  . *****:***::***:**:******:************:.**** :** ::*:*** **

MinB_C1           VTQYILLLAAFQVLLYRYTGQEDILVGVPTSGRTQIEFAPIVGYFVDPVVIRANL      295
ClyF_C1_LEGE      ATQSMVLLAAFQVLLYRYTGQEDILVGVPTSGRTKSEFMPLVGYFVDPVVMRVNF      295
ClyF_C1_PCC       ATQSMVLLAAFKILLHRYTGQEDILVGVPTSGRTKSEFMPLVGYFVDPVVMRVNF      295
                  .** ::*****::**:******************: ** *:*********:*.*:
```

**B)**

```
CLUSTAL O(1.2.4) multiple sequence alignment


ColG_C1      VYPLSYGQKNIWFLWQLAPQGHHYNLSFAIRIYSQADIITWQQTFKVLRQRHPLLRSTFP      60
MinB_C1      -YPLSYGQQALWFLWQLAPESAAYNMVFACRICSQVNVTTLQNTFQLLINRHPQLRSTFS      59
              *******: :********:.  **: ** ** **.:: * *:**::* :*** *****

ColG_C1      KVSEQAVLQVHQNQELDFLQIDATTWSEDELHKRVTEAHRHPFDLEKEPVMRVRCFTCSE      120
MinB_C1      KQGNQVVQQIHQTQLPDFQQINASNWSEQELEQRVIQEYKQPFDLENGSVIRVRLFTSSP      119
             * .:*.* *:**.*  ** **:*:.***:**.:** : :::*****:  *:*** **.*

ColG_C1      QEQVLLLTIHHIAWDGWSMNLIIKELPQLYQAQRAGVEASLPELKHSYQDYVRWQRELVE      180
MinB_C1      QEHILLMSVHHIAGDGWSMPLLMEELITSYPALESGVQPSLTPLQHSYIDYIHWQRELLT      179
             **::**:::**** ***** *:::**   * * .:**: **  *:*** **::*****:

ColG_C1      SQEGESLWDYWQQKLAGELPVLNLPTDRKRPPIQTYNGGSYPFKLSEKLTQQLKTLAQKE      240
MinB_C1      TVQGEKLWNYWQQQLAGELPVLNLPTDKPRPPIQTYNGASYNFTLSPELTEKLKKLAQTE      239
             : :**.**:****:*************: *********.** *.** :**::**.***.*

ColG_C1      DVTLYMLLLAVFQVLLSRYSGQKDVLVGSHTFGRSKAEFAPIVGYFVDSVVMRADL   296
MinB_C1      GVTQYILLLAAFQVLLYRYTGQEDILVGVPTSGRTQIEFAPIVGYFVDPVVIRANL   295
             .** *:****.***** **:**:*:***  * **:: *********** **:**:*
```

**Figure S64**. Clustal Omega multiple sequence alignments[17] of the condensation domain 1 of MinB (MinB_C1) with A) condensation domain 1 of ClyF from *Nodularia* sp. LEGE 06071 (ClyF_C1_LEGE) and condensation domain 1 of ClyF from *Anabaena* sp. PCC 7108 (ClyF_C1_PCC), and B) condensation domain 1 of (ColG_C1) indicates a closer relationship between MinB_C1 and ClyF_C1_LEGE or ClyF_C1_PCC than between MinB_C1 and ColG_C1, due to a higher degree of sequence similarity.

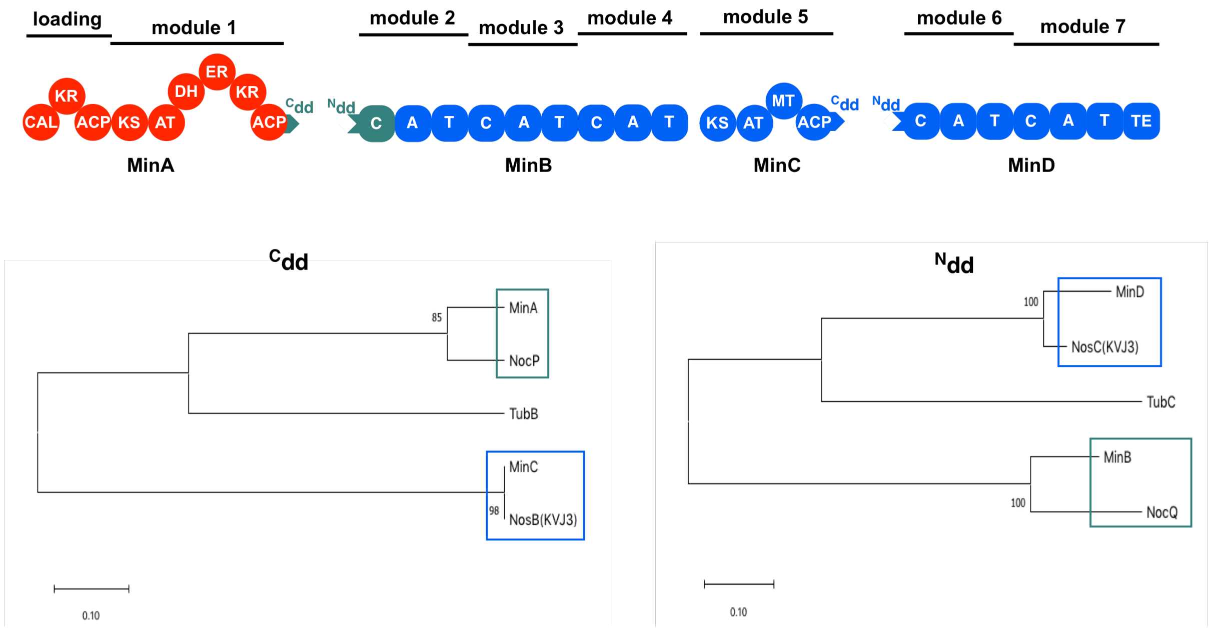


**Figure S65.** Docking domains in MinA-D. C- and N-terminal docking domains ($^{C}$dd; $^{N}$dd) can be found in MinA and MinB, which are closely related to the C- and N-terminal dds of NocP and NocQ. The relationship of dds, which are related to the prototype dds TubB_$^{C}$dd and TubC_$^{N}$dd, is far more pronounced to NocP_$^{C}$dd and NocQ_$^{N}$dd than to the dds of NosB and NosD or MinC and MinD.

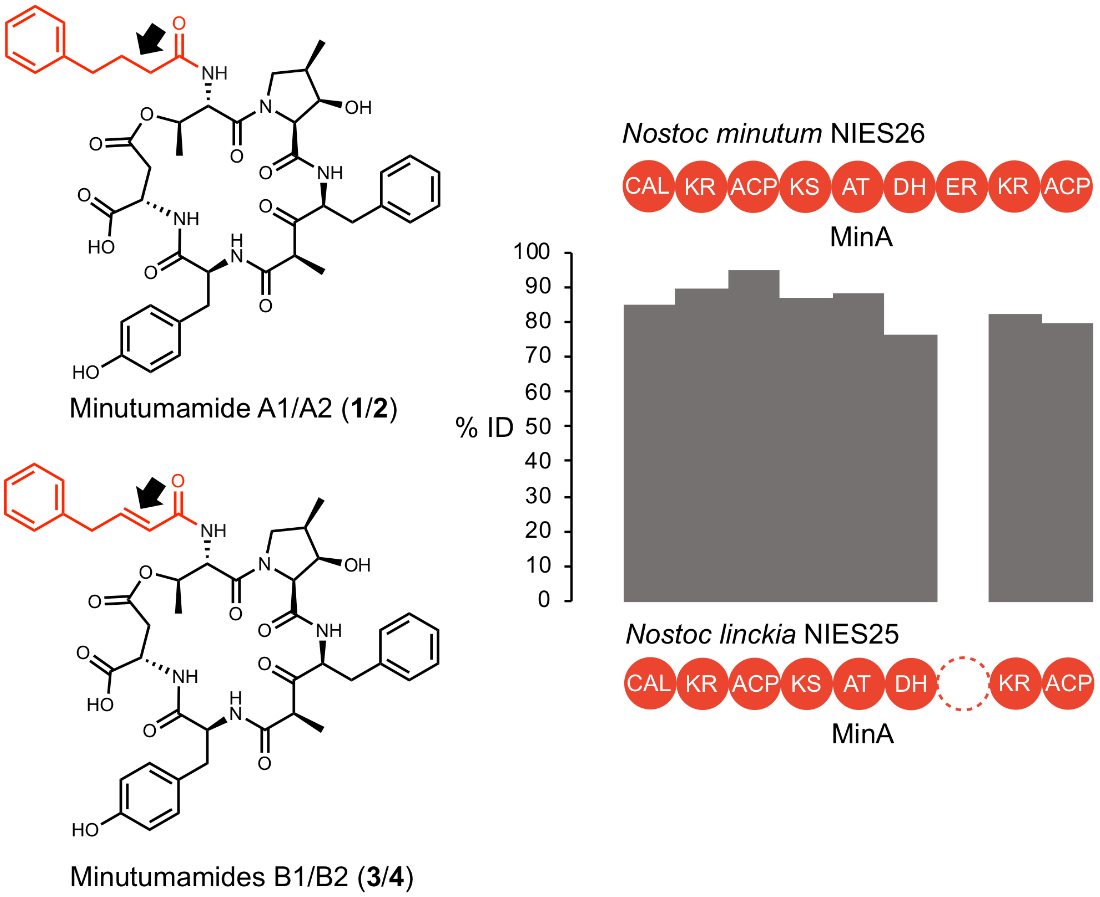


**Figure S66.** Diversification of minutumamides A1/A1 (**1**/**2**) and B1/B2 (**3**/**4**). MinA from *N. linckia* NIES25 lacks an enoyl reductase (ER) domain that is present in MinA from *N. minutum* NIES26, resulting in a double bond in minutumamide B1 and B2.

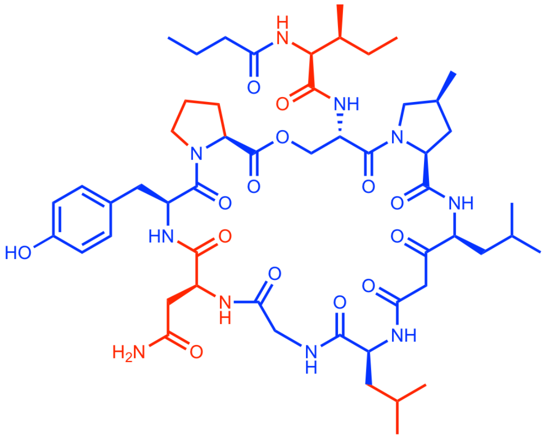

Nostopeptolide A1 (**5**)

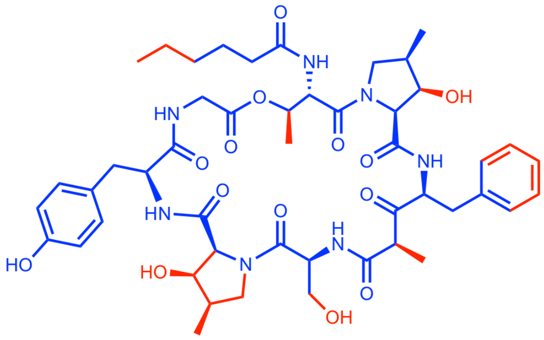

Nostopeptolide KVJ3 (**7**)

**Figure S67.** Structure comparison of nostopeptolide A1 (**5**) and nostopeptolide KVJ3 (**7**). Both compounds share similarity in the overall architecture, like the position of the macrolactone bond relative to the polyketide substructure, as well as shared structural features like the presence of a fatty acid moiety in the side chain. However, substantial differences exist between both compounds (marked in red), like the different ring topology (7-membered vs. 8-membered), the absence of an amino acid in the side chain of nostopetolide KVJ3 (**7**), as well as pronounced variation in the amino acid composition.

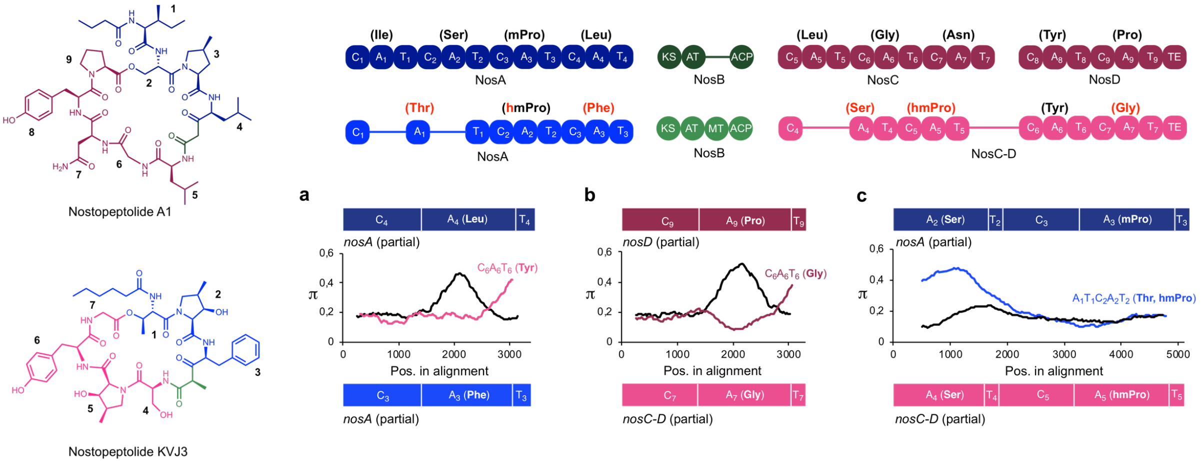


**Figure S68.** Comparison of gene segments that encode NRPS modules involved in nostopeptolide A1 (**5**) and nostopeptolide KVJ3 (**7**) biosynthesis by sliding window analysis to compute the average number of nucleotide differences per site between two sequences ($\pi$ values). a)-b) Pairwise comparison of modules reveals high divergence in the segments encoding the A domain, with sequences encoding the specificity pocket being the most affected. Remarkably, the divergent sequences can be complemented by highly homologous sequences from other modules of the two clusters, thereby indicating inter-cluster gene shuffling. c) The sequence encoding the domain series $A_4T_4C_4A_5T_5$, which facilitates the incorporation of serine and hydroxymethylproline in the biosynthesis of nostopeptolide KVJ3 (**7**) is highly homologous to the sequence encoding the domain series $A_2T_2C_2A_3T_3$, which facilitates the incorporation of serine and methylproline in the biosynthesis of nostopeptolide A1 (**6**).

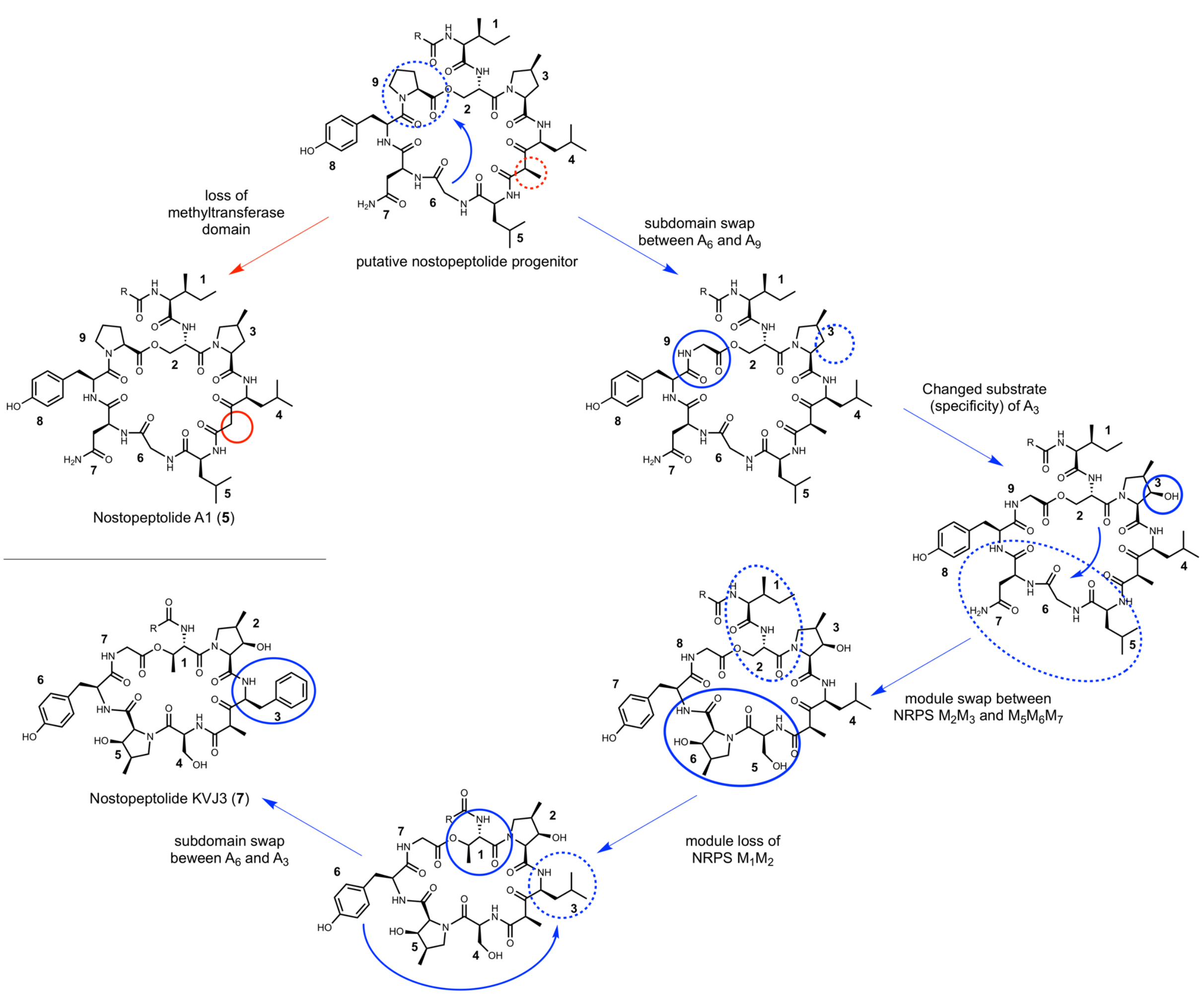


**Figure S69.** Evolutionary model for the emergence of nostopeptolide A1 (**5**) and nostopeptolide KVJ3 (**7**) from a putative nostopeptolide progenitor.

## References


[1] M. Kawachi, M. Ishimoto, F. Mori, K. Yumoto, M. Sato, M.-H. Noël, *9th Edition, Microbial Culture Collection at National Institute for Environmental Studies, Tsukuba, Japan* **2013**, 279.

[2] R. Rippka, *Methods Enzymol.* **1988**, *167*, 3-27.

[3] V. Toome, B. Wegrzynski, J. Dell, *Biochem. Biophys. Res. Commun.* **1976**, *71*, 598-602.

[4] J. Rozas, A. Ferrer-Mata, J. C. Sánchez-DelBarrio, S. Guirao-Rico, P. Librado, S. E. Ramos-Onsins, A. Sánchez-Gracia, *Mol. Biol. Evol.* **2017**, *34*, 3299-3302.

[5] S. Kumar, G. Stecher, M. Li, C. Knyaz, K. Tamura, *Mol. Biol. Evol.* **2018**, *35*, 1547-1549.

[6] B. O. Bachmann, J. Ravel, *Methods in enzymology* **2009**, *458*, 181-217.

[7] C. D. Richter, D. Nietlispach, R. W. Broadhurst, K. J. Weissman, *Nat. Chem. Biol.* **2008**, *4*, 75-81.

[8] N. Saitou, M. Nei, *Mol. Biol. Evol.* **1987**, *4*, 406-425.

[9] J. Felsenstein, *Evolution* **1985**, *39*, 783-791.

[10] E. Zuckerkandl, L. Pauling, *Evolutionary divergence and convergence in proteins*, Academic Press, New York, **1965**.

[11] P. Marfey, *Carlsberg Res. Commun.* **1984**, *49*, 591-596.

[12] J. M. Seco, E. Quiñoá, R. Riguera, *The Journal of organic chemistry* **1999**, *64*, 4669-4675.

[13] T. Golakoti, W. Y. Yoshida, S. Chaganty, R. E. Moore *Tetrahedron* **2000**, *56*, 9093-9102.

[14] G. A. Olah, Q. Wang, N. J. Trivedi, G. K. Surya Prakash, *Synthesis* **1991**, *1991*, 739-740.

[15] K. Moon, C. Lim, S. Kim, D. C. Oh, *J. Chromatogr. A* **2013**, *1272*, 141-144.

[16] N. J. Baxter, M. P. Williamson, *J. Biomol. NMR* **1997**, *9*, 359-369.

[17] F. Sievers, A. Wilm, D. Dineen, T. J. Gibson, K. Karplus, W. Li, R. Lopez, H. McWilliam, M. Remmert, J. Söding, J. D. Thompson, D. G. Higgins, *Mol. Syst. Biol.* **2011**, *7*, 539.
